# Accounting for Thermal Effects in the Charge-Transfer Properties of Dipolar Organic Dyes for Dye-Sensitized Solar Cells

**DOI:** 10.3390/molecules31193422

**Published:** 2026-09-26

**Authors:** Pierre C. Souza-Junior, Mateus R. Barbosa, Guilherme D. R. Matos, Flávio O. Sanches-Neto, João B. L. Martins, Daniel F. Scalabrini-Machado

**Affiliations:** 1 Laboratório de Modelagem de Sistemas Complexos, Instituto de Química, Universidade de Brasília, Brasília 70910-900, DF, Brazil; pierrecarlosj@gmail.com (P.C.S.-J.); mateusrodriguesbarbosa@gmail.com (M.R.B.); guilherme.matos@mcti.gov.br (G.D.R.M.); 2 Instituto Federal de Educação, Ciência e Tecnologia de Goiás, Goiânia 72876-601, GO, Brazil; flavio.neto@ifg.edu.br; 3 Grupo de Química Teórica e Estrutural de Anápolis, Centro de Pesquisa e Pós-Graduação, Universidade Estadual de Goiás, Anápolis 75132-400, GO, Brazil; 4 Laboratório de Química Computacional, Instituto de Química, Universidade de Brasília, Brasília 70910-900, DF, Brazil

**Keywords:** DSSC, thermal effects, conformational sampling, TD-DFT, donor–*π*–acceptor dyes, computational screening

## Abstract

Metal-free donor–π–acceptor (D–π–A) dyes are attractive, low-cost sensitizers for dye-sensitized solar cells (DSSC), and computational screening allows rapid exploration of this vast chemical space. However, most screening protocols characterize each candidate from a single low-energy conformer, neglecting the conformational flexibility that these dyes retain at typical operating temperatures. Here we present a thermally aware screening framework that combines CREST conformational sampling with CENSO ensemble refinement (r^2^SCAN-3c) and Boltzmann-weighted TD-DFT (CAM-B3LYP) descriptors to evaluate 150 D–π–A architectures built from five donors, ten π-bridges, and three acceptors. A composite *Z*-score ranking of five charge-transfer and photovoltaic descriptors selected the ten most promising sensitizers, which were further examined across three functionals (B3LYP, CAM-B3LYP, ωB97XD), charge-transfer kinetics, and explicit anchoring onto a (${\mathrm{TiO}}_{2}{)}_{14}$ anatase cluster. Boltzmann-averaged and single-conformer descriptors differed most strongly for dyes in which the lowest-energy conformers had very low oscillator strengths, making the ensemble properties sensitive to higher-energy, more emissive conformers. For D4-B1-A1, for example, the difference in the idealized short-circuit photocurrent descriptor $J_{\mathrm{SC}-\max}$ approached 100%, while the average deviation across the dataset was 18%. A similarly pronounced difference was observed for the charge-transfer rate $k_{\mathrm{CT}}$ for the same sensitizer, with the trend persisting across two additional functionals. These results suggest that conformational effects can become particularly important when torsional motions involving the donor or acceptor groups substantially alter the electronic character of the D–π–A sensitizer. Thus, for architectures with appreciable donor–bridge or bridge–acceptor torsional flexibility, ensemble averaging may provide a more representative description than relying on a single low-energy conformer. Anchoring calculations further showed that dyes bearing the dicyanomethylene-rhodanine acceptor adsorb only weakly onto TiO_2_, despite favorable electronic descriptors. These results establish conformational averaging as a useful component of computational DSSC screening for structurally flexible sensitizers.

## 1. Introduction

Photovoltaic technology spans crystalline and thin-film silicon, inorganic thin-film absorbers, hybrid organic–inorganic perovskites, quantum-dot sensitizers, and metal-oxide-based dye-sensitized solar cells (DSSCs), each offering a distinct balance of efficiency, cost, and manufacturing complexity. Metal oxides such as TiO_2_, ZnO, and WO_3_ recur across these technologies as electron-transport layers, photoanodes, or passivation layers [1], and quantum-dot devices have advanced rapidly through both direct quantum-dot sensitization [2] and perovskite quantum dots that combine size-tunable absorption with the optoelectronic properties of the perovskite family [3]. Within this landscape, DSSCs occupy a niche defined by comparatively simple, low-temperature fabrication and by their reliance on a molecular rather than purely inorganic light absorber, making rational sensitizer design a central lever for device performance. These devices offer potentially low-cost fabrication, and organic dyes have become central to realizing that promise [4,5,6], although electrolyte stability, solvent choice, and dye source substantially affect device lifetime and environmental burden [7,8]. Since the pioneering work of O’Regan and Grätzel [9], ruthenium-based complexes drove DSSCs to certified efficiencies approaching 10% [10], but the scarcity and toxicity of ruthenium motivated a shift toward metal-free alternatives. Organic donor–π–acceptor (D–π–A) dyes have emerged as the leading class of such alternatives, owing to their synthetic versatility, strong light absorption, and large molar extinction coefficients [11,12]. In this modular architecture, the donor, conjugated bridge, and acceptor can be systematically modified to tune the frontier-orbital energies and charge-transfer character of the sensitizer. Upon photoexcitation, these electronic characteristics govern the redistribution of charge through the conjugated bridge, influencing the subsequent photoconversion processes.

Theoretical screening has played an important role in identifying donor, bridge, and acceptor motifs with favorable electronic and photophysical characteristics. DFT and TD-DFT investigations have systematically compared electron-donating groups, including triarylamine, heteroaromatic, and phenoxazine-based donors, for their ability to red-shift absorption and stabilize charge-separated excited states [13,14,15,16]. Comparative studies of electron-withdrawing anchoring groups, particularly cyanoacrylic- and carboxylic-acid-derived groups, have demonstrated that the choice of anchoring group can influence both light-harvesting efficiency and interaction with the TiO_2_ surface [17,18]. Other theoretical studies have investigated D–π–A sensitizers incorporating diverse combinations of donor, π-bridge, and acceptor components, including porphyrin- and perylene-based acceptors, norbornylogous spacers, and linear-carbon-chain bridges [19,20,21].

At the theoretical level, assessing a candidate sensitizer requires a compact set of electronic and photovoltaic descriptors. The alignment of the dye’s frontier-orbital energies with the iodide/triiodide redox couple and the TiO_2_ conduction band provides a first approximation to the thermodynamic feasibility of electron injection and dye regeneration. At the same time, the oscillator strength of the S_0_→S_1_ transition contributes directly to the estimated light-harvesting efficiency (LHE) [22,23]. From these electronic-structure quantities, photovoltaic descriptors such as the open-circuit voltage ($V_{OC}$), the electron-injection driving force ($\Delta G_{\mathrm{inj}}$), and the dye-regeneration driving force ($\Delta G_{\mathrm{reg}}$) can be estimated directly from ground- and excited-state calculations [19,20]. The spatial separation between the photoexcited hole and electron densities provides an additional descriptor of charge-transfer (CT) character and can help distinguish spatially separated excitations from more localized transitions. Because every one of these descriptors is computed directly from the dye’s molecular geometry, their accuracy is inherently tied to how faithfully that geometry represents the sensitizer under operating conditions. TD-DFT excitation energies computed with functionals of the family employed here reproduce experimentally measured absorption maxima of triphenylamine-based D–π–A dyes to within a few tenths of an eV [24,25].

Dye-sensitized solar cells (DSSCs) operate at finite temperatures, typically reaching 60–80 °C under prolonged solar irradiation [26]. For a flexible sensitizer, however, temperature is not only an operational variable. It also determines the distribution of molecular conformations that are thermally populated at equilibrium. A dye with several low-energy conformers therefore cannot, in general, be represented by a single optimized structure. Instead, its observable properties reflect a Boltzmann-weighted ensemble whose composition depends on the relative free energies of the accessible conformers. This distinction is particularly relevant for D–π–A sensitizers, in which rotation about donor–bridge and bridge–acceptor bonds can modify orbital localization, frontier-orbital energies, excitation energies, and charge-transfer character. Thus, even without irreversible thermal degradation, finite-temperature conformational sampling can affect the molecular descriptors used to rank candidate dyes.

Nevertheless, computational screening of DSSC sensitizers is still predominantly based on properties calculated for a single optimized geometry [13,14,15,16,17,18,19,20,21,22,23]. This approach implicitly assumes that one structure adequately represents the dye under the conditions relevant to its operation. This assumption may be reasonable for rigid chromophores, but becomes less reliable as conformational flexibility increases. A few studies have explicitly incorporated conformational or finite-temperature sampling. CREST/GFN2-xTB sampling followed by Boltzmann-weighted TD-DFT has been used in the design of D–π–A candidates [27], while molecular-dynamics sampling combined with TD-DFT has been employed to obtain ensemble-averaged spectra of flexible dyes in solution [28]. Ab initio molecular dynamics has also provided a detailed description of solvent organization and nuclear motion in the prototypical Ru(II) sensitizer [Ru(dcbpy)_2_(NCS)_2_]^4−^ [29]. However, the computational cost of such an approach limits its direct application to large molecular libraries [30].

The challenge for computational screening is therefore not simply to sample conformations, but to do so at a cost that allows evaluation of many candidate structures. Here, we combine metadynamics-based sampling at the GFN2-xTB level with DFT refinement to efficiently explore thermally accessible conformations before evaluating their electronic properties. Metadynamics enhances exploration of conformational space by discouraging repeated sampling of already visited regions, making it particularly useful when relevant conformers are separated by torsional barriers that may hinder their interconversion during conventional molecular dynamics [31]. The resulting conformational ensemble can then be refined at the DFT level, providing a practical compromise between conformational coverage and electronic-structure accuracy. This strategy enables consistent ensemble-based analysis across a library of chemically distinct sensitizers.

In this work, we investigate how finite-temperature conformational ensembles influence the descriptors used in computational DSSC screening. We construct a library of 150 systematically combined D–π–A dyes spanning bridges with contrasting structural characteristics and evaluate their thermally accessible conformers using the same computational workflow. For each dye, we compare properties obtained from the conventional single optimized structure with the corresponding Boltzmann-weighted ensemble averages. This enables us to determine when conformational averaging substantially changes the descriptors used for screening and whether the magnitude of this effect depends systematically on molecular architecture. More broadly, the study provides a conformationally informed and computationally tractable framework for screening D–π–A sensitizers at finite temperature.

## 2. Results and Discussion

### 2.1. First Screening of the Chromophores (Gas Phase)

The modular design yielded 150 D–π–A structures, represented in Figure 1 for comparing charge-transfer behavior and photovoltaic properties.

The gas-phase screening revealed broad variation in the charge-transfer and photovoltaic descriptors across the 150-dye library. The hole–electron descriptors distinguished spatially separated and more localized excitations (Figures S1–S6 in the Supporting Information), while the terminal acceptor strongly influenced the LUMO energy (Figures S11 and S12) and $V_{OC}$ (Figures S15 and S16), with A1 generally yielding the most favorable values, followed by A2 and A3. Most dyes combined visible-light absorption (Figures S7 and S8) with high LHE (Figures S13 and S14), and the most favorable architectures balanced electron-injection (Figures S17 and S18) and dye-regeneration driving forces (Figures S19 and S20), showing that the donor–bridge–acceptor combination can tune both optical response and charge-transfer energetics.

The difference between the Boltzmann average and the most-populated conformer was strongly property-dependent. The HOMO energy showed only minor changes between the most-populated conformer and the Boltzmann average (Figures S9 and S10), whereas $J_{\mathrm{SC}\text{-}\max}$ changed by 12.5% on average and by up to 100% when the most-populated conformer was essentially dark (Figures S21 and S22). The very large relative deviations found for the *t* index (Figures S5 and S6) and $\Delta G_{\mathrm{inj}}$ (Figures S17 and S18) in some dyes primarily arose from Boltzmann-averaged values close to zero rather than from comparably large absolute changes. Moreover, these deviations correlated only weakly with either the dominant conformer’s Boltzmann weight or the ensemble size, so neither quantity reliably identifies when a single-conformer description will depart from the thermal average. The subsequent selection therefore used the Boltzmann-averaged descriptors, while the full conformer-resolved comparisons are reported in the Supporting Information.

### 2.2. Composite-Score Selection of the Top-Ranked Sensitizers

Application of the composite *Z*-score ranking procedure described in Section 3.4 to the 150 generated molecular architectures identified ten top-ranked sensitizers: D5-B7-A3, D3-B7-A3, D4-B5-A2, D4-B1-A2, D5-B7-A2, D2-B1-A1, D3-B2-A1, D3-B1-A1, D4-B1-A1, and D3-B2-A3. Because the composite score integrates the descriptors $S_{r}$, *D*, *t*, $\lambda_{\max}$, and *f*, these structures were selected as those providing the most favorable overall balance between excited-state charge separation and optical response within the initial screening.

To assess the robustness of this selection with respect to the computational method and environment, the ten top-ranked sensitizers were subsequently evaluated using three density functionals, B3LYP, CAM-B3LYP, and ωB97XD, with implicit solvation in acetonitrile. In addition, CAM-B3LYP calculations were performed in the gas phase to isolate the effect of the environment. The resulting charge-transfer descriptors, frontier-orbital energies, and photovoltaic parameters are summarized in Figure 2.

The calculated descriptors exhibit a pronounced dependence on the choice of exchange-correlation functional (Figure 2). Under implicit solvation, B3LYP consistently predicts the smallest $S_{r}$ values, together with the largest *D* and *t* values, indicating a more pronounced charge-transfer character than CAM-B3LYP and ωB97XD. In contrast, ωB97XD generally yields the largest $S_{r}$ and the smallest *D* and *t*, corresponding to a more localized excited-state description, while CAM-B3LYP gives intermediate values for most structures. The greater CT character predicted by B3LYP is consistent with the known limitations of global hybrid functionals for long-range charge-transfer excitations, for which their limited long-range exact-exchange contribution can lead to underestimated excitation energies [32]. Range-separated functionals such as CAM-B3LYP and ωB97XD provide a more appropriate treatment of long-range exchange and are therefore expected to offer a more reliable description of the CT states considered here [33]. Accordingly, B3LYP results are retained for comparison, whereas the physical interpretation below focuses primarily on CAM-B3LYP and ωB97XD.

The molecular environment also influences the calculated descriptors, although the magnitude and direction of the response depend on the sensitizer architecture. Relative to the gas-phase CAM-B3LYP results, implicit acetonitrile generally increases $S_{r}$ and decreases *t*, indicating a reduction in the calculated CT character, while D4-B1-A1 shows the opposite trend. Similar architecture-dependent effects are observed for the photovoltaic and thermodynamic descriptors, where solvation modifies $V_{\mathrm{OC}}$ and generally makes $\Delta G_{\mathrm{inj}}$ less negative, although D4-B1-A1 and D4-B1-A2 exhibit more favorable injection energetics in solution. The LHE is also affected by the molecular environment, with D4-B1-A1 showing a particularly pronounced solvent response. These results indicate that solvent effects cannot be represented by a uniform correction across the sensitizer library and should instead be considered together with the molecular architecture.

The frontier-orbital energies preserve the qualitative alignment required for electron injection and dye regeneration across the methods examined: the LUMO energies remain above the TiO_2_ conduction-band reference, whereas the HOMO energies remain below the $I^{-}/I_{3}^{-}$ redox potential. Nevertheless, their absolute values vary substantially with the functional. The same method dependence is reflected in $V_{\mathrm{OC}}$, for which ωB97XD consistently predicts the largest values, followed by CAM-B3LYP and B3LYP, with differences approaching 2 eV for some sensitizers. The LHE also varies substantially among the functionals because $\mathrm{LHE}=1-10^{-f}$ and therefore directly reflects differences in the calculated oscillator strengths. ωB97XD generally predicts high LHE values, whereas B3LYP gives substantially lower values for several sensitizers, with D4-B5-A2 providing a particularly clear example.

The thermodynamic descriptors further illustrate the functional dependence of the predicted photovoltaic performance. ωB97XD generally predicts the largest $\Delta G_{\mathrm{reg}}$ values but the least favorable $\Delta G_{\mathrm{inj}}$ values among the three functionals. Positive $\Delta G_{\mathrm{inj}}$ values are obtained for D3-B2-A1, D3-B2-A3, and D4-B1-A1, indicating thermodynamically unfavorable electron injection according to the adopted energetic model, whereas the remaining sensitizers retain negative values. Overall, the comparison demonstrates that both the electronic-structure method and the molecular environment substantially affect the descriptors used to evaluate the sensitizers. B3LYP systematically predicts more pronounced CT character and consequently alters several optical and photovoltaic descriptors, whereas CAM-B3LYP and ωB97XD provide less pronounced CT character within the present analysis. These results highlight the importance of using range-separated functionals and an appropriate treatment of the molecular environment when assessing the top-ranked sensitizers for DSSC applications.

### 2.3. Kinetic Analysis of Charge-Transfer Constants

The charge-transfer kinetics of the ten top-ranked sensitizers were evaluated using the three density functionals considered above. Full per-conformer values and Boltzmann averages for all ten sensitizers ($S_{r}$, *D*, *t*, HOMO, LUMO, HOMO–LUMO gap, $\lambda_{\max}$, *f*, [E(M)], IP, EA, $\lambda_{e}$, $\lambda_{h}$, $\lambda_{i}$, $\mu_{1}$, $\mu_{2}$, $\mu_{12}$, $V_{12}$, and $k_{\mathrm{CT}}$ at each of the three functionals) are reported in Tables S2–S7 of the Supporting Information. Reorganization energies show comparatively moderate functional dependence, whereas $V_{12}$ and $k_{\mathrm{CT}}$ vary substantially with the functional. D4-B5-A2 illustrates this sensitivity most clearly, with $V_{12}$ increasing from 0.010 eV with B3LYP to 1.924 eV with ωB97XD (1.842 eV with CAM-B3LYP) and $k_{\mathrm{CT}}$ rising from effectively zero to $1.073\times 10^{13}\;s^{-1}$ with CAM-B3LYP. B3LYP generally yields lower $k_{\mathrm{CT}}$ values than CAM-B3LYP and ωB97XD, although the magnitude of this dependence varies among the molecular architectures.

The idealized short-circuit photocurrent density, $J_{\mathrm{SC}-\max}$, calculated from the $S_{0}\to S_{1}$ transition also varies substantially with molecular structure and functional, ranging from 0.01 to 15.16 mA cm^−2^ across the ten sensitizers. D4-B5-A2 again shows the clearest sensitivity, with $J_{\mathrm{SC}-\max}=0.01$ mA cm^−2^ at the B3LYP level compared with 0.91 and 0.57 mA cm^−2^ for CAM-B3LYP and ωB97XD, respectively, while D3-B2-A3 gives the largest calculated value (15.16 mA cm^−2^ with CAM-B3LYP). This pronounced functional dependence arises from a qualitative difference in the predicted $S_{0}\to S_{1}$ transition rather than from differences in the conformational ensemble. In four of the ten sensitizers (D3-B7-A3, D4-B5-A2, D5-B7-A2, and D5-B7-A3), B3LYP predicts a very weak S_0_→S_1_ transition for the same conformers that exhibit substantially larger oscillator strengths with CAM-B3LYP and ωB97XD. The ensemble-averaged oscillator strength is below 0.03 at B3LYP, compared with values above 0.29 for both range-separated functionals. Moreover, the low B3LYP intensity is not restricted to the most-populated conformer, with f<0.05 for every conformer considered for these four dyes. The simulated spectra in Figures S23 and S24 of the Supporting Information are consistent with this behavior. At B3LYP, S_1_ contributes little to the absorption in the relevant spectral region, whereas at CAM-B3LYP and ωB97XD it contributes substantially to the main absorption band. The corresponding changes in $S_{r}$, *D*, and *t*, including a reversal of the *t* index for several dyes, show that the choice of functional can substantially alter the electronic interpretation of the same sensitizer.

To assess the importance of Boltzmann averaging, we compared the properties of the single most-populated conformer with the corresponding ensemble average for each sensitizer. For $J_{\mathrm{SC}-\max}$ at the CAM-B3LYP level, this comparison yields a mean absolute deviation of 18.4% across the ten sensitizers, reaching 99.9% for D4-B1-A1 (Figure 3). The effect is more pronounced for $k_{\mathrm{CT}}$, where deviations at the CAM-B3LYP level range from −99.95% (D4-B1-A1) to +6.3% (D3-B1-A1), with the remaining sensitizers staying within about ±25% of their Boltzmann-averaged value (Figure 4). In both cases, D4-B1-A1 shows a near-suppression of the calculated property when only its dominant conformer is considered, because that conformer is essentially optically dark; the ensemble average instead reflects minority conformers with substantial oscillator strength and electronic coupling. Repeating the comparison at the B3LYP and ωB97XD levels reproduces the same qualitative pattern for both $J_{\mathrm{SC}-\max}$ (Figures S27 and S28) and $k_{\mathrm{CT}}$ (Figures S29 and S30), confirming that this sensitivity to conformer selection is not restricted to a single electronic-structure method. Only three of the ten sensitizers exhibit $|\Delta k_{\mathrm{CT}}|>10\%$ relative to the Boltzmann average at the CAM-B3LYP level.

Separating the contributions of $\lambda_{i}$ and $V_{12}$ to this sensitivity (Figures S25 and S26) shows that $\lambda_{i}$ is essentially insensitive to conformer selection, with a mean absolute deviation of only 0.8%, while $V_{12}$ accounts for most of the variation, averaging 13.0% and reaching −91.9% for D4-B1-A1. The electronic coupling is therefore the dominant source of the conformational sensitivity of $k_{\mathrm{CT}}$ within the present model.

The origin of the largest deviations differs between D4-B1-A1 and the D5-B7-A2/D5-B7-A3 pair. For D4-B1-A1, the two most-populated conformers (82.5% of the population) are essentially optically dark, so the Boltzmann-averaged $k_{\mathrm{CT}}$ is instead carried by a minority bright conformer with an unusually small hole reorganization energy. For D5-B7-A2 and D5-B7-A3, no conformer is dark, but the most-populated conformer in each case has the largest excited-state dipole shift and the smallest transition dipole of its ensemble, which mechanically lowers $V_{12}$ through Equation (6). The magnitude of the best-conformer-versus-average discrepancy is thus governed by the electronic heterogeneity of the low-lying conformers rather than by ensemble size.

Collectively, these results show that conformational averaging can materially affect both the optical response and charge-transfer kinetics of the selected D–π–A sensitizers, particularly when higher-energy conformers differ electronically from the dominant one. Boltzmann-averaged properties therefore provide a more representative basis for comparing sensitizers with thermally accessible conformational ensembles than properties obtained from a single conformer.

### 2.4. TiO_2_-Dye Anchoring

The strength and structural details of the dye–TiO_2_ interaction were next assessed for the ten top-ranked sensitizers, using the ${\left({\mathrm{TiO}}_{2}\right)}_{14}$ cluster model and bidentate anchoring protocol described above. Table 1 reports the raw and BSSE-corrected interaction energies of each dye–${\left({\mathrm{TiO}}_{2}\right)}_{14}$ complex, together with the dipole moment of the isolated dye and of the anchored complex, whereas Figure 5 presents the corresponding optimized geometries, annotated with these energies and dipole moments.

Figure 5 shows that, in all nine optimized structures, the dye’s acceptor group binds directly to the cluster surface, coordinating to one or two surface Ti atoms depending on the acceptor chemistry. To quantify this interaction, we measured, in each complex, the distance between the dye’s anchoring heteroatom(s) and the nearest surface titanium atom. For the seven dyes bearing the carboxylic-acid-derived acceptors (A1, A2), both oxygen atoms of the anchoring group remained bonded to two distinct Ti atoms, with O–Ti distances of 1.99–2.14 Å, confirming the bidentate binding mode assumed for these systems [21,34]. The two remaining A3 dyes anchor instead through the nitrogen atom(s) of the dicyanovinyl group. D5-B7-A3 anchors through both nitrogen atoms, with N–Ti distances of 2.17 Å, whereas D3-B7-A3 retains only one N–Ti contact within bonding distance (2.20 Å), indicating a monodentate rather than bidentate attachment.

This weaker and geometrically distinct A3 anchoring motif is consistent with the interaction-energy trends in Table 1: D3-B7-A3 and D5-B7-A3 exhibit the two weakest BSSE-corrected interactions of the series, at −12.41 and −20.03 kcal mol^−1^, respectively, substantially less negative than the −39 to −62 kcal mol^−1^ range found for the A1/A2 dyes. This behavior is consistent with the absence of negatively charged oxygen donors in A3 and with the weaker coordination of neutral nitrile nitrogen atoms relative to carboxylate oxygen donors under the present model. Beyond interaction strength, Table 1 reveals that anchoring does not produce a uniform change in the molecular dipole moment. Upon binding to TiO_2_, the dipole moment increases for four sensitizers, most notably D2-B1-A1 (from 20.84 to 35.82 D, +72%) and D4-B1-A1 (from 22.79 to 25.53 D, +12%), but decreases for the remaining six, with reductions as large as 59% for D4-B1-A2. Thus, surface anchoring does not produce a uniform increase in the molecular dipole moment across the sensitizer series. Instead, the change in dipole moment depends on the electronic structure and anchoring geometry of each dye, indicating that charge redistribution upon TiO_2_ binding can either reinforce or partially oppose the pre-existing donor–acceptor polarization within the sensitizer.

## 3. Methods

### 3.1. Construction of the Molecular Test Set

We constructed a chemically diverse yet structurally controlled test set of 150 D–π–A sensitizers by combining five donor units (D), ten conjugated bridges (B), and three acceptor groups (A) commonly employed in DSSC investigations. The donor units consist primarily of aromatic nitrogen-containing groups commonly used in push–pull chromophores [13,14,15,16,17]. The bridges were selected to provide contrasting molecular lengths while maintaining symmetric connectivity. Each structure contained a single bridge so the spacer’s effect could be evaluated independently. The acceptors were selected to represent distinct electron-withdrawing and anchoring motifs, including carboxylic-acid-derived and dicyanomethylene/rhodanine groups, enabling comparison of their effects on electronic properties and TiO_2_ binding [17,18].

### 3.2. CRENSO Protocol for Conformational and Photophysical Characterization

To account for the sensitivity of electrochemical properties to molecular geometry, we characterized the thermal conformational ensembles using the CRENSO protocol, which combines CREST conformational sampling with CENSO ensemble refinement [35]. Initial conformers were sampled and optimized at the GFN-FF/GFN2-xTB levels using CREST [36], and the resulting ensembles were refined at the r^2^SCAN-3c level [37] using CENSO [35] and ORCA 5 [38]. For the conformational ensembles used in thermal averaging, the energy windows applied during CREST sampling and CENSO refinement were each reduced by 0.5 kcal mol^−1^ relative to their respective default values.

To reduce the overall computational cost, the photophysical properties of all 2960 CENSO-refined conformers were evaluated using the Tamm–Dancoff approximation [39], as implemented in Gaussian 16 [40], at the CAM-B3LYP/6-31G(d,p) level for the first 12 excited states. Multiwfn 3.8 [41,42] was used to extract the relevant electronic and excited-state descriptors. For each dye, the electronic and S_0_→S_1_ transition descriptors of the individual conformers were Boltzmann-weighted at 298.15 K using their relative Gibbs free energies, according to $p_{i}=e^{-\Delta G_{i}/RT}/\sum_{i}e^{-\Delta G_{i}/RT}$. We then calculated the ensemble-level photovoltaic parameters for each dye from these per-conformer electronic descriptors and this procedure yielded one set of thermally corrected photovoltaic parameters for each of the 150 dyes.

### 3.3. Photovoltaic Parameters

To assess the photovoltaic characteristics of each sensitizer at the screening level, we selected four complementary parameters that describe light harvesting and the energetics of the charge-transfer cycle, namely the open-circuit voltage ($V_{OC}$), light-harvesting efficiency (LHE), electron-injection driving force ($\Delta G_{\mathrm{inj}}$), and dye-regeneration driving force ($\Delta G_{\mathrm{reg}}$). The $V_{OC}$ reflects the energy alignment between the dye’s LUMO and the semiconductor conduction band, whereas LHE estimates the fraction of incident light absorbed by the sensitizer. The two driving forces assess whether the excited dye can inject an electron into the semiconductor and whether the redox mediator can subsequently regenerate the resulting oxidized dye. Together, these parameters provide a compact set of screening descriptors for evaluating light harvesting and the energetic feasibility of the principal charge-transfer steps.

We calculated these photovoltaic parameters using established theoretical models from the literature [19,20,22,23]. The specific equations and variables used to obtain these values are summarized in Table 2.

The excitation energy $E_{\mathrm{ex}}$ entering $\Delta G_{\mathrm{inj}}$ was taken as the vertical (Franck–Condon) S_0_→S_1_ TD-DFT excitation energy at the ground-state geometry, rather than the relaxed, adiabatic 0–0 transition energy, which would additionally require optimizing the S_1_ geometry of every conformer. Two considerations support this approximation. Electron injection into TiO_2_ has been shown to occur on an ultrafast, sub-100 fs timescale, faster than vibrational relaxation of the sensitizer excited state, making the vertical excitation energy a physically motivated approximation for the excitation energy relevant to this injection pathway [43]. Furthermore, a direct comparison of both schemes for triphenylamine-based D–π–A dyes found the resulting $\Delta G_{\mathrm{inj}}$ values to differ by a nearly constant offset of similar magnitude to the intrinsic accuracy of the underlying oxidation-potential estimates, so that the relative ranking of candidate sensitizers is preserved regardless of the scheme used [22]. Given this near-constant offset and the computational cost of optimizing the excited state for every conformer, we therefore adopted the vertical-excitation approximation consistently across the 150-dye screening library.

### 3.4. Selection of DSSC Sensitizers for Further Analysis

To rank the 150 D–π–A dyes according to a combined measure of charge-transfer character and optical response, we constructed a composite scoring index from five descriptors associated with charge-transfer character and light harvesting: the hole–electron overlap index $S_{r}$, the hole–electron centroid distance *D*, the charge-transfer separation index *t*, the S_0_→S_1_ transition wavelength $\lambda_{\max}$, and the oscillator strength *f*. Each descriptor was oriented so that larger values were uniformly favorable. $S_{r}$ was sign-inverted, since smaller values of this index indicate weaker hole–electron overlap and therefore stronger charge-transfer character; *D* was retained without modification; *t* was transformed to max(t,0), thereby assigning zero to nonpositive values; $\lambda_{\max}$ was transformed to $\max(\lambda_{\max}-300\;\mathrm{nm},0)$, so that only the portion above 300 nm contributed positively; and *f* was retained without modification. The 300 nm threshold was adopted as a pragmatic screening criterion to favor transitions extending into the near-UV/visible region rather than as a physical absorption cutoff.

The five oriented descriptors were then independently standardized to zero mean and unit variance across the 150-dye set with scikit-learn’s StandardScaler [44],

(1) $$z_{ij}=\frac{x_{ij}-{\bar{x}}_{j}}{\sigma_{j}},$$

where $x_{ij}$ is the oriented value of descriptor *j* for molecule *i*, and ${\bar{x}}_{j}$ and $\sigma_{j}$ are, respectively, the mean and standard deviation of descriptor *j* over the 150 dyes. Because all five standardized descriptors had equal weights, the composite score was expressed as their arithmetic mean,

(2) $${\mathrm{Score}}_{i}=\frac{1}{5}\sum_{j=1}^{5}z_{ij}.$$

The equal-weight scheme was adopted as a neutral screening criterion rather than as an assumption that the five descriptors contribute equally to experimental DSSC performance. Molecules were ranked in descending order of ${\mathrm{Score}}_{i}$, and the ten highest-scoring dyes (D5-B7-A3, D3-B7-A3, D4-B5-A2, D4-B1-A2, D5-B7-A2, D2-B1-A1, D3-B2-A1, D3-B1-A1, D4-B1-A1, and D3-B2-A3) were selected for the density-functional, kinetic, and dye–TiO_2_ interaction analyses discussed in the following sections. The full ranking of all 150 dyes, together with the underlying $S_{r}$, *D*, *t*, $\lambda_{\max}$, and *f* values, is reported in Table S1 of the Supporting Information.

### 3.5. Solvent-Phase Conformational Search and Excited-State Calculations for the Selected Sensitizers

For the ten top-ranked sensitizers, conformers were regenerated following the same CRENSO protocol described in Section 3.2 (GFN2-xTB), but now accounting for the polar solvent environment relevant to acetonitrile-based DSSC electrolytes. Conformational sampling and pre-optimization with CREST included the analytical linearized Poisson–Boltzmann (ALPB) implicit solvation model with acetonitrile as solvent. The resulting ensembles were refined at the r^2^SCAN-3c level [37] using CENSO [35] and ORCA 5 [38], now combined with the SMD continuum solvation model (acetonitrile) to account for bulk electrostatic solvent effects at the DFT level.

For each refined conformer of the ten selected dyes, we computed the first 12 singlet excited states by TD-DFT in Gaussian 16 [40], employing three exchange–correlation functionals spanning increasing long-range-corrected character, namely, B3LYP, CAM-B3LYP, and ωB97XD, all combined with the SMD(acetonitrile) solvation model using the 6-31G(d,p). This comparison allowed us to assess the sensitivity of the excited-state and photovoltaic descriptors to the amount and range dependence of Hartree–Fock exchange.

The photovoltaic parameters ($V_{OC}$, LHE, $\Delta G_{\mathrm{inj}}$, and $\Delta G_{\mathrm{reg}}$) and the descriptors used to characterize the nature of the S_0_→S_1_ transition ($S_{r}$, *D*, *t*, $\lambda_{\max}$, *f*) were obtained for each conformer following the procedure detailed in Section 3.3, using Multiwfn 3.8 [41,42] for the hole–electron density analysis. As in the gas-phase screening, conformer-level descriptors were Boltzmann-averaged at 298.15 K prior to computing the photovoltaic parameters, yielding one set of thermally corrected, solvent-phase values for each of the ten sensitizers at each of the three TD-DFT levels.

### 3.6. Reorganization Energy and Charge-Transfer Kinetics

To evaluate the charge-transfer kinetics within the adopted molecular model for the ten selected sensitizers, we computed the internal reorganization energy and the associated intramolecular charge-transfer rate constant for each conformer following a semiclassical Marcus-theory treatment [45,46]. The electron and hole reorganization energies, $\lambda_{e}$ and $\lambda_{h}$, were obtained by the four-point (adiabatic potential energy surfaces) method,

(3) $$\lambda_{e}=\left[E^{-}\left(M\right)-E^{-}\left(M^{-}\right)\right]+\left[E\left(M^{-}\right)-E\left(M\right)\right],$$

(4) $$\lambda_{h}=\left[E^{+}\left(M\right)-E^{+}\left(M^{+}\right)\right]+\left[E\left(M^{+}\right)-E\left(M\right)\right],$$

where E(M), $E^{-}\left(M^{-}\right)$, and $E^{+}\left(M^{+}\right)$ are the energies of the neutral, anionic, and cationic species, respectively, each computed at its own optimized geometry, while $E^{-}\left(M\right)$, $E(M^{-})$, $E^{+}\left(M\right)$, and $E(M^{+})$ denote single-point energies of the neutral or charged species evaluated at the optimized geometry of the opposite charge state. The total internal reorganization energy is then

(5) $$\lambda_{i}=\lambda_{h}+\lambda_{e}.$$

The electronic coupling between the two states selected to represent the relevant charge-transfer process, $V_{12}$, was estimated from the TD-DFT output via the generalized Mulliken–Hush formalism,

(6) $$V_{12}=\frac{\left|\mu_{12}\right|\cdot \Delta E_{12}}{\sqrt{{\left(\mu_{1}-\mu_{2}\right)}^{2}+4{\left(\mu_{12}\right)}^{2}}},$$

where $\mu_{1}$ and $\mu_{2}$ are the permanent dipole moments of the two adiabatic states, $\mu_{12}$ is their transition dipole moment, and $\Delta E_{12}$ is the vertical energy gap between these states. The two states used in the analysis were the adiabatic $S_{0}$ and $S_{1}$ TD-DFT states, taken as proxies for the donor-localized and acceptor-localized diabatic charge-transfer states within the standard two-state Mulliken–Hush approximation. Finally, the charge-transfer rate constant was estimated using the semiclassical Marcus expression under the zero-driving-force approximation, $\Delta G^{{}^{\circ}}=0$:

(7) $$k_{\mathrm{CT}}=\sqrt{\frac{\pi }{\lambda_{i}k_{B}T}}\cdot \frac{{\left|V_{12}\right|}^{2}}{\hbar }\cdot \exp\left(-\frac{\lambda_{i}}{4k_{B}T}\right),$$

where $k_{B}$ is the Boltzmann constant, T=298.15 K, and *ℏ* is the reduced Planck constant. All energies and dipole moments entering Equations (3)–(7) were obtained at the same TD-DFT level (B3LYP, CAM-B3LYP, or ωB97XD) and SMD(acetonitrile) solvation used for the excited-state calculations described above. As with the other excited-state descriptors, $\lambda_{i}$, $V_{12}$, and $k_{\mathrm{CT}}$ were evaluated per conformer and Boltzmann-averaged at 298.15 K, yielding ensemble-averaged values for each dye at each of the three functionals, reported in Tables S2–S7 of the Supporting Information.

The photovoltaic descriptors introduced below ($V_{OC}$, LHE, $\Delta G_{\mathrm{inj}}$, $\Delta G_{\mathrm{reg}}$, and $J_{\mathrm{SC}\text{-}\max}$), like $V_{12}$ and $k_{\mathrm{CT}}$ above, were evaluated per conformer and only then Boltzmann-averaged (average-of-the-property) throughout this work, since $\langle g\rangle =\sum_{i}p_{i}\;g\left({\mathrm{conformer}}_{i}\right)$ is the formally correct ensemble average of any observable *g*, as noted in Section 3.2. For $V_{OC}$, $\Delta G_{\mathrm{inj}}$, and $\Delta G_{\mathrm{reg}}$, which are linear in the underlying descriptors, this coincides exactly with the simpler property-of-the-average, g(〈X〉), computed once from the Boltzmann-averaged descriptors. For the nonlinear LHE, $J_{\mathrm{SC}-\max}$, $V_{12}$, and $k_{\mathrm{CT}}$, we assessed the extent to which evaluating the property at the Boltzmann-averaged structural parameters reproduces the corresponding ensemble-averaged property (Table S8 of the Supporting Information). For LHE, $J_{\mathrm{SC}\text{-}\max}$, and $V_{12}$, the two procedures showed relatively small differences, averaging only a few percent. In contrast, the approximation was substantially less reliable for $k_{\mathrm{CT}}$, for which differences of several orders of magnitude were obtained for dyes having a low-oscillator-strength dominant conformer. This greater sensitivity is consistent with the quadratic dependence of $k_{\mathrm{CT}}$ on $V_{12}$ and its exponential dependence on $\lambda_{i}$, which can amplify comparatively modest conformational variations in these quantities.

As an additional optical screening descriptor, we estimated an idealized short-circuit photocurrent density, $J_{\mathrm{SC}\text{-}\max}$, following Estrella et al. [47] and the implementation of Consiglio et al. [21]:

(8) $$J_{\mathrm{SC}\text{-}\max}=q\int \mathrm{LHE}\left(\lambda \right)\cdot \Phi_{\mathrm{inj}}\cdot \eta_{\mathrm{coll}}\cdot \vartheta_{\mathrm{ph},\mathrm{AM}1.5G}\left(\lambda \right)\;d\lambda ,$$

where *q* is the elementary charge and $\vartheta_{\mathrm{ph},\mathrm{AM}1.5G}\left(\lambda \right)$ is the photon flux of the AM1.5G reference solar spectrum [48]. Under the ideal-case assumption $\Phi_{\mathrm{inj}}=\eta_{\mathrm{coll}}=1$, Equation (8) was evaluated by numerically integrating $\mathrm{LHE}\left(\lambda \right)\cdot \vartheta_{\mathrm{ph},\mathrm{AM}1.5G}\left(\lambda \right)$ over 280–830 nm, with $\mathrm{LHE}\left(\lambda \right)=1-10^{-\varepsilon (\lambda )\Gamma }$ (Table 2) and ε(λ) a Gaussian-broadened molar absorption coefficient (half-width at half-maximum 0.35 eV) built from the S_0_→S_1_ oscillator strength and $\lambda_{\max}$ of each dye, and a fixed dye loading Γ=30 nmol cm^−2^, matching the parameters adopted by Consiglio et al. [21] Because only the S_0_→S_1_ transition contributes to LHE in this work (consistent with Table 2), rather than the full multi-state absorption spectrum used by Consiglio et al. [21], $J_{\mathrm{SC}-\max}$ should therefore be interpreted as a single-band analog of the idealized photocurrent descriptor rather than as a quantitative prediction of device photocurrent. $J_{\mathrm{SC}\text{-}\max}$ was calculated separately for each conformer using its corresponding *f* and $\lambda_{\max}$, and the resulting values were subsequently Boltzmann-averaged (average-of-the-property), as described above. For comparison, $J_{\mathrm{SC}\text{-}\max}$ was also evaluated from the Boltzmann-averaged *f* and $\lambda_{\max}$ (property-of-the-average). The results of both approaches, together with the corresponding LHE values, are reported for all ten sensitizers and three functionals in Table S8 of the Supporting Information.

### 3.7. Dye–TiO_2_ Anatase Interaction Energy

To evaluate the strength of the dye–semiconductor binding, we modeled the anchoring of the ten selected sensitizers onto the TiO_2_ anatase surface, following the computational strategy of Consiglio et al. [21]. A ${\left({\mathrm{TiO}}_{2}\right)}_{14}$ cluster, cleaved from the anatase (1 0 1) crystallographic surface, was used as a representative, finite model of the semiconductor. This cluster size was chosen in light of the systematic convergence studies in the literature [49]. Sánchez-de-Armas et al. [49] benchmarked a series of finite ${\left({\mathrm{TiO}}_{2}\right)}_{n}$ clusters (n=1–38) against each other and showed that the frontier-orbital and optical-absorption features of adsorbed dyes are already semiquantitatively converged by n≈6–9 Ti units. ${\left({\mathrm{TiO}}_{2}\right)}_{9}$ clusters have since been adopted as an electronically converged yet tractable semiconductor model in several TD-DFT studies of dye-sensitized electron injection [34,50]. The ${\left({\mathrm{TiO}}_{2}\right)}_{14}$ cluster used here exceeds these previously reported cluster sizes while providing a tractable surface model with a well-defined termination suitable for bidentate dye anchoring, consistent with Consiglio et al. [21]. Each dye was anchored to the cluster through its acceptor group in a bidentate binding mode, with the two oxygen atoms of the anchoring group bonded to two surface titanium atoms, consistent with the most stable binding geometry reported for cyanoacrylic-acid-type anchors. For the dyes bearing the carboxylic-acid-derived acceptors (A1, A2), the anchoring group was modeled in its fully dissociated bidentate form; to preserve the electroneutrality of the model, the detached proton was placed on a two-fold-coordinated (bridging) oxygen atom of the cluster, far from the anchoring site, following the approach of Sánchez-de-Armas et al. [34], consistent with the protonation scheme adopted by Consiglio et al. [21] for dyes anchoring through a carboxylic-acid-derived group. The resulting dye–${\left({\mathrm{TiO}}_{2}\right)}_{14}$ complexes were optimized at the B3LYP/def2-SVP level including dispersion corrections (GD3 model) with implicit solvation via the SMD model (acetonitrile), as implemented in Gaussian 16 [40]. Every dye–${\left({\mathrm{TiO}}_{2}\right)}_{14}$ complex was treated as a closed-shell singlet with an overall neutral charge, as expected for a stoichiometric, electroneutral anatase cluster. No pseudo-hydrogen capping of undercoordinated surface Ti atoms was introduced. Electroneutrality was preserved instead through the Ti:O stoichiometry of the cluster and, for the dissociated A1/A2 anchors, through the acidic-proton relocation described above.

The interaction energy between each dye and the TiO_2_ cluster was computed as

(9) $$E_{\mathrm{int}}=E_{{\mathrm{dye}@\mathrm{TiO}}_{2}}-\left(E_{\mathrm{dye}}+E_{{\mathrm{TiO}}_{2}}\right),$$

where $E_{{\mathrm{dye}@\mathrm{TiO}}_{2}}$ is the energy of the optimized complex, and $E_{\mathrm{dye}}$ and $E_{{\mathrm{TiO}}_{2}}$ are the energies of the isolated dye and bare cluster fragments, respectively, computed as single points at the same B3LYP/def2-SVP/SMD(acetonitrile) level using the geometry that each fragment adopts within the optimized complex, i.e., without further relaxation of the monomers. Basis-set superposition error (BSSE) was corrected using the standard counterpoise method of Boys and Bernardi, and both the raw and BSSE-corrected interaction energies are reported in Table 1.

## 4. Conclusions

This work introduces a thermally aware computational framework for screening D–π–A sensitizers that explicitly accounts for thermally accessible conformers when evaluating their electronic and photophysical properties, an aspect often overlooked in conventional screening approaches. By integrating conformational searches with excited-state TD-DFT calculations, we screened 150 donor–π–acceptor combinations and selected the ten highest-scoring sensitizers for further study, including solvation effects, charge-transfer kinetics, and explicit anchoring onto a TiO_2_ cluster model. This additional analysis showed that, despite exhibiting favorable electronic descriptors, some of the top-ranked dyes, particularly those bearing the A3 acceptor, exhibit substantially weaker dye–TiO_2_ interaction energies within the adopted cluster model, underscoring that promising charge-transfer character alone does not guarantee efficient anchoring.

Critically, comparing the Boltzmann-averaged descriptors of the ten top-ranked sensitizers against the values obtained from each dye’s single most-populated conformer revealed substantial divergence for a subset of the series. At the CAM-B3LYP level, $J_{\mathrm{SC}-\max}$ deviated by 18.4% on average and by up to 99.9% for D4-B1-A1, while $k_{\mathrm{CT}}$ ranged from −99.95% to +6.3% across the ten sensitizers, again dominated by D4-B1-A1. This sensitivity of $k_{\mathrm{CT}}$ originates primarily from the electronic coupling $V_{12}$ (mean absolute deviation of 13.0%, reaching −91.9% for D4-B1-A1) rather than from the internal reorganization energy $\lambda_{i}$ (mean absolute deviation of only 0.8%), identifying $V_{12}$ as the dominant source of the conformational sensitivity of the charge-transfer kinetics within the present model. This sensitivity is not a generic feature of the sensitizer library. It is instead confined to dyes whose most stable conformer places the donor and/or acceptor group nearly orthogonal to the conjugated bridge, giving an optically dark $S_{0}\to S_{1}$ transition, as found for D4-B1-A1 and, at the B3LYP level, for D4-B5-A2. Consequently, the relative ranking of sensitizers depends on whether conformational averaging is included, particularly for architectures prone to this dark-conformer effect, for which a single low-energy structure may not adequately capture the properties of the conformational ensemble.

These results indicate that conformational averaging is not uniformly critical across dipolar organic dyes, but becomes essential whenever the lowest-energy conformer decouples the donor and acceptor electronically, since a single-conformer treatment can then severely underestimate a sensitizer’s charge-transfer performance. The present framework therefore provides a practical route for flagging such cases and for prioritizing DSSC sensitizers whose favorable optical and charge-transfer properties persist across their thermally accessible conformational ensemble, rather than only at a single idealized geometry. 

## Figures and Tables

**Figure 1:**
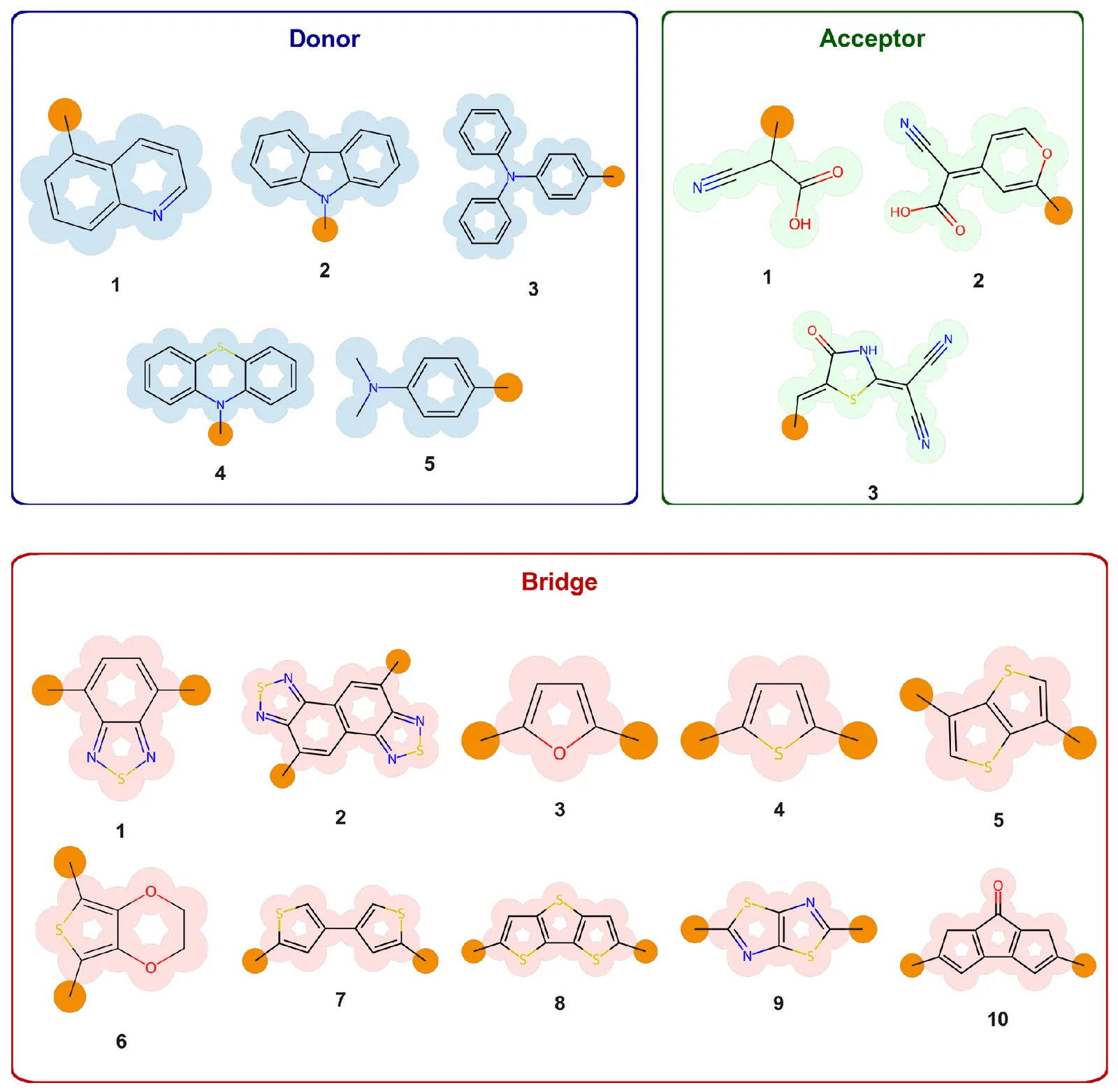
Donor (D1–D5), π-bridge (B1–B10), and acceptor (A1–A3) building blocks used to assemble the 150-dye D-π-A library. Orange circles indicate the bond formed between each fragment and its neighbor in the assembled dye.

**Figure 2:**
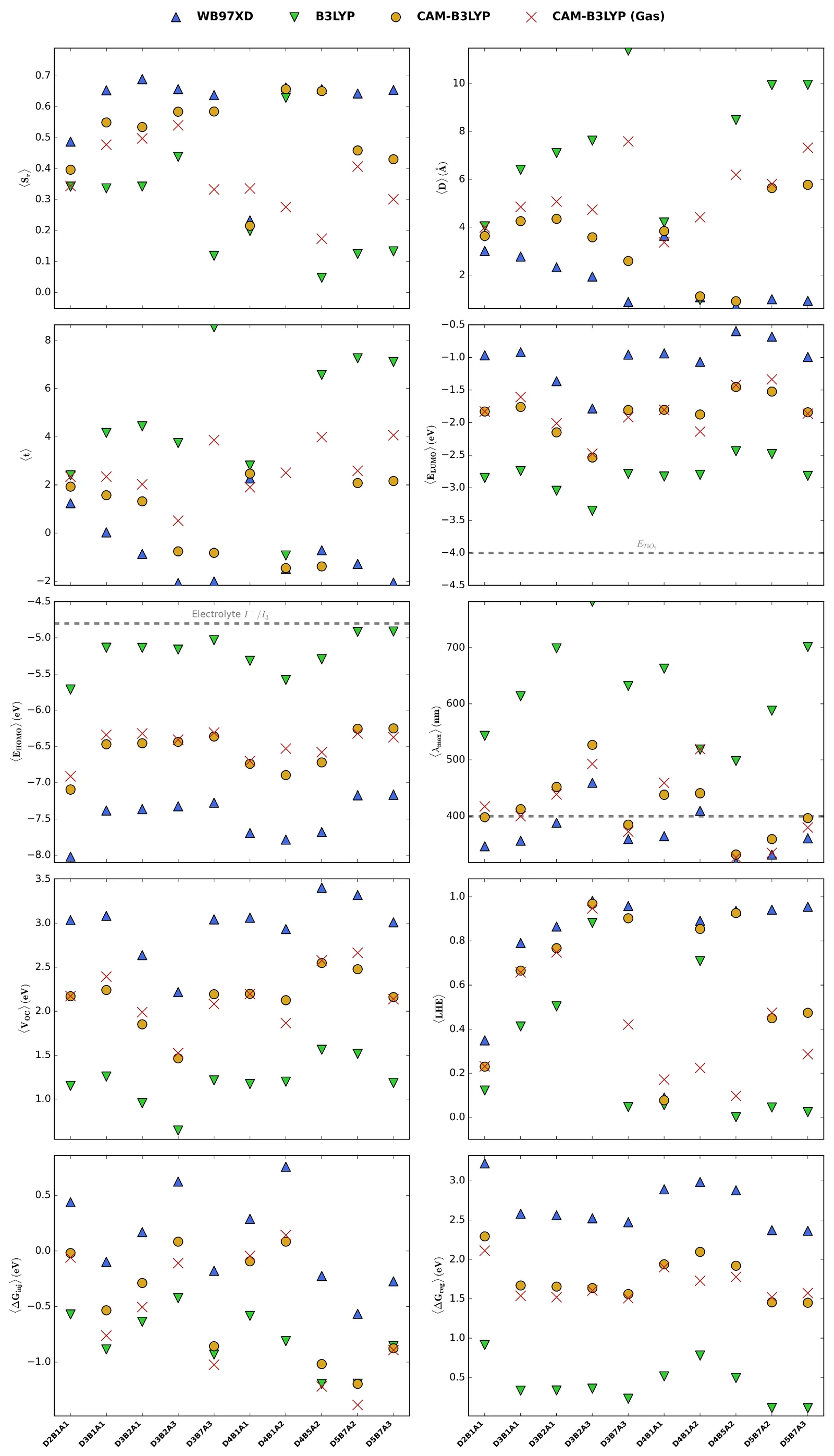
Comparison of excited-state, electronic, and photovoltaic descriptors for the ten top-ranked sensitizers calculated using ωB97XD, B3LYP, and CAM-B3LYP with implicit acetonitrile solvation, together with CAM-B3LYP gas-phase results. Only average values over the conformer ensembles are shown. Horizontal dashed lines represent the ${\mathrm{TiO}}_{2}$ conduction band edge ($E_{{\mathrm{TiO}}_{2}}\approx -4.0$ eV in $\langle E_{\mathrm{LUMO}}\rangle$), the electrolyte redox potential ($\mathrm{Electrolyte}\;I^{-}/I_{3}^{-}\approx -4.8$ eV in $\langle E_{\mathrm{HOMO}}\rangle$), and the threshold between the UV and visible light regions (400 nm in $\langle \lambda_{\max}\rangle$).

**Figure 3:**
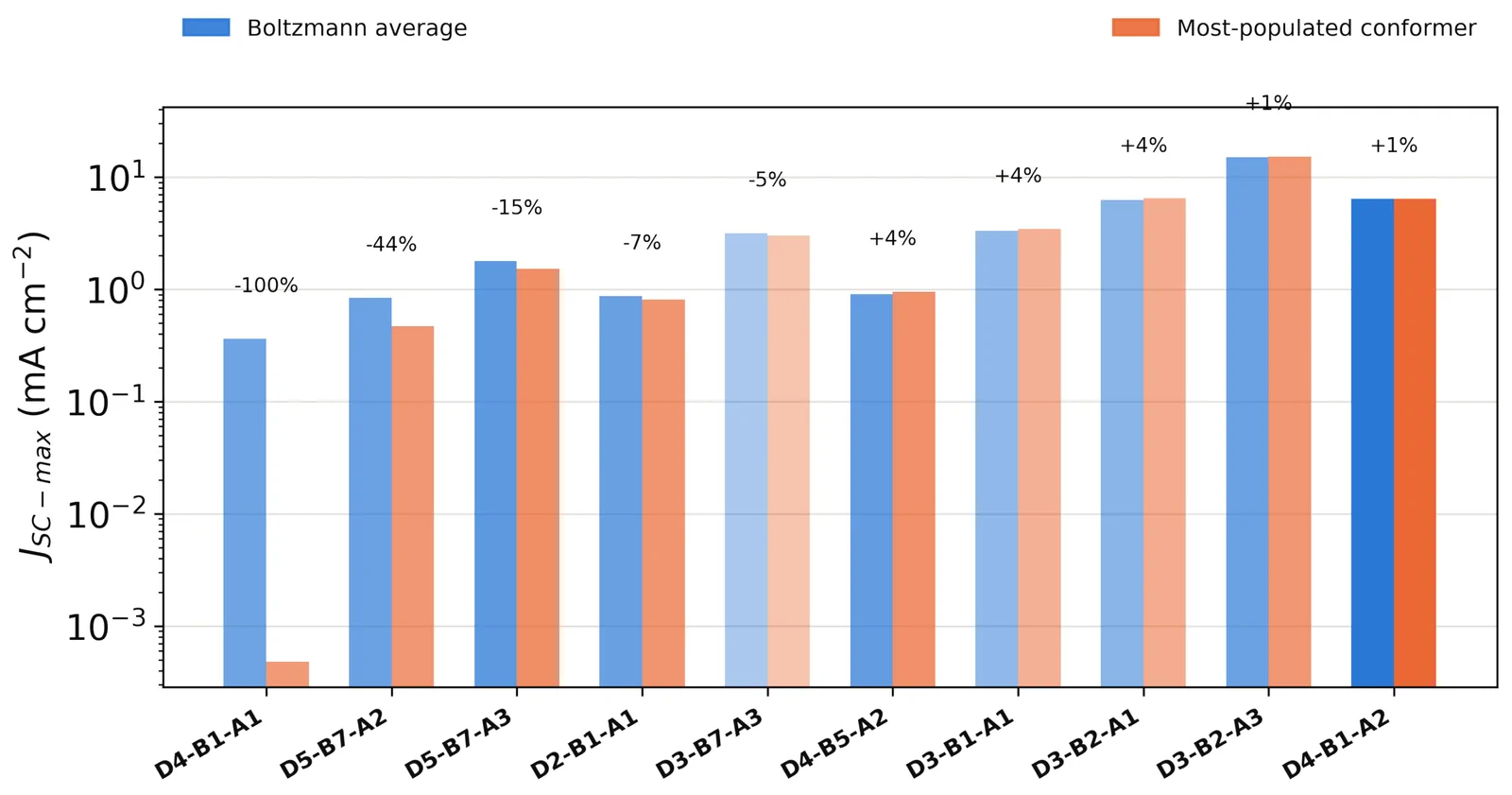
Comparison between Boltzmann-averaged and most-populated-conformer $J_{\mathrm{SC}-\max}$ for the ten top-ranked sensitizers at the CAM-B3LYP/6-31G(d,p)/SMD(acetonitrile) level. The percentage above each pair of bars represents the relative deviation of the most-populated-conformer value from the Boltzmann average, $\Delta J_{\mathrm{SC}-\max}=\left(J_{\mathrm{SC}-\max}^{\mathrm{best}}-\langle J_{\mathrm{SC}-\max}\rangle \right)/\langle J_{\mathrm{SC}-\max}\rangle$. Bar opacity is proportional to the Boltzmann weight of the most-populated conformer (darker = a single conformer dominates the ensemble; lighter = the population is spread across several conformers).

**Figure 4:**
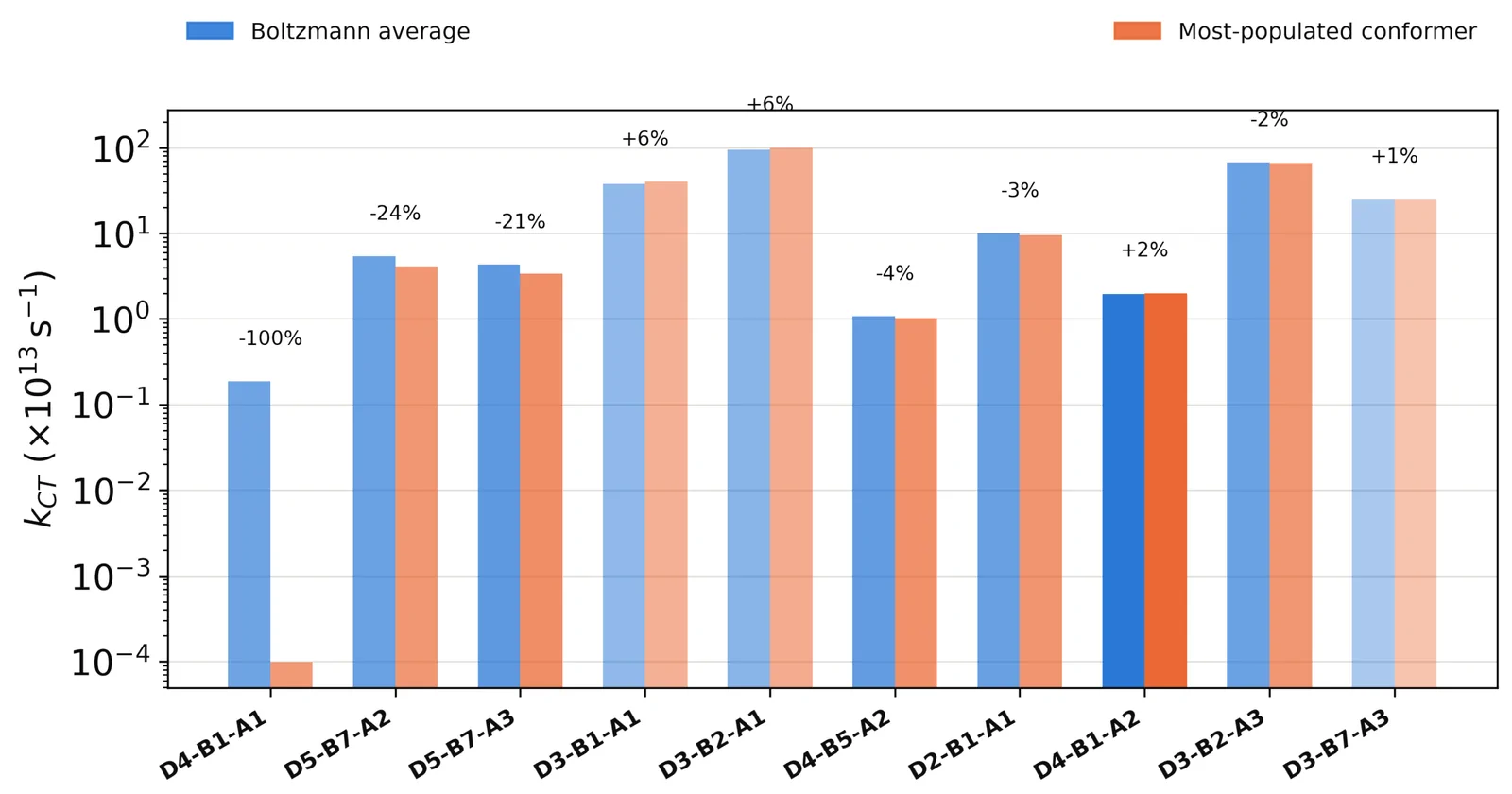
Comparison between Boltzmann-averaged and most-populated-conformer charge-transfer rate constants, $k_{\mathrm{CT}}$, for the ten top-ranked sensitizers at the CAM-B3LYP/6-31G(d,p)/SMD(acetonitrile) level. The percentage above each pair of bars represents the relative deviation of the most-populated-conformer value from the Boltzmann average, $\Delta k_{\mathrm{CT}}=\left(k_{\mathrm{CT}}^{\mathrm{best}}-\langle k_{\mathrm{CT}}\rangle \right)/\langle k_{\mathrm{CT}}\rangle$. Bar opacity is proportional to the Boltzmann weight of the most-populated conformer (darker = a single conformer dominates the ensemble; lighter = the population is spread across several conformers).

**Figure 5:**
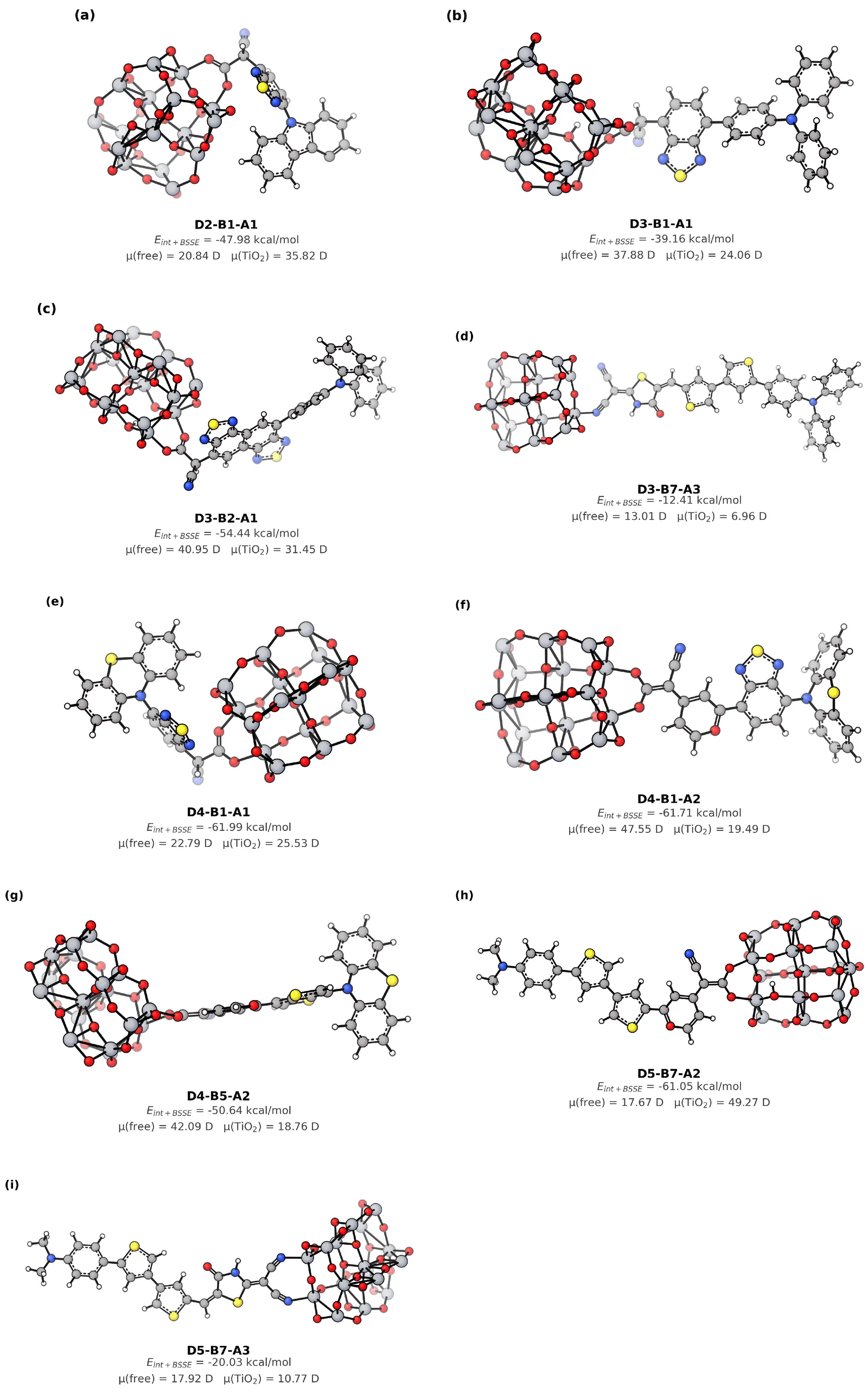
B3LYP/def2-SVP/SMD(acetonitrile) optimized structures of the dye–TiO_2_ complexes, for nine of the ten top-ranked sensitizers (D3-B2-A3 excluded; see the note below Table 1). For each complex, the BSSE-corrected interaction energy ($E_{\mathrm{int}+\mathrm{BSSE}}$, in kcal mol^−1^) and the dipole moment of the anchored (TiO_2_-bound, $\mu_{{\mathrm{TiO}}_{2}}$) and free (isolated dye, $\mu_{\mathrm{free}}$) forms (in Debye) are given below each structure.

**Table 1:** Interaction energies (raw and BSSE-corrected) of nine of the ten top-ranked dyes anchored onto the ${\left({\mathrm{TiO}}_{2}\right)}_{14}$ cluster, together with the dipole moments of the free dye and the anchored complex *.

Molecule	$E_{\mathrm{int}}$ (kcal/mol)	$E_{\mathrm{int}}$ + BSSE (kcal/mol)	Free Dye μ	Dye+(TiO_2_)_14_ μ
D2-B1-A1	−64.30	−47.98	20.84	35.82
D3-B1-A1	−52.53	−39.16	37.88	24.06
D3-B2-A1	−69.81	−54.44	40.95	31.45
D3-B7-A3	−17.71	−12.41	13.01	6.96
D4-B1-A1	−80.19	−61.99	22.79	25.53
D4-B1-A2	−75.28	−61.71	47.55	19.49
D4-B5-A2	−63.80	−50.64	42.09	18.76
D5-B7-A2	−74.71	−61.05	17.67	49.27
D5-B7-A3	−27.14	−20.03	17.92	10.77

* Note: D3-B2-A3 is excluded from this table and from Figure 5 because the anchoring-complex geometry optimization did not converge at the B3LYP/def2-SVP/SMD(acetonitrile) level of theory.

**Table 2:** Summary of the calculated photovoltaic parameters and their corresponding equations.

Parameter	Symbol	Equation
Open-circuit voltage	$V_{OC}$	$V_{OC}=E_{\mathrm{LUMO}}-E_{\mathrm{CB}}$
Light-harvesting efficiency	LHE	$\mathrm{LHE}=1-10^{-f}$
Electron injection driving force	$\Delta G_{\mathrm{inj}}$	$\Delta G_{\mathrm{inj}}=E_{\mathrm{dye}}^{*}-E_{\mathrm{CB}}$
Excited-state oxidation potential	$E_{\mathrm{dye}}^{*}$	$E_{\mathrm{dye}}^{*}=E_{\mathrm{dye}}^{0}-E_{\mathrm{ex}}$
Dye regeneration driving force	$\Delta G_{\mathrm{reg}}$	$\Delta G_{\mathrm{reg}}=E_{\mathrm{redox}}-E_{\mathrm{dye}}$

Note: $E_{\mathrm{CB}}$ is the conduction band redox potential, *f* is the oscillator strength at $\lambda_{\max}$, $E_{\mathrm{ex}}$ is the excitation energy, and $E_{\mathrm{redox}}$ is the redox potential of the mediator (−4.80 eV for the $I^{-}/I_{3}^{-}$ pair).

## Data Availability

The original contributions presented in this study are included in the article/Supplementary Materials. Further inquiries can be directed to the corresponding authors.

## Supplementary Materials

The following supporting information can be downloaded at https://www.mdpi.com/article/10.3390/molecules31193422/s1: Table S1: Composite *Z*-score ranking of the 150-dye screening library, with the underlying $S_{r}$, *D*, *t*, $\lambda_{\max}$, and *f* descriptors, gas-phase CAM-B3LYP/6-31G(d,p); Table S2: Hole–electron overlap, spatial charge-transfer, and frontier-orbital/photophysical parameters (per conformer and Boltzmann average), ten top-ranked sensitizers, CAM-B3LYP/SMD(acetonitrile); Table S3: Redox potentials, reorganization energies, dipole moments, electronic coupling $V_{12}$, and charge-transfer rate constant $k_{\mathrm{CT}}$ (per conformer and Boltzmann average), ten top-ranked sensitizers, CAM-B3LYP/SMD(acetonitrile); Table S4: As Table S2, B3LYP/SMD(acetonitrile); Table S5: As Table S3, B3LYP/SMD(acetonitrile); Table S6: As Table S2, ωB97XD/SMD(acetonitrile); Table S7: As Table S3, ωB97XD/SMD(acetonitrile); Table S8: Property-of-the-average versus average-of-the-property comparison for LHE, $J_{\mathrm{SC}\text{-}\max}$, $V_{12}$, and $k_{\mathrm{CT}}$, ten top-ranked sensitizers, three functionals; Figures S1 and S2: *D* index, Boltzmann average versus most-populated conformer, full 150-dye library; [App app1-molecules-31-03422]Figures S3 and S4: $S_{r}$ index, Boltzmann average versus most-populated conformer, full 150-dye library; Figures S5 and S6: *t* index, Boltzmann average versus most-populated conformer, full 150-dye library; Figures S7 and S8: $\lambda_{\max}$, Boltzmann average versus most-populated conformer, full 150-dye library; Figures S9 and S10: HOMO energy, Boltzmann average versus most-populated conformer, full 150-dye library; Figures S11 and S12: LUMO energy, Boltzmann average versus most-populated conformer, full 150-dye library; Figures S13 and S14: LHE, Boltzmann average versus most-populated conformer, full 150-dye library; Figures S15 and S16: $V_{OC}$, Boltzmann average versus most-populated conformer, full 150-dye library; Figures S17 and S18: $\Delta G_{\mathrm{inj}}$, Boltzmann average versus most-populated conformer, full 150-dye library; Figures S19 and S20: $\Delta G_{\mathrm{reg}}$, Boltzmann average versus most-populated conformer, full 150-dye library; Figures S21 and S22: $J_{\mathrm{SC}\text{-}\max}$, Boltzmann average versus most-populated conformer, full 150-dye library, gas phase; Figure S23: Simulated UV-Vis absorption spectra, most-populated conformer versus Boltzmann-weighted ensemble average, D2-B1-A1, D3-B1-A1, D3-B2-A1, D3-B2-A3, and D3-B7-A3, at B3LYP, CAM-B3LYP, and ωB97XD/SMD(acetonitrile); Figure S24: As Figure S23, D4-B1-A1, D4-B1-A2, D4-B5-A2, D5-B7-A2, and D5-B7-A3; Figure S25: Internal reorganization energy $\lambda_{i}$, Boltzmann average versus most-populated conformer, ten top-ranked sensitizers, CAM-B3LYP/SMD(acetonitrile); Figure S26: Electronic coupling $V_{12}$, Boltzmann average versus most-populated conformer, ten top-ranked sensitizers, CAM-B3LYP/SMD(acetonitrile); Figure S27: $J_{\mathrm{SC}\text{-}\max}$, Boltzmann average versus most-populated conformer, ten top-ranked sensitizers, B3LYP/SMD(acetonitrile); Figure S28: $J_{\mathrm{SC}\text{-}\max}$, Boltzmann average versus most-populated conformer, ten top-ranked sensitizers, ωB97XD/SMD(acetonitrile); Figure S29: Charge-transfer rate constant $k_{\mathrm{CT}}$, Boltzmann average versus most-populated conformer, ten top-ranked sensitizers, B3LYP/SMD(acetonitrile); Figure S30: Charge-transfer rate constant $k_{\mathrm{CT}}$, Boltzmann average versus most-populated conformer, ten top-ranked sensitizers, ωB97XD/SMD(acetonitrile); Data S1: Cartesian coordinates (XYZ format) of every optimized conformer and every TiO_2_-anchored structure reported in this work along with raw CSV data files underlying the descriptor and kinetic analyses reported in this work.
