# Supplementary material for: Accounting for Thermal Effects in the Charge-Transfer Properties of Dipolar Organic Dyes for Dye-Sensitized Solar Cells
Source: Molecules. 2026 Sep 26;31(19):3422. doi: 10.3390/molecules31193422 (PMC13666422; doi:10.3390/molecules31193422)
Supplement: Supplementary file 1 [file molecules-31-03422-s001.zip › molecules-4557397-supplementary tables and figures.pdf]

Supplementary Information

for

Accounting for Thermal Effect in Charge Transfer Properties of Dipolar Organic Dyes for Dye-Sensitized Solar Cells

Pierre C. Souza-Junior[1,‡], Mateus R. Barbosa[1,‡], Guilherme D. R. Matos[1], Flávio O. Sanches-Neto[2,3], João B. L. Martins[4,‡,*], and Daniel F. Scalabrini-Machado[1,‡,*]

[1]Laboratório de Modelagem de Sistemas Complexos, Instituto de Química, Universidade de Brasília-DF, 70910-900, Brazil
[2]Instituto Federal de Educação, Ciência e Tecnologia de Goiás, Goiânia-GO, 72876-601, GO, Brazil
[3]Grupo de Química Teórica e Estrutural de Anápolis, Centro de Pesquisa e Pós-Graduação, Universidade Estadual de Goiás, Anápolis-GO, 75132-40, Brazil
[4]Laboratório de Química Computacional, Instituto de Química, Universidade de Brasilia, Brasília-DF 70910-900, Brazil

* Correspondence: lopes@unb.br and daniel.scalabrini@unb.br
‡ These authors contributed equally to this work.

1 Composite Screening of the 150-Dye Library

1.1 Composite Z-Score Ranking of the 150-Dye Screening Library

Table S1 lists all 150 D–π–A dyes of the initial screening library, ranked in descending order of the composite score Score_i defined in the "Selection of DSSC Sensitizers for Further Analysis" subsection of the main text, together with the five Boltzmann-averaged, gas-phase (CAM-B3LYP/6-31G(d,p)) descriptors from which it was computed: the hole–electron overlap index S_r, the hole–electron centroid distance D, the charge-transfer separation index t, the $S_0 \rightarrow S_1$ transition wavelength λ_{max}, and the oscillator strength f. The ten top-ranked sensitizers selected for further analysis in the main text are shaded.

Table S1: Composite Z-score ranking of the 150-dye screening library, together with the five underlying descriptors (Boltzmann-averaged, gas-phase CAM-B3LYP/6-31G(d,p)). Shaded rows are the ten top-ranked sensitizers carried forward for solvent-phase, multi-functional, kinetic, and anchoring analysis.

**Rank**	**Molecule**	S_r	D (Å)	t (Å)	λ_{max} (nm)	f	**Score**
1	D5_B7_A3	0.301	7.324	4.069	379.8	0.1550	2.011
2	D3_B7_A3	0.333	7.587	3.865	372.3	0.2587	1.945
3	D4_B5_A2	0.174	6.195	3.993	326.4	0.0591	1.888
4	D4_B1_A2	0.276	4.420	2.510	519.2	0.1107	1.605
5	D5_B7_A2	0.407	5.809	2.589	334.8	0.3000	1.107
6	D2_B1_A1	0.344	3.999	2.344	417.3	0.1140	1.077
7	D3_B2_A1	0.498	5.074	2.029	438.9	0.6000	1.067
8	D3_B1_A1	0.477	4.854	2.350	400.1	0.4664	1.007
9	D4_B1_A1	0.336	3.371	1.909	459.0	0.0944	0.998
10	D3_B2_A3	0.540	4.736	0.520	492.8	1.2921	0.903
11	D3_B2_A2	0.543	4.758	0.863	474.7	1.0793	0.874
12	D3_B1_A3	0.536	4.246	0.173	477.4	1.3929	0.734
13	D5_B1_A3	0.535	3.944	0.353	465.0	1.4140	0.710
14	D5_B8_A3	0.602	3.500	-1.052	455.5	2.3900	0.699
15	D5_B5_A2	0.446	4.842	1.666	323.9	0.4820	0.669
16	D4_B6_A2	0.396	3.807	1.131	363.1	0.6244	0.629
17	D5_B1_A2	0.517	3.918	0.556	436.3	1.0030	0.586
18	D3_B9_A3	0.628	3.236	-1.435	453.8	2.2976	0.577
19	D5_B2_A3	0.583	3.552	-0.629	494.5	1.4230	0.554
20	D4_B2_A1	0.421	3.001	0.845	461.4	0.2369	0.548
21	D5_B9_A3	0.601	3.036	-1.318	441.2	2.2410	0.545
22	D5_B2_A2	0.569	3.914	0.460	464.6	1.0110	0.543
23	D4_B4_A2	0.398	3.418	0.925	368.2	0.6635	0.540
24	D3_B1_A2	0.557	4.059	0.504	438.4	1.0353	0.531
25	D2_B2_A1	0.482	3.441	1.095	453.0	0.2040	0.526
26	D5_B5_A3	0.628	3.010	-1.392	439.3	2.3220	0.510
27	D3_B5_A3	0.636	2.821	-1.843	442.9	2.4028	0.502

Continued on next page

Table S1 – Continued from previous page

**Rank**	**Molecule**	S_r	D (Å)	t (Å)	$\lambda_{\max}$ (nm)	f	**Score**
28	D3_B8_A3	0.646	2.917	-1.822	450.5	2.3498	0.502
29	D2_B1_A3	0.499	3.268	0.399	484.3	0.5950	0.494
30	D5_B1_A1	0.529	3.760	1.486	397.0	0.3860	0.484
31	D5_B2_A1	0.556	3.766	0.895	435.8	0.5540	0.439
32	D4_B8_A3	0.587	3.000	-0.949	420.5	1.8123	0.375
33	D3_B6_A3	0.597	2.882	-1.101	440.4	1.7398	0.373
34	D3_B3_A3	0.619	2.567	-1.471	424.7	2.0821	0.347
35	D3_B8_A2	0.654	2.771	-2.167	413.7	2.2932	0.342
36	D5_B6_A2	0.588	3.395	-0.347	391.8	1.7880	0.341
37	D5_B6_A3	0.605	3.049	-0.618	431.0	1.6850	0.337
38	D2_B2_A3	0.544	2.843	-0.799	500.5	0.7160	0.324
39	D3_B9_A2	0.658	3.217	-1.293	412.8	2.0477	0.321
40	D3_B4_A3	0.620	2.774	-1.232	432.9	1.7844	0.306
41	D5_B8_A2	0.647	2.842	-1.651	410.4	2.1000	0.297
42	D5_B3_A3	0.603	2.427	-1.288	414.1	1.9700	0.292
43	D2_B4_A1	0.429	3.347	1.453	277.2	0.2140	0.276
44	D4_B5_A3	0.596	2.389	-1.228	410.6	1.9214	0.275
45	D5_B4_A2	0.600	3.270	-0.352	388.0	1.7300	0.271
46	D4_B2_A2	0.487	2.417	-0.393	478.4	0.5949	0.270
47	D5_B4_A3	0.616	2.447	-1.427	422.3	1.8370	0.252
48	D2_B8_A3	0.608	2.380	-1.662	414.2	1.8950	0.251
49	D2_B1_A2	0.482	3.034	-0.037	455.9	0.3990	0.244
50	D3_B10_A3	0.608	1.428	-2.390	552.7	1.0393	0.244
51	D4_B9_A3	0.602	2.394	-1.234	407.2	1.8749	0.238
52	D5_B10_A3	0.595	1.119	-1.923	563.6	0.9380	0.225
53	D1_B8_A3	0.663	2.165	-2.147	412.2	2.1893	0.200
54	D5_B3_A2	0.575	3.169	-0.050	379.2	1.4540	0.194
55	D2_B9_A3	0.637	2.145	-1.840	411.5	1.9870	0.182
56	D3_B7_A2	0.542	4.389	0.483	332.4	0.6482	0.178
57	D2_B2_A2	0.550	2.679	-0.539	479.1	0.5180	0.164

Continued on next page

Table S1 – Continued from previous page

Rank	Molecule	S_r	D (Å)	t (Å)	$\lambda_{\max}$ (nm)	f	Score
58	D3_B4_A2	0.644	2.993	-0.958	392.9	1.7022	0.151
59	D3_B5_A2	0.551	4.297	0.451	324.2	0.7615	0.150
60	D4_B4_A1	0.394	2.986	1.123	304.4	0.0021	0.148
61	D2_B3_A3	0.582	2.651	-0.702	410.7	1.3020	0.147
62	D4_B8_A1	0.452	3.223	0.716	317.6	0.4470	0.140
63	D3_B10_A2	0.621	1.736	-1.877	539.5	0.7592	0.138
64	D5_B9_A2	0.677	2.522	-1.609	402.4	1.9670	0.128
65	D3_B6_A2	0.653	2.630	-1.350	398.8	1.7639	0.116
66	D5_B10_A2	0.625	1.477	-1.817	552.8	0.6830	0.108
67	D2_B9_A2	0.624	2.637	-1.046	376.6	1.6590	0.076
68	D3_B3_A2	0.627	3.040	-0.483	386.8	1.3317	0.058
69	D1_B9_A3	0.693	1.983	-2.276	414.3	1.9659	0.052
70	D2_B5_A3	0.623	1.987	-1.954	420.3	1.4680	0.049
71	D4_B8_A2	0.622	2.131	-1.556	377.2	1.6415	0.003
72	D2_B4_A3	0.592	2.175	-1.308	416.8	1.0550	-0.001
73	D4_B3_A2	0.543	2.654	-0.068	367.0	0.9726	-0.005
74	D4_B9_A2	0.625	2.179	-1.384	373.7	1.5774	-0.025
75	D4_B2_A3	0.642	1.092	-2.455	466.3	1.2443	-0.055
76	D4_B3_A3	0.631	1.445	-1.742	390.2	1.7115	-0.055
77	D3_B10_A1	0.622	1.899	-0.831	497.5	0.3182	-0.096
78	D4_B5_A1	0.455	2.730	0.525	307.1	0.1947	-0.098
79	D1_B5_A3	0.683	1.569	-2.527	414.6	1.5862	-0.107
80	D4_B7_A3	0.550	2.187	-1.088	352.6	1.0300	-0.110
81	D4_B6_A3	0.621	1.072	-2.241	403.9	1.4684	-0.126
82	D4_B4_A3	0.628	1.247	-2.009	393.4	1.5138	-0.131
83	D1_B1_A1	0.577	2.788	0.870	341.3	0.1884	-0.136
84	D5_B5_A1	0.629	3.252	-0.038	307.2	1.3450	-0.139
85	D4_B10_A3	0.626	0.752	-1.450	511.8	0.6039	-0.143
86	D1_B8_A2	0.698	1.410	-2.909	378.7	1.9602	-0.146
87	D2_B3_A2	0.598	2.460	-0.380	365.6	0.9550	-0.150

Continued on next page

Table S1 – Continued from previous page

Rank	Molecule	S_r	D (Å)	t (Å)	$\lambda_{\max}$ (nm)	f	**Score**
88	D1_B10_A3	0.657	0.678	-2.464	528.2	0.6044	-0.165
89	D1_B2_A3	0.650	1.143	-2.779	420.5	1.3289	-0.167
90	D3_B9_A1	0.667	2.616	-0.832	352.3	1.3710	-0.168
91	D2_B10_A3	0.662	0.528	-0.942	543.8	0.5370	-0.173
92	D2_B10_A2	0.637	0.745	-1.579	529.1	0.3480	-0.194
93	D1_B3_A3	0.688	1.126	-2.446	381.5	1.8167	-0.203
94	D5_B10_A1	0.636	1.315	-1.286	503.0	0.2570	-0.211
95	D4_B10_A2	0.619	0.640	-1.161	501.2	0.4005	-0.239
96	D3_B6_A1	0.636	2.876	-0.271	321.6	1.1055	-0.241
97	D3_B3_A1	0.619	2.835	-0.147	309.5	1.0774	-0.257
98	D4_B1_A3	0.657	0.691	-1.880	428.2	1.1874	-0.270
99	D3_B7_A1	0.620	2.845	0.000	309.8	1.0214	-0.276
100	D1_B7_A3	0.588	1.314	-1.876	353.6	1.1312	-0.277
101	D3_B5_A1	0.680	2.284	-1.407	328.2	1.4221	-0.294
102	D5_B8_A1	0.696	2.266	-1.385	329.2	1.5230	-0.294
103	D1_B1_A3	0.650	0.917	-2.350	397.2	1.2284	-0.299
104	D3_B8_A1	0.708	1.738	-2.315	345.8	1.6732	-0.299
105	D1_B10_A2	0.664	0.450	-1.643	519.1	0.3993	-0.301
106	D5_B9_A1	0.678	2.358	-0.893	327.7	1.3270	-0.310
107	D2_B7_A3	0.600	1.146	-1.963	348.1	1.1970	-0.320
108	D4_B10_A1	0.616	0.908	-0.748	479.3	0.1437	-0.337
109	D1_B4_A3	0.688	1.106	-2.539	393.1	1.2385	-0.352
110	D2_B8_A1	0.583	2.732	-0.261	298.7	0.6240	-0.371
111	D3_B4_A1	0.664	2.520	-0.620	314.9	1.0551	-0.383
112	D1_B2_A1	0.627	2.388	-0.265	365.6	0.4049	-0.386
113	D1_B6_A3	0.692	0.512	-2.999	381.3	1.5384	-0.387
114	D2_B9_A1	0.602	2.519	-0.137	304.9	0.7130	-0.398
115	D1_B1_A2	0.614	1.370	-1.219	370.2	0.6940	-0.407
116	D2_B5_A1	0.594	2.763	0.120	296.2	0.4460	-0.412
117	D2_B10_A1	0.630	0.857	-1.214	461.9	0.1500	-0.418

Continued on next page

Table S1 – Continued from previous page

**Rank**	**Molecule**	S_r	D (Å)	t (Å)	$\lambda_{\max}$ (nm)	f	**Score**
118	D1_B2_A2	0.661	1.071	-2.044	397.1	0.8424	-0.419
119	D1_B9_A2	0.737	0.499	-1.617	375.0	1.6976	-0.445
120	D5_B4_A1	0.666	2.512	-0.281	287.0	0.9980	-0.448
121	D1_B10_A1	0.662	0.469	-1.229	487.2	0.1420	-0.466
122	D5_B6_A1	0.679	2.329	-0.453	293.6	1.0360	-0.487
123	D2_B8_A2	0.716	0.650	-1.104	375.7	1.2830	-0.509
124	D1_B6_A2	0.694	0.482	-1.440	349.1	1.4493	-0.517
125	D5_B7_A1	0.674	2.383	-0.231	277.4	0.8640	-0.523
126	D2_B6_A3	0.669	0.308	-2.289	375.6	1.0850	-0.530
127	D5_B3_A1	0.696	1.841	-0.957	274.2	1.1680	-0.550
128	D1_B3_A2	0.718	0.933	-1.901	369.9	1.0017	-0.575
129	D1_B4_A2	0.727	0.892	-2.277	361.1	1.1509	-0.577
130	D4_B6_A1	0.529	1.841	-0.099	297.8	0.0031	-0.591
131	D4_B7_A2	0.670	1.158	-1.162	322.0	0.9466	-0.606
132	D1_B7_A2	0.674	0.896	-1.018	322.6	1.0412	-0.619
133	D2_B7_A2	0.668	0.905	-1.121	322.7	1.0030	-0.619
134	D4_B7_A1	0.542	1.409	-0.306	296.9	0.0005	-0.679
135	D2_B4_A2	0.726	1.312	-0.726	339.3	0.7620	-0.698
136	D2_B6_A2	0.714	0.582	-1.157	348.9	0.9160	-0.707
137	D1_B8_A1	0.751	1.186	-2.272	311.5	1.1315	-0.730
138	D4_B9_A1	0.654	1.058	-1.673	290.8	0.6747	-0.737
139	D1_B5_A2	0.726	0.781	-1.390	328.2	0.8021	-0.795
140	D2_B5_A2	0.746	0.755	-1.934	331.8	0.7960	-0.828
141	D1_B6_A1	0.711	1.351	-0.946	282.5	0.4096	-0.886
142	D1_B9_A1	0.761	0.580	-2.398	301.6	0.8532	-0.952
143	D1_B5_A1	0.780	1.093	-1.931	304.1	0.6905	-0.958
144	D4_B3_A1	0.621	0.492	-0.980	282.9	0.0014	-0.964
145	D1_B7_A1	0.762	1.361	-0.729	275.5	0.3830	-0.990
146	D1_B3_A1	0.773	1.245	-0.831	280.8	0.4722	-1.001
147	D2_B3_A1	0.723	1.048	-0.852	275.8	0.0900	-1.053

Continued on next page

Table S1 – Continued from previous page

**Rank**	**Molecule**	S_r	D (Å)	t (Å)	$\lambda_{\max}$ (nm)	f	**Score**
148	D1_B4_A1	0.797	0.729	-1.573	286.5	0.4006	-1.145
149	D2_B7_A1	0.759	0.737	-0.942	271.2	0.0740	-1.173
150	D2_B6_A1	0.784	0.328	-1.075	273.4	0.0850	-1.278

2 Conformer-Resolved Electronic, Photophysical, and Charge-Transfer Kinetics Data for the Top-10 Sensitizers

2.1 Boltzmann-Weighted Ensemble Averaging

For a molecule comprising n conformers, the statistical population p_i of the i-th conformer is obtained from its relative Gibbs free energy ($\Delta G_i = G_i - G_{\min}$) via Maxwell–Boltzmann statistics,

$$p_i = \frac{\exp\left(-\dfrac{\Delta G_i}{k_B T}\right)}{\sum_{j=1}^{n} \exp\left(-\dfrac{\Delta G_j}{k_B T}\right)}, \tag{S1}$$

with $k_B = 8.617333 \times 10^{-5}$ eV K^{-1} and $T = 298.15$ K. Every ensemble-averaged property $\langle X \rangle$ reported in the main text and in the tables below is then the population-weighted expectation value

$$\langle X \rangle = \sum_{i=1}^{n} p_i \, X_i, \tag{S2}$$

evaluated directly from the per-conformer values X_i (not from a formula applied to already-averaged inputs), with the exception of V_{OC}, ΔG_{inj}, and ΔG_{reg}, which are instead computed once from the Boltzmann-averaged underlying descriptors (mathematically equivalent for these linear quantities), as detailed in the main text (Section 2.6). Section 2.5 below additionally verifies, for the nonlinear LHE, $J_{\text{SC-max}}$, V_{12}, and k_{CT}, how much the property-of-the-average alternative would have differed from the average-of-the-property values reported here.

2.2 CAM-B3LYP/6-31G(d,p)

Table S2: Hole–electron overlap, spatial charge-transfer, and frontier-orbital/photophysical parameters for the conformational ensemble of the ten selected sensitizers at CAM-B3LYP/6-31G(d,p)/SMD(acetonitrile). The Boltzmann-weighted ensemble average is shown in the highlighted row beneath each conformer set, labeled ⟨Molecule⟩.

**Molecule / Conf.**	**Pop. (%)**	S_r	D (Å)	t (Å)	**HOMO (eV)**	**LUMO (eV)**	**Gap (eV)**	$\lambda_{\max}$ (nm)	f
D2_B1_A1-CONF1	59.96	0.389	3.604	1.924	-7.09	-1.81	5.28	396.7	0.1083
D2_B1_A1-CONF2	22.32	0.410	3.674	1.934	-7.10	-1.85	5.25	400.3	0.1217
D2_B1_A1-CONF3	17.73	0.404	3.716	1.975	-7.10	-1.86	5.24	400.7	0.1220
**⟨D2_B1_A1⟩**	–	**0.396**	**3.639**	**1.935**	**-7.09**	**-1.83**	**5.26**	**398.2**	**0.1137**
D3_B1_A1-CONF1	1.68	0.557	4.171	1.518	-6.46	-1.70	4.75	409.0	0.4803
D3_B1_A1-CONF2	0.63	0.555	4.184	1.502	-6.46	-1.75	4.71	413.3	0.5042
D3_B1_A1-CONF3	1.19	0.551	4.253	1.622	-6.47	-1.71	4.76	406.2	0.4426
D3_B1_A1-CONF4	0.54	0.549	4.328	1.667	-6.47	-1.75	4.72	409.8	0.4591
D3_B1_A1-CONF5	40.26	0.555	4.227	1.534	-6.46	-1.76	4.70	413.4	0.4979
D3_B1_A1-CONF6	22.73	0.548	4.332	1.651	-6.48	-1.76	4.72	411.2	0.4551
D3_B1_A1-CONF7	32.97	0.544	4.249	1.571	-6.47	-1.76	4.71	413.4	0.4649
**⟨D3_B1_A1⟩**	–	**0.550**	**4.258**	**1.574**	**-6.47**	**-1.76**	**4.71**	**412.7**	**0.4762**
D3_B2_A1-CONF1	0.20	0.541	4.287	1.229	-6.45	-2.15	4.30	453.7	0.6640
D3_B2_A1-CONF2	0.81	0.540	4.291	1.288	-6.45	-2.11	4.34	447.5	0.6203
D3_B2_A1-CONF3	0.08	0.536	4.248	1.228	-6.46	-2.15	4.31	454.8	0.6488
D3_B2_A1-CONF4	0.57	0.531	4.359	1.350	-6.46	-2.11	4.35	444.9	0.5924
D3_B2_A1-CONF5	4.00	0.537	4.275	1.240	-6.45	-2.13	4.32	450.8	0.6356
D3_B2_A1-CONF6	30.10	0.527	4.424	1.414	-6.46	-2.14	4.32	448.7	0.6063
D3_B2_A1-CONF7	43.86	0.540	4.317	1.276	-6.45	-2.16	4.29	453.6	0.6556
D3_B2_A1-CONF8	20.39	0.532	4.331	1.311	-6.46	-2.15	4.31	452.4	0.6261
**⟨D3_B2_A1⟩**	–	**0.534**	**4.350**	**1.324**	**-6.46**	**-2.15**	**4.31**	**451.7**	**0.6333**
D3_B2_A3-CONF1	58.74	0.587	3.598	-0.751	-6.43	-2.54	3.89	528.1	1.5182
D3_B2_A3-CONF2	41.26	0.581	3.562	-0.763	-6.45	-2.53	3.91	525.0	1.4810
**⟨D3_B2_A3⟩**	–	**0.584**	**3.583**	**-0.756**	**-6.44**	**-2.54**	**3.90**	**526.8**	**1.5028**
D3_B7_A3-CONF1	16.52	0.585	2.330	-1.160	-6.36	-1.79	4.58	381.3	1.0741
D3_B7_A3-CONF2	27.51	0.585	2.414	-1.053	-6.37	-1.79	4.58	382.0	1.0437
D3_B7_A3-CONF3	13.58	0.598	2.746	-0.740	-6.36	-1.79	4.57	386.8	1.1250

Continued on next page

Table S2 – Continued from previous page

Molecule / Conf.	Pop. (%)	S_r	D (Å)	t (Å)	HOMO (eV)	LUMO (eV)	Gap (eV)	$\lambda_{\max}$ (nm)	f
D3_B7_A3-CONF4	5.94	0.607	2.644	-0.815	-6.36	-1.78	4.57	385.1	1.1256
D3_B7_A3-CONF5	12.28	0.593	2.642	-0.786	-6.36	-1.79	4.58	385.5	1.1248
D3_B7_A3-CONF6	5.61	0.571	2.930	-0.091	-6.36	-1.87	4.49	391.5	0.8076
D3_B7_A3-CONF7	5.18	0.573	3.059	-0.021	-6.35	-1.87	4.48	394.6	0.7750
D3_B7_A3-CONF8	9.42	0.564	2.610	-0.904	-6.37	-1.87	4.50	386.1	0.9436
D3_B7_A3-CONF9	3.96	0.568	3.045	-0.020	-6.35	-1.86	4.49	392.7	0.7926
**⟨D3_B7_A3⟩**	–	**0.585**	**2.593**	**-0.819**	**-6.36**	**-1.81**	**4.56**	**385.1**	**1.0281**
D4_B1_A1-CONF1	0.64	0.636	1.918	0.230	-6.97	-1.58	5.39	407.6	0.2633
D4_B1_A1-CONF2	0.71	0.627	1.958	0.425	-6.95	-1.55	5.41	406.5	0.2500
D4_B1_A1-CONF3	0.30	0.630	1.930	0.236	-6.97	-1.58	5.38	408.2	0.2654
D4_B1_A1-CONF4	0.46	0.634	1.912	0.210	-6.97	-1.58	5.39	407.4	0.2665
D4_B1_A1-CONF5	56.43	0.131	4.237	2.938	-6.69	-1.84	4.85	442.8	0.0000
D4_B1_A1-CONF6	26.08	0.131	4.242	2.935	-6.69	-1.87	4.83	446.9	0.0000
D4_B1_A1-CONF7	15.37	0.614	1.971	0.285	-6.97	-1.60	5.37	409.9	0.2550
**⟨D4_B1_A1⟩**	–	**0.216**	**3.841**	**2.474**	**-6.74**	**-1.80**	**4.94**	**438.1**	**0.0447**
D4_B1_A2-CONF1	91.03	0.657	1.108	-1.486	-6.90	-1.88	5.02	441.3	0.8406
D4_B1_A2-CONF2	4.63	0.660	1.330	-0.751	-6.90	-1.78	5.12	429.1	0.7675
D4_B1_A2-CONF3	4.34	0.658	1.147	-1.429	-6.90	-1.88	5.02	440.9	0.8371
**⟨D4_B1_A2⟩**	–	**0.657**	**1.120**	**-1.449**	**-6.90**	**-1.88**	**5.02**	**440.7**	**0.8370**
D4_B5_A2-CONF2	16.66	0.663	0.997	-0.773	-6.71	-1.52	5.19	325.8	1.1609
D4_B5_A2-CONF3	62.85	0.651	0.857	-1.529	-6.72	-1.44	5.28	333.1	1.1405
D4_B5_A2-CONF4	0.29	0.526	3.151	0.262	-6.76	-1.40	5.36	351.2	0.7393
D4_B5_A2-CONF5	3.35	0.647	1.090	-0.942	-6.75	-1.49	5.26	325.2	1.0881
D4_B5_A2-CONF6	9.00	0.635	0.996	-1.601	-6.74	-1.43	5.32	333.8	1.0897
D4_B5_A2-CONF7	3.03	0.652	0.890	-1.543	-6.72	-1.45	5.27	332.6	1.1615
D4_B5_A2-CONF9	0.16	0.653	1.181	-0.979	-6.74	-1.49	5.25	324.4	1.1029
D4_B5_A2-CONF10	4.66	0.649	0.959	-1.481	-6.74	-1.43	5.30	332.9	1.1228
**⟨D4_B5_A2⟩**	–	**0.651**	**0.914**	**-1.382**	**-6.72**	**-1.45**	**5.27**	**331.7**	**1.1362**
D5_B7_A2-CONF1	3.68	0.545	4.447	1.260	-6.33	-1.52	4.81	360.0	0.4453
D5_B7_A2-CONF2	58.83	0.416	6.229	2.683	-6.24	-1.52	4.73	357.0	0.1460

Continued on next page

Table S2 – Continued from previous page

**Molecule / Conf.**	**Pop. (%)**	S_r	D (Å)	t (Å)	**HOMO (eV)**	**LUMO (eV)**	**Gap (eV)**	λ_{max} (nm)	f
D5_B7_A2-CONF3	7.16	0.499	4.315	0.864	-6.35	-1.52	4.83	352.2	0.4554
D5_B7_A2-CONF4	2.81	0.495	4.121	0.583	-6.35	-1.53	4.82	354.6	0.4466
D5_B7_A2-CONF5	27.33	0.526	5.039	1.388	-6.24	-1.53	4.70	366.5	0.5281
D5_B7_A2-CONF6	0.18	0.544	4.455	1.314	-6.33	-1.53	4.80	360.1	0.4409
**⟨D5_B7_A2⟩**	–	**0.459**	**5.639**	**2.085**	**-6.26**	**-1.52**	**4.73**	**359.3**	**0.2926**
D5_B7_A3-CONF1	10.07	0.483	4.915	1.089	-6.25	-1.78	4.47	388.8	0.4153
D5_B7_A3-CONF2	15.27	0.481	5.115	1.305	-6.24	-1.78	4.46	390.0	0.3646
D5_B7_A3-CONF3	5.56	0.490	4.835	1.237	-6.23	-1.78	4.45	396.5	0.6904
D5_B7_A3-CONF4	3.62	0.463	4.481	1.476	-6.32	-1.86	4.46	401.9	0.5391
D5_B7_A3-CONF5	65.48	0.404	6.206	2.650	-6.25	-1.87	4.38	399.2	0.2150
**⟨D5_B7_A3⟩**	–	**0.430**	**5.771**	**2.166**	**-6.25**	**-1.84**	**4.41**	**396.7**	**0.2962**

Table S3: Redox potentials, reorganization energies, dipole moments, electronic coupling (V_{12}), and Marcus charge-transfer rate constant (k_{ct}) for CAM-B3LYP/6-31G(d,p)/SMD(acetonitrile). The Boltzmann-weighted ensemble average is shown in the highlighted row beneath each conformer set, labeled ⟨Molecule⟩.

**Molecule / Conf.**	**IP (eV)**	**EA (eV)**	λ_e (eV)	λ_h (eV)	λ_i (eV)	μ_1 (a.u.)	μ_2 (a.u.)	μ_{12} (a.u.)	V_{12} (eV)	k_{ct} (10^{13} s^{-1})
D2_B1_A1-CONF1	5.76	3.13	0.366	0.103	0.469	0.636	6.594	1.270	0.6127	9.6036
D2_B1_A1-CONF2	5.77	3.16	0.343	0.112	0.455	0.982	7.811	1.394	0.5852	10.2017
D2_B1_A1-CONF3	5.77	3.17	0.340	0.102	0.442	0.987	8.136	1.390	0.5606	10.7400
**⟨D2_B1_A1⟩**	**5.77**	**3.14**	**0.356**	**0.105**	**0.461**	**0.775**	**7.139**	**1.319**	**0.5973**	**9.9386**
D3_B1_A1-CONF1	5.15	3.01	0.314	0.112	0.426	1.238	9.441	2.803	0.8552	29.7863
D3_B1_A1-CONF2	5.16	3.05	0.296	0.114	0.410	1.970	10.503	2.826	0.8282	33.3211
D3_B1_A1-CONF3	5.15	3.02	0.326	0.111	0.437	1.213	9.466	2.671	0.8292	24.8133
D3_B1_A1-CONF4	5.16	3.05	0.298	0.111	0.409	1.947	10.675	2.720	0.8001	31.5510
D3_B1_A1-CONF5	5.16	3.06	0.294	0.109	0.403	3.831	11.727	2.837	0.8749	40.0310
D3_B1_A1-CONF6	5.16	3.06	0.295	0.110	0.405	3.780	11.747	2.736	0.8536	37.3751
D3_B1_A1-CONF7	5.16	3.06	0.296	0.110	0.407	3.727	11.718	2.723	0.8446	35.9930
**⟨D3_B1_A1⟩**	**5.16**	**3.06**	**0.296**	**0.110**	**0.406**	**3.688**	**11.650**	**2.773**	**0.8585**	**37.6547**

Continued on next page

Table S3 – Continued from previous page

**Molecule / Conf.**	**IP (eV)**	**EA (eV)**	λ_e (eV)	λ_h (eV)	λ_i (eV)	μ_1 (a.u.)	μ_2 (a.u.)	μ_{12} (a.u.)	V_{12} (eV)	k_{ct} (10^{13} s^{-1})
D3_B2_A1-CONF1	5.16	3.28	0.193	0.108	0.301	2.188	11.773	3.104	0.7426	90.1326
D3_B2_A1-CONF2	5.15	3.25	0.201	0.107	0.308	1.945	11.087	3.004	0.7608	87.0611
D3_B2_A1-CONF3	5.16	3.28	0.201	0.109	0.309	2.265	11.748	3.046	0.7367	80.7930
D3_B2_A1-CONF4	5.16	3.25	0.206	0.108	0.315	1.925	11.167	2.922	0.7446	77.7035
D3_B2_A1-CONF5	5.16	3.27	0.192	0.108	0.300	2.887	12.385	3.029	0.7395	90.2008
D3_B2_A1-CONF6	5.16	3.28	0.193	0.108	0.301	1.888	11.346	2.922	0.7262	86.0513
D3_B2_A1-CONF7	5.16	3.29	0.191	0.107	0.298	3.483	12.548	3.084	0.7688	100.1384
D3_B2_A1-CONF8	5.16	3.29	0.192	0.108	0.300	3.492	12.491	2.997	0.7595	95.7213
**⟨D3_B2_A1⟩**	**5.16**	**3.29**	**0.192**	**0.108**	**0.300**	**2.956**	**12.146**	**3.014**	**0.7526**	**94.3313**
D3_B2_A3-CONF1	5.17	3.65	0.228	0.110	0.338	5.742	16.386	4.747	0.7814	65.8889
D3_B2_A3-CONF2	5.18	3.64	0.232	0.102	0.334	5.723	16.274	4.644	0.7803	68.5142
**⟨D3_B2_A3⟩**	**5.17**	**3.65**	**0.230**	**0.107**	**0.336**	**5.734**	**16.340**	**4.705**	**0.7810**	**66.9721**
D3_B7_A3-CONF1	5.11	3.09	0.313	0.158	0.471	5.032	13.814	3.360	0.9882	24.4380
D3_B7_A3-CONF2	5.11	3.08	0.311	0.158	0.469	5.007	14.006	3.447	0.9868	24.8926
D3_B7_A3-CONF3	5.10	3.08	0.308	0.164	0.472	4.799	14.168	3.572	0.9717	23.4105
D3_B7_A3-CONF4	5.10	3.08	0.307	0.158	0.465	4.686	14.356	3.568	0.9558	24.2695
D3_B7_A3-CONF5	5.10	3.08	0.312	0.161	0.472	4.805	13.844	3.582	0.9988	24.6072
D3_B7_A3-CONF6	5.10	3.17	0.317	0.158	0.475	4.296	10.891	3.214	1.1053	29.1888
D3_B7_A3-CONF7	5.10	3.17	0.319	0.159	0.478	4.341	11.255	3.143	1.0569	25.8558
D3_B7_A3-CONF8	5.11	3.17	0.319	0.160	0.480	5.041	13.348	3.340	1.0061	23.1035
D3_B7_A3-CONF9	5.10	3.17	0.319	0.156	0.476	4.323	11.336	3.189	1.0620	26.7559
**⟨D3_B7_A3⟩**	**5.10**	**3.11**	**0.313**	**0.159**	**0.472**	**4.841**	**13.512**	**3.424**	**0.9997**	**24.7403**
D4_B1_A1-CONF1	5.63	2.91	0.602	0.788	1.390	2.634	5.155	2.241	1.3256	0.0033
D4_B1_A1-CONF2	5.60	2.88	0.637	0.763	1.400	1.857	4.830	2.174	1.2588	0.0027
D4_B1_A1-CONF3	5.63	2.91	0.603	0.785	1.388	2.203	5.933	2.243	1.1677	0.0027
D4_B1_A1-CONF4	5.62	2.91	0.606	0.783	1.389	2.734	5.983	2.246	1.2328	0.0029
D4_B1_A1-CONF5	5.37	3.15	0.337	0.541	0.878	1.278	6.885	0.034	0.0169	0.0001
D4_B1_A1-CONF6	5.37	3.16	0.367	0.542	0.909	1.010	7.727	0.033	0.0135	0.0000
D4_B1_A1-CONF7	5.63	2.92	0.593	0.183	0.776	3.807	7.904	2.164	1.0982	1.2096
**⟨D4_B1_A1⟩**	**5.42**	**3.11**	**0.390**	**0.491**	**0.881**	**1.619**	**7.229**	**0.407**	**0.2084**	**0.1861**

Continued on next page

Table S3 – Continued from previous page

**Molecule / Conf.**	**IP (eV)**	**EA (eV)**	λ_e (eV)	λ_h (eV)	λ_i (eV)	μ_1 (a.u.)	μ_2 (a.u.)	μ_{12} (a.u.)	V_{12} (eV)	k_{ct} (10^{13} s^{-1})
D4_B1_A2-CONF1	5.63	3.11	0.624	0.141	0.765	5.951	8.368	3.637	1.3332	2.0024
D4_B1_A2-CONF2	5.62	3.03	0.674	0.153	0.826	3.974	5.375	3.374	1.4146	1.1896
D4_B1_A2-CONF3	5.63	3.11	0.626	0.149	0.775	7.032	9.439	3.668	1.3361	1.8090
**⟨D4_B1_A2⟩**	**5.63**	**3.11**	**0.626**	**0.142**	**0.768**	**5.906**	**8.275**	**3.626**	**1.3371**	**1.9563**
D4_B5_A2-CONF2	5.40	2.84	0.327	0.535	0.862	3.697	2.148	3.756	1.8634	1.4281
D4_B5_A2-CONF3	5.40	2.78	0.347	0.543	0.890	2.867	4.007	3.215	1.8325	1.0319
D4_B5_A2-CONF4	5.49	2.75	0.338	0.300	0.638	4.263	9.914	2.672	1.2128	6.2434
D4_B5_A2-CONF5	5.43	2.82	0.343	0.571	0.914	2.788	2.898	3.662	1.9061	0.8726
D4_B5_A2-CONF6	5.42	2.78	0.345	0.562	0.906	3.949	3.735	3.209	1.8563	0.8992
D4_B5_A2-CONF7	5.40	2.78	0.350	0.541	0.891	4.293	5.236	3.348	1.8455	1.0357
D4_B5_A2-CONF9	5.43	2.82	0.346	0.570	0.916	4.199	4.058	3.756	1.9104	0.8633
D4_B5_A2-CONF10	5.43	2.78	0.386	0.568	0.954	5.345	4.766	3.294	1.8549	0.5478
**⟨D4_B5_A2⟩**	**5.41**	**2.79**	**0.345**	**0.545**	**0.890**	**3.265**	**3.725**	**3.327**	**1.8420**	**1.0730**
D5_B7_A2-CONF1	4.99	2.83	0.323	0.274	0.597	2.476	10.995	2.236	0.8002	4.1775
D5_B7_A2-CONF2	4.90	2.83	0.328	0.169	0.497	5.068	17.638	1.749	0.4657	4.1230
D5_B7_A2-CONF3	5.00	2.83	0.328	0.276	0.604	4.940	14.339	2.472	0.8194	4.0688
D5_B7_A2-CONF4	5.00	2.85	0.328	0.273	0.601	5.053	15.451	2.152	0.6684	2.7936
D5_B7_A2-CONF5	4.90	2.85	0.325	0.171	0.496	5.839	16.654	2.396	0.6852	8.9609
D5_B7_A2-CONF6	4.99	2.84	0.327	0.275	0.602	3.388	10.251	2.232	0.9387	5.4246
**⟨D5_B7_A2⟩**	**4.91**	**2.84**	**0.327**	**0.184**	**0.511**	**5.171**	**16.814**	**2.008**	**0.5699**	**5.4084**
D5_B7_A3-CONF1	4.92	3.08	0.310	0.196	0.506	6.452	18.655	2.307	0.5639	5.4571
D5_B7_A3-CONF2	4.90	3.08	0.309	0.171	0.480	6.489	19.035	2.240	0.5346	6.5157
D5_B7_A3-CONF3	4.92	3.08	0.323	0.199	0.523	5.776	17.857	2.822	0.6618	6.2839
D5_B7_A3-CONF4	4.98	3.17	0.312	0.256	0.568	4.443	13.811	2.648	0.7591	5.0966
D5_B7_A3-CONF5	4.90	3.17	0.329	0.170	0.499	6.487	20.021	1.946	0.4291	3.4029
**⟨D5_B7_A3⟩**	**4.91**	**3.14**	**0.323**	**0.177**	**0.501**	**6.370**	**19.388**	**2.101**	**0.4837**	**4.3066**

2.3 B3LYP/6-31G(d,p)

Table S4: Hole–electron overlap, spatial charge-transfer, and frontier-orbital/photophysical parameters for the conformational ensemble of the ten selected sensitizers at B3LYP/6-31G(d,p)/SMD(acetonitrile). The Boltzmann-weighted ensemble average is shown in the highlighted row beneath each conformer set, labeled ⟨Molecule⟩.

**Molecule / Conf.**	**Pop. (%)**	S_r	D (Å)	t (Å)	**HOMO (eV)**	**LUMO (eV)**	**Gap (eV)**	λ_{max} (nm)	f
D2_B1_A1-CONF1	59.96	0.337	4.047	2.418	-5.71	-2.83	2.88	540.9	0.0532
D2_B1_A1-CONF2	22.32	0.352	4.015	2.361	-5.72	-2.86	2.85	544.9	0.0617
D2_B1_A1-CONF3	17.73	0.347	4.053	2.400	-5.72	-2.87	2.84	546.8	0.0622
**⟨D2_B1_A1⟩**	–	**0.342**	**4.041**	**2.402**	**-5.71**	**-2.85**	**2.87**	**542.8**	**0.0567**
D3_B1_A1-CONF1	1.68	0.347	6.295	4.058	-5.13	-2.69	2.43	601.0	0.2419
D3_B1_A1-CONF2	0.63	0.350	6.314	4.045	-5.13	-2.73	2.40	609.4	0.2586
D3_B1_A1-CONF3	1.19	0.321	6.468	4.306	-5.13	-2.70	2.43	605.9	0.1985
D3_B1_A1-CONF4	0.54	0.326	6.489	4.305	-5.13	-2.74	2.40	613.6	0.2106
D3_B1_A1-CONF5	40.26	0.346	6.373	4.112	-5.13	-2.74	2.39	611.7	0.2537
D3_B1_A1-CONF6	22.73	0.327	6.468	4.270	-5.14	-2.75	2.39	615.4	0.2100
D3_B1_A1-CONF7	32.97	0.330	6.386	4.157	-5.14	-2.75	2.39	615.4	0.2193
**⟨D3_B1_A1⟩**	–	**0.336**	**6.399**	**4.165**	**-5.14**	**-2.74**	**2.39**	**613.5**	**0.2313**
D3_B2_A1-CONF1	0.20	0.355	7.036	4.346	-5.13	-3.04	2.09	695.8	0.3344
D3_B2_A1-CONF2	0.81	0.348	7.027	4.400	-5.13	-3.01	2.12	688.1	0.3012
D3_B2_A1-CONF3	0.08	0.351	6.953	4.275	-5.14	-3.04	2.10	696.4	0.3184
D3_B2_A1-CONF4	0.57	0.326	7.188	4.595	-5.14	-3.02	2.12	692.3	0.2641
D3_B2_A1-CONF5	4.00	0.346	7.077	4.419	-5.13	-3.03	2.10	695.1	0.3089
D3_B2_A1-CONF6	30.10	0.329	7.169	4.543	-5.14	-3.04	2.10	698.7	0.2789
D3_B2_A1-CONF7	43.86	0.352	7.064	4.392	-5.13	-3.05	2.08	699.3	0.3257
D3_B2_A1-CONF8	20.39	0.340	7.055	4.389	-5.14	-3.05	2.09	699.6	0.2967
**⟨D3_B2_A1⟩**	–	**0.342**	**7.095**	**4.439**	**-5.14**	**-3.05**	**2.09**	**698.9**	**0.3045**
D3_B2_A3-CONF1	58.74	0.444	7.595	3.699	-5.16	-3.36	1.80	781.3	0.9626
D3_B2_A3-CONF2	41.26	0.430	7.658	3.808	-5.16	-3.35	1.81	783.1	0.8821
**⟨D3_B2_A3⟩**	–	**0.438**	**7.621**	**3.744**	**-5.16**	**-3.36**	**1.81**	**782.0**	**0.9294**
D3_B7_A3-CONF1	16.52	0.106	12.055	9.017	-5.03	-2.77	2.26	623.3	0.0062
D3_B7_A3-CONF2	27.51	0.110	12.154	9.119	-5.04	-2.77	2.27	622.1	0.0073
D3_B7_A3-CONF3	13.58	0.133	11.112	8.326	-5.03	-2.76	2.26	626.1	0.0465

Continued on next page

Table S4 – Continued from previous page

Molecule / Conf.	Pop. (%)	S_r	D (Å)	t (Å)	HOMO (eV)	LUMO (eV)	Gap (eV)	λ_{max} (nm)	f
D3_B7_A3-CONF4	5.94	0.125	11.126	8.381	-5.03	-2.76	2.26	626.2	0.0360
D3_B7_A3-CONF5	12.28	0.125	11.155	8.411	-5.03	-2.76	2.26	625.5	0.0370
D3_B7_A3-CONF6	5.61	0.126	9.551	7.313	-5.03	-2.84	2.18	660.5	0.0249
D3_B7_A3-CONF7	5.18	0.143	9.526	7.232	-5.02	-2.84	2.18	661.3	0.0364
D3_B7_A3-CONF8	9.42	0.108	11.644	8.694	-5.04	-2.85	2.19	650.4	0.0064
D3_B7_A3-CONF9	3.96	0.133	9.527	7.297	-5.02	-2.84	2.18	662.2	0.0302
**⟨D3_B7_A3⟩**	–	**0.119**	**11.378**	**8.552**	**-5.03**	**-2.79**	**2.25**	**631.9**	**0.0211**
D4_B1_A1-CONF1	0.64	0.594	2.236	0.472	-5.57	-2.58	2.99	492.4	0.1741
D4_B1_A1-CONF2	0.71	0.585	2.280	0.637	-5.56	-2.55	3.00	491.5	0.1657
D4_B1_A1-CONF3	0.30	0.587	2.276	0.498	-5.57	-2.59	2.99	494.4	0.1746
D4_B1_A1-CONF4	0.46	0.592	2.233	0.455	-5.58	-2.58	3.00	491.7	0.1776
D4_B1_A1-CONF5	56.43	0.120	4.598	3.287	-5.26	-2.87	2.39	694.4	0.0000
D4_B1_A1-CONF6	26.08	0.120	4.606	3.288	-5.27	-2.90	2.37	704.9	0.0000
D4_B1_A1-CONF7	15.37	0.569	2.329	0.548	-5.57	-2.60	2.97	499.3	0.1648
**⟨D4_B1_A1⟩**	–	**0.199**	**4.202**	**2.808**	**-5.32**	**-2.83**	**2.49**	**662.9**	**0.0290**
D4_B1_A2-CONF1	91.03	0.628	0.939	-0.955	-5.58	-2.80	2.78	519.0	0.5382
D4_B1_A2-CONF2	4.63	0.637	1.442	-0.408	-5.57	-2.73	2.85	506.0	0.4921
D4_B1_A2-CONF3	4.34	0.631	0.949	-0.914	-5.58	-2.80	2.78	518.6	0.5352
**⟨D4_B1_A2⟩**	–	**0.629**	**0.963**	**-0.928**	**-5.58**	**-2.80**	**2.78**	**518.4**	**0.5359**
D4_B5_A2-CONF2	16.66	0.046	8.420	6.524	-5.29	-2.50	2.78	512.4	0.0001
D4_B5_A2-CONF3	62.85	0.048	8.521	6.604	-5.29	-2.42	2.87	495.7	0.0001
D4_B5_A2-CONF4	0.29	0.268	6.241	3.797	-5.35	-2.38	2.97	488.8	0.0870
D4_B5_A2-CONF5	3.35	0.038	8.361	6.529	-5.31	-2.47	2.84	501.4	0.0001
D4_B5_A2-CONF6	9.00	0.050	8.413	6.548	-5.31	-2.42	2.90	491.1	0.0006
D4_B5_A2-CONF7	3.03	0.048	8.590	6.644	-5.29	-2.43	2.86	496.2	0.0001
D4_B5_A2-CONF9	0.16	0.040	8.360	6.520	-5.31	-2.47	2.84	502.2	0.0001
D4_B5_A2-CONF10	4.66	0.034	8.538	6.696	-5.31	-2.42	2.89	491.3	0.0001
**⟨D4_B5_A2⟩**	–	**0.048**	**8.485**	**6.580**	**-5.29**	**-2.44**	**2.86**	**498.0**	**0.0004**
D5_B7_A2-CONF1	3.68	0.143	8.811	6.659	-4.98	-2.48	2.49	575.8	0.0168
D5_B7_A2-CONF2	58.83	0.117	10.191	7.441	-4.91	-2.48	2.43	588.2	0.0157

Continued on next page

Table S4 – Continued from previous page

Molecule / Conf.	Pop. (%)	S_r	D (Å)	t (Å)	HOMO (eV)	LUMO (eV)	Gap (eV)	$\lambda_{\max}$ (nm)	f
D5_B7_A2-CONF3	7.16	0.117	10.249	7.493	-4.99	-2.48	2.52	564.4	0.0112
D5_B7_A2-CONF4	2.81	0.125	10.531	7.718	-5.00	-2.50	2.51	564.9	0.0091
D5_B7_A2-CONF5	27.33	0.141	9.410	6.886	-4.90	-2.50	2.40	597.8	0.0336
D5_B7_A2-CONF6	0.18	0.146	8.758	6.626	-4.98	-2.48	2.49	575.0	0.0177
**⟨D5_B7_A2⟩**	–	**0.125**	**9.938**	**7.271**	**-4.92**	**-2.48**	**2.43**	**588.0**	**0.0202**
D5_B7_A3-CONF1	10.07	0.128	10.442	7.523	-4.91	-2.76	2.15	675.0	0.0083
D5_B7_A3-CONF2	15.27	0.134	10.418	7.476	-4.91	-2.76	2.15	677.3	0.0102
D5_B7_A3-CONF3	5.56	0.151	9.640	7.019	-4.90	-2.76	2.14	682.9	0.0432
D5_B7_A3-CONF4	3.62	0.164	8.210	6.075	-4.97	-2.83	2.14	691.5	0.0355
D5_B7_A3-CONF5	65.48	0.130	9.891	7.036	-4.91	-2.84	2.07	713.1	0.0067
**⟨D5_B7_A3⟩**	–	**0.133**	**9.952**	**7.116**	**-4.91**	**-2.82**	**2.10**	**701.4**	**0.0105**

Table S5: Redox potentials, reorganization energies, dipole moments, electronic coupling (V_{12}), and Marcus charge-transfer rate constant (k_{ct}) for B3LYP/6-31G(d,p)/SMD(acetonitrile). The Boltzmann-weighted ensemble average is shown in the highlighted row beneath each conformer set, labeled ⟨Molecule⟩.

Molecule / Conf.	IP (eV)	EA (eV)	λ_e (eV)	λ_h (eV)	λ_i (eV)	μ_1 (a.u.)	μ_2 (a.u.)	μ_{12} (a.u.)	V_{12} (eV)	k_{ct} (10^{13} s^{-1})
D2_B1_A1-CONF1	5.48	2.96	0.335	0.094	0.429	0.659	7.214	1.218	0.3992	6.2802
D2_B1_A1-CONF2	5.48	2.99	0.314	0.104	0.418	0.991	8.070	1.318	0.3969	7.0387
D2_B1_A1-CONF3	5.49	3.00	0.308	0.093	0.402	0.996	8.343	1.319	0.3830	7.7923
**⟨D2_B1_A1⟩**	**5.48**	**2.97**	**0.326**	**0.096**	**0.422**	**0.793**	**7.605**	**1.258**	**0.3958**	**6.7176**
D3_B1_A1-CONF1	4.89	2.84	0.284	0.102	0.386	1.405	13.238	2.450	0.3946	9.8726
D3_B1_A1-CONF2	4.90	2.88	0.267	0.105	0.372	2.153	14.177	2.515	0.3926	11.3245
D3_B1_A1-CONF3	4.89	2.84	0.296	0.102	0.398	1.345	13.401	2.196	0.3502	6.7670
D3_B1_A1-CONF4	4.90	2.88	0.268	0.102	0.370	2.105	14.400	2.284	0.3519	9.3851
D3_B1_A1-CONF5	4.90	2.88	0.265	0.100	0.365	3.939	15.458	2.506	0.4044	13.0850
D3_B1_A1-CONF6	4.90	2.88	0.266	0.101	0.366	3.868	15.473	2.299	0.3711	10.8026
D3_B1_A1-CONF7	4.90	2.88	0.267	0.101	0.368	3.819	15.347	2.312	0.3750	10.8011
**⟨D3_B1_A1⟩**	**4.90**	**2.88**	**0.267**	**0.101**	**0.367**	**3.789**	**15.349**	**2.389**	**0.3860**	**11.6530**

Continued on next page

Table S5 – Continued from previous page

**Molecule / Conf.**	**IP (eV)**	**EA (eV)**	λ_e (eV)	λ_h (eV)	λ_i (eV)	μ_1 (a.u.)	μ_2 (a.u.)	μ_{12} (a.u.)	V_{12} (eV)	k_{ct} (10^{13} s^{-1})
D3_B2_A1-CONF1	4.90	3.19	0.165	0.098	0.264	2.474	15.922	3.028	0.3658	33.6555
D3_B2_A1-CONF2	4.89	3.16	0.173	0.098	0.271	2.131	15.202	2.884	0.3637	30.4702
D3_B2_A1-CONF3	4.90	3.19	0.173	0.099	0.272	2.551	15.919	2.945	0.3590	29.4357
D3_B2_A1-CONF4	4.90	3.17	0.178	0.099	0.277	2.077	15.360	2.690	0.3361	24.3160
D3_B2_A1-CONF5	4.90	3.18	0.165	0.098	0.263	3.126	16.576	2.900	0.3531	31.6486
D3_B2_A1-CONF6	4.90	3.19	0.165	0.099	0.264	2.099	15.606	2.757	0.3354	28.2850
D3_B2_A1-CONF7	4.90	3.20	0.163	0.097	0.261	3.707	16.828	2.989	0.3675	35.2198
D3_B2_A1-CONF8	4.90	3.20	0.165	0.099	0.264	3.699	16.791	2.854	0.3542	31.3286
**⟨D3_B2_A1⟩**	**4.90**	**3.19**	**0.165**	**0.098**	**0.263**	**3.173**	**16.418**	**2.886**	**0.3543**	**32.0881**
D3_B2_A3-CONF1	4.92	3.54	0.172	0.104	0.277	6.507	21.746	5.182	0.4462	43.0249
D3_B2_A3-CONF2	4.93	3.54	0.180	0.096	0.277	6.435	21.803	4.952	0.4288	39.8583
**⟨D3_B2_A3⟩**	**4.92**	**3.54**	**0.176**	**0.101**	**0.277**	**6.477**	**21.769**	**5.087**	**0.4390**	**41.7184**
D3_B7_A3-CONF1	4.81	2.97	0.258	0.148	0.406	5.212	28.407	0.323	0.0277	0.0387
D3_B7_A3-CONF2	4.81	2.97	0.256	0.149	0.405	5.188	28.525	0.345	0.0295	0.0444
D3_B7_A3-CONF3	4.81	2.96	0.253	0.155	0.408	4.972	26.051	0.962	0.0900	0.4026
D3_B7_A3-CONF4	4.81	2.96	0.253	0.149	0.401	4.846	25.999	0.844	0.0788	0.3315
D3_B7_A3-CONF5	4.80	2.96	0.257	0.149	0.406	4.968	26.062	0.864	0.0809	0.3310
D3_B7_A3-CONF6	4.81	3.04	0.261	0.148	0.409	4.450	20.842	0.761	0.0868	0.3698
D3_B7_A3-CONF7	4.80	3.04	0.263	0.150	0.412	4.504	20.779	0.908	0.1040	0.5107
D3_B7_A3-CONF8	4.81	3.04	0.263	0.150	0.413	5.229	27.226	0.367	0.0318	0.0477
D3_B7_A3-CONF9	4.80	3.04	0.263	0.145	0.408	4.483	20.861	0.834	0.0948	0.4474
**⟨D3_B7_A3⟩**	**4.81**	**2.98**	**0.258**	**0.150**	**0.407**	**5.014**	**26.459**	**0.593**	**0.0565**	**0.2030**
D4_B1_A1-CONF1	5.36	2.72	0.590	0.765	1.354	2.733	6.015	2.253	1.0176	0.0028
D4_B1_A1-CONF2	5.34	2.69	0.629	0.746	1.375	1.919	5.511	2.185	0.9743	0.0021
D4_B1_A1-CONF3	5.36	2.72	0.588	0.765	1.353	2.311	6.768	2.254	0.8917	0.0022
D4_B1_A1-CONF4	5.37	2.72	0.593	0.768	1.361	2.811	6.679	2.263	0.9585	0.0023
D4_B1_A1-CONF5	5.08	2.98	0.306	0.494	0.800	1.283	7.543	0.023	0.0066	0.0000
D4_B1_A1-CONF6	5.08	2.99	0.340	0.495	0.835	1.024	8.290	0.017	0.0042	0.0000
D4_B1_A1-CONF7	5.36	2.73	0.579	0.209	0.788	3.869	8.627	2.167	0.8360	0.6191
**⟨D4_B1_A1⟩**	**5.13**	**2.94**	**0.363**	**0.456**	**0.819**	**1.637**	**7.874**	**0.398**	**0.1539**	**0.0952**

Continued on next page

Table S5 – Continued from previous page

**Molecule / Conf.**	**IP (eV)**	**EA (eV)**	λ_e (eV)	λ_h (eV)	λ_i (eV)	μ_1 (a.u.)	μ_2 (a.u.)	μ_{12} (a.u.)	V_{12} (eV)	k_{ct} (10^{13} s^{-1})
D4_B1_A2-CONF1	5.36	2.99	0.590	0.147	0.737	6.046	7.954	3.502	1.1524	1.9907
D4_B1_A2-CONF2	5.36	2.90	0.643	0.151	0.794	4.134	5.079	3.476	1.2141	1.2263
D4_B1_A2-CONF3	5.36	2.98	0.592	0.153	0.745	7.086	9.076	3.525	1.1504	1.8252
**⟨D4_B1_A2⟩**	**5.36**	**2.98**	**0.592**	**0.148**	**0.740**	**6.003**	**7.869**	**3.501**	**1.1552**	**1.9481**
D4_B5_A2-CONF2	5.11	2.70	0.287	0.489	0.775	3.608	16.239	0.043	0.0082	0.0001
D4_B5_A2-CONF3	5.11	2.63	0.306	0.497	0.803	2.893	17.870	0.050	0.0084	0.0001
D4_B5_A2-CONF4	5.18	2.59	0.299	0.258	0.557	4.317	15.529	1.451	0.3179	1.0064
D4_B5_A2-CONF5	5.14	2.68	0.304	0.520	0.824	2.765	16.352	0.054	0.0099	0.0001
D4_B5_A2-CONF6	5.13	2.63	0.305	0.512	0.817	3.912	17.912	0.098	0.0176	0.0002
D4_B5_A2-CONF7	5.11	2.63	0.309	0.494	0.803	4.295	18.544	0.049	0.0086	0.0001
D4_B5_A2-CONF9	5.14	2.67	0.306	0.519	0.824	4.153	16.341	0.061	0.0125	0.0001
D4_B5_A2-CONF10	5.13	2.62	0.340	0.518	0.858	5.280	18.756	0.037	0.0069	0.0000
**⟨D4_B5_A2⟩**	**5.12**	**2.64**	**0.304**	**0.498**	**0.802**	**3.260**	**17.603**	**0.057**	**0.0101**	**0.0030**
D5_B7_A2-CONF1	4.76	2.69	0.277	0.246	0.523	2.492	17.826	0.590	0.0827	0.0983
D5_B7_A2-CONF2	4.68	2.68	0.282	0.151	0.433	5.211	23.303	0.594	0.0691	0.1801
D5_B7_A2-CONF3	4.78	2.69	0.282	0.248	0.530	5.067	23.222	0.495	0.0598	0.0476
D5_B7_A2-CONF4	4.78	2.70	0.283	0.245	0.528	5.209	24.946	0.422	0.0469	0.0298
D5_B7_A2-CONF5	4.68	2.70	0.279	0.154	0.434	5.991	23.606	0.836	0.0980	0.3593
D5_B7_A2-CONF6	4.76	2.69	0.280	0.247	0.527	3.307	16.872	0.597	0.0946	0.1222
**⟨D5_B7_A2⟩**	**4.69**	**2.69**	**0.281**	**0.165**	**0.446**	**5.310**	**23.213**	**0.648**	**0.0763**	**0.2123**
D5_B7_A3-CONF1	4.71	2.96	0.256	0.179	0.435	6.734	26.748	0.425	0.0389	0.0561
D5_B7_A3-CONF2	4.68	2.96	0.254	0.154	0.408	6.777	26.785	0.471	0.0431	0.0926
D5_B7_A3-CONF3	4.70	2.95	0.268	0.183	0.451	6.022	24.521	1.071	0.1044	0.3392
D5_B7_A3-CONF4	4.75	3.03	0.257	0.229	0.485	4.649	19.634	1.014	0.1202	0.3105
D5_B7_A3-CONF5	4.69	3.04	0.273	0.152	0.425	6.779	25.363	0.427	0.0399	0.0656
**⟨D5_B7_A3⟩**	**4.69**	**3.01**	**0.268**	**0.159**	**0.427**	**6.655**	**25.465**	**0.490**	**0.0468**	**0.0929**

2.4 ωB97XD/6-31G(d,p)

Table S6: Hole–electron overlap, spatial charge-transfer, and frontier-orbital/photophysical parameters for the conformational ensemble of the ten selected sensitizers at ωB97XD/6-31G(d,p)/SMD(acetonitrile). The Boltzmann-weighted ensemble average is shown in the highlighted row beneath each conformer set, labeled ⟨Molecule⟩.

**Molecule / Conf.**	**Pop. (%)**	S_r	D (Å)	t (Å)	**HOMO (eV)**	**LUMO (eV)**	**Gap (eV)**	$\lambda_{\max}$ (nm)	f
D2_B1_A1-CONF1	59.96	0.479	3.034	1.287	-8.02	-0.95	7.07	344.8	0.1800
D2_B1_A1-CONF2	22.32	0.495	3.046	1.228	-8.02	-0.98	7.04	348.3	0.1961
D2_B1_A1-CONF3	17.73	0.505	2.906	1.117	-8.02	-1.00	7.03	348.4	0.1978
**⟨D2_B1_A1⟩**	–	**0.487**	**3.014**	**1.244**	**-8.02**	**-0.97**	**7.05**	**346.2**	**0.1867**
D3_B1_A1-CONF1	1.68	0.658	2.728	0.020	-7.37	-0.86	6.51	354.1	0.6763
D3_B1_A1-CONF2	0.63	0.657	2.719	-0.008	-7.37	-0.91	6.46	357.8	0.6973
D3_B1_A1-CONF3	1.19	0.659	2.734	0.057	-7.38	-0.86	6.52	351.2	0.6503
D3_B1_A1-CONF4	0.54	0.658	2.765	0.054	-7.39	-0.90	6.48	353.9	0.6651
D3_B1_A1-CONF5	40.26	0.658	2.762	0.013	-7.38	-0.92	6.45	357.2	0.6963
D3_B1_A1-CONF6	22.73	0.654	2.820	0.062	-7.39	-0.92	6.47	354.6	0.6645
D3_B1_A1-CONF7	32.97	0.648	2.784	0.041	-7.38	-0.92	6.47	356.8	0.6705
**⟨D3_B1_A1⟩**	–	**0.653**	**2.781**	**0.034**	**-7.38**	**-0.92**	**6.46**	**356.3**	**0.6795**
D3_B2_A1-CONF1	0.20	0.690	2.279	-0.955	-7.35	-1.36	5.99	390.8	0.8906
D3_B2_A1-CONF2	0.81	0.673	2.428	-0.619	-7.36	-1.32	6.04	385.4	0.8487
D3_B2_A1-CONF3	0.08	0.684	2.307	-0.894	-7.36	-1.36	6.00	391.8	0.8800
D3_B2_A1-CONF4	0.57	0.690	2.274	-0.894	-7.37	-1.33	6.04	383.2	0.8323
D3_B2_A1-CONF5	4.00	0.687	2.278	-0.913	-7.35	-1.35	6.01	388.2	0.8632
D3_B2_A1-CONF6	30.10	0.687	2.365	-0.817	-7.37	-1.35	6.02	385.6	0.8502
D3_B2_A1-CONF7	43.86	0.692	2.313	-0.900	-7.35	-1.37	5.98	390.2	0.8881
D3_B2_A1-CONF8	20.39	0.687	2.333	-0.859	-7.37	-1.37	6.00	389.0	0.8663
**⟨D3_B2_A1⟩**	–	**0.689**	**2.332**	**-0.865**	**-7.36**	**-1.36**	**6.00**	**388.4**	**0.8706**
D3_B2_A3-CONF1	58.74	0.658	1.966	-2.059	-7.32	-1.79	5.53	460.1	1.7495
D3_B2_A3-CONF2	41.26	0.656	1.913	-2.093	-7.33	-1.78	5.56	457.9	1.7290
**⟨D3_B2_A3⟩**	–	**0.657**	**1.944**	**-2.073**	**-7.32**	**-1.78**	**5.54**	**459.2**	**1.7410**
D3_B7_A3-CONF1	16.52	0.628	0.886	-2.112	-7.28	-0.94	6.34	357.4	1.4617
D3_B7_A3-CONF2	27.51	0.625	0.940	-2.060	-7.28	-0.94	6.34	357.8	1.4405
D3_B7_A3-CONF3	13.58	0.641	0.842	-2.173	-7.27	-0.94	6.33	359.8	1.3918

Continued on next page

Table S6 – Continued from previous page

**Molecule / Conf.**	**Pop. (%)**	S_r	D (Å)	t (Å)	**HOMO (eV)**	**LUMO (eV)**	**Gap (eV)**	$\lambda_{\max}$ (nm)	f
D3_B7_A3-CONF4	5.94	0.648	0.773	-2.193	-7.27	-0.93	6.33	358.6	1.4023
D3_B7_A3-CONF5	12.28	0.638	0.791	-2.176	-7.28	-0.94	6.34	359.1	1.3924
D3_B7_A3-CONF6	5.61	0.664	0.839	-1.541	-7.27	-1.02	6.25	361.9	1.1981
D3_B7_A3-CONF7	5.18	0.664	0.904	-1.422	-7.26	-1.02	6.24	363.2	1.1684
D3_B7_A3-CONF8	9.42	0.641	0.965	-1.938	-7.28	-1.02	6.26	360.6	1.4200
D3_B7_A3-CONF9	3.96	0.664	0.851	-1.415	-7.26	-1.02	6.25	362.3	1.2000
**⟨D3_B7_A3⟩**	–	**0.638**	**0.881**	**-2.007**	**-7.27**	**-0.96**	**6.32**	**359.2**	**1.3901**
D4_B1_A1-CONF1	0.64	0.640	1.816	0.151	-7.89	-0.72	7.17	373.6	0.3185
D4_B1_A1-CONF2	0.71	0.630	1.857	0.355	-7.88	-0.69	7.19	372.2	0.3039
D4_B1_A1-CONF3	0.30	0.634	1.821	0.152	-7.89	-0.73	7.16	373.8	0.3207
D4_B1_A1-CONF4	0.46	0.637	1.810	0.130	-7.90	-0.73	7.17	373.6	0.3211
D4_B1_A1-CONF5	56.43	0.150	4.027	2.713	-7.65	-0.97	6.68	361.4	0.0001
D4_B1_A1-CONF6	26.08	0.152	4.031	2.707	-7.65	-1.00	6.65	364.1	0.0000
D4_B1_A1-CONF7	15.37	0.619	1.859	0.205	-7.90	-0.74	7.16	374.4	0.3123
**⟨D4_B1_A1⟩**	–	**0.233**	**3.648**	**2.273**	**-7.69**	**-0.94**	**6.76**	**364.4**	**0.0547**
D4_B1_A2-CONF1	91.03	0.662	1.072	-1.524	-7.78	-1.07	6.71	410.2	0.9677
D4_B1_A2-CONF2	4.63	0.676	1.220	-0.758	-7.79	-0.97	6.83	398.5	0.8858
D4_B1_A2-CONF3	4.34	0.662	1.109	-1.480	-7.78	-1.07	6.71	409.8	0.9658
**⟨D4_B1_A2⟩**	–	**0.662**	**1.080**	**-1.487**	**-7.78**	**-1.07**	**6.72**	**409.7**	**0.9638**
D4_B5_A2-CONF2	16.66	0.656	0.931	-0.372	-7.67	-0.66	7.01	313.0	1.2172
D4_B5_A2-CONF3	62.85	0.660	0.655	-0.824	-7.68	-0.58	7.10	318.8	1.1986
D4_B5_A2-CONF4	0.29	0.628	0.806	-1.679	-7.70	-0.55	7.15	324.3	1.1578
D4_B5_A2-CONF5	3.35	0.660	0.935	-0.394	-7.71	-0.63	7.08	312.1	1.1699
D4_B5_A2-CONF6	9.00	0.639	0.586	-0.704	-7.70	-0.57	7.13	319.0	1.1691
D4_B5_A2-CONF7	3.03	0.660	0.643	-0.683	-7.67	-0.59	7.08	318.4	1.2309
D4_B5_A2-CONF9	0.16	0.647	0.948	-0.656	-7.70	-0.64	7.06	311.3	1.2143
D4_B5_A2-CONF10	4.66	0.663	0.636	-0.595	-7.70	-0.58	7.12	318.5	1.2019
**⟨D4_B5_A2⟩**	–	**0.657**	**0.704**	**-0.711**	**-7.68**	**-0.60**	**7.08**	**317.6**	**1.1992**
D5_B7_A2-CONF1	3.68	0.646	1.075	-1.212	-7.25	-0.68	6.57	332.5	1.0027
D5_B7_A2-CONF2	58.83	0.635	0.999	-1.252	-7.16	-0.68	6.49	330.8	1.2702

Continued on next page

Table S6 – Continued from previous page

Molecule / Conf.	Pop. (%)	S_r	D (Å)	t (Å)	HOMO (eV)	LUMO (eV)	Gap (eV)	$\lambda_{\max}$ (nm)	f
D5_B7_A2-CONF3	7.16	0.636	0.998	-1.248	-7.27	-0.67	6.60	330.5	1.2445
D5_B7_A2-CONF4	2.81	0.638	0.987	-1.410	-7.28	-0.69	6.59	331.0	1.1625
D5_B7_A2-CONF5	27.33	0.662	0.984	-1.328	-7.16	-0.69	6.47	333.6	1.2104
D5_B7_A2-CONF6	0.18	0.642	1.133	-1.100	-7.25	-0.69	6.57	332.4	1.0095
**⟨D5_B7_A2⟩**	–	**0.643**	**0.998**	**-1.275**	**-7.17**	**-0.68**	**6.49**	**331.6**	**1.2387**
D5_B7_A3-CONF1	10.07	0.639	0.917	-2.130	-7.17	-0.93	6.24	358.0	1.3707
D5_B7_A3-CONF2	15.27	0.643	0.991	-2.104	-7.16	-0.93	6.23	358.4	1.3591
D5_B7_A3-CONF3	5.56	0.644	0.942	-2.078	-7.15	-0.93	6.22	360.3	1.3444
D5_B7_A3-CONF4	3.62	0.654	0.919	-1.479	-7.24	-1.01	6.23	363.3	1.1962
D5_B7_A3-CONF5	65.48	0.660	0.919	-2.063	-7.16	-1.02	6.15	361.3	1.3545
**⟨D5_B7_A3⟩**	–	**0.654**	**0.931**	**-2.056**	**-7.16**	**-0.99**	**6.17**	**360.6**	**1.3505**

Table S7: Redox potentials, reorganization energies, dipole moments, electronic coupling (V_{12}), and Marcus charge-transfer rate constant (k_{ct}) for ωB97XD/6-31G(d,p)/SMD(acetonitrile). The Boltzmann-weighted ensemble average is shown in the highlighted row beneath each conformer set, labeled ⟨Molecule⟩.

Molecule / Conf.	IP (eV)	EA (eV)	λ_e (eV)	λ_h (eV)	λ_i (eV)	μ_1 (a.u.)	μ_2 (a.u.)	μ_{12} (a.u.)	V_{12} (eV)	k_{ct} (10^{13} s^{-1})
D2_B1_A1-CONF1	5.97	3.20	0.332	0.106	0.438	0.630	6.021	1.504	0.8758	27.4311
D2_B1_A1-CONF2	5.97	3.24	0.358	0.115	0.472	0.985	7.170	1.611	0.8223	16.6619
D2_B1_A1-CONF3	5.98	3.24	0.355	0.106	0.461	0.987	6.917	1.636	0.8599	20.5365
**⟨D2_B1_A1⟩**	**5.97**	**3.22**	**0.342**	**0.108**	**0.450**	**0.773**	**6.436**	**1.551**	**0.8610**	**23.8054**
D3_B1_A1-CONF1	5.35	3.10	0.329	0.116	0.445	1.157	7.333	3.010	1.2220	49.6813
D3_B1_A1-CONF2	5.36	3.13	0.310	0.117	0.428	1.894	8.427	3.013	1.1748	55.2676
D3_B1_A1-CONF3	5.36	3.10	0.341	0.115	0.456	1.143	7.212	2.921	1.2243	44.3374
D3_B1_A1-CONF4	5.36	3.13	0.313	0.114	0.427	1.878	8.425	2.962	1.1752	55.5440
D3_B1_A1-CONF5	5.36	3.14	0.308	0.113	0.421	3.770	9.614	3.034	1.2500	67.0280
D3_B1_A1-CONF6	5.36	3.15	0.310	0.113	0.423	3.725	9.563	2.980	1.2489	65.4040
D3_B1_A1-CONF7	5.36	3.14	0.311	0.114	0.425	3.670	9.715	2.954	1.2143	61.0579
**⟨D3_B1_A1⟩**	**5.36**	**3.14**	**0.310**	**0.113**	**0.424**	**3.629**	**9.555**	**2.993**	**1.2363**	**63.9930**

Continued on next page

Table S7 – Continued from previous page

**Molecule / Conf.**	**IP (eV)**	**EA (eV)**	λ_e (eV)	λ_h (eV)	λ_i (eV)	μ_1 (a.u.)	μ_2 (a.u.)	μ_{12} (a.u.)	V_{12} (eV)	k_{ct} (10^{13} s^{-1})
D3_B2_A1-CONF1	5.36	3.33	0.208	0.112	0.320	2.074	8.571	3.606	1.1786	182.9327
D3_B2_A1-CONF2	5.35	3.29	0.217	0.111	0.328	1.870	8.620	3.376	1.1375	156.7005
D3_B2_A1-CONF3	5.36	3.32	0.216	0.112	0.329	2.148	8.668	3.554	1.1660	162.8587
D3_B2_A1-CONF4	5.36	3.30	0.222	0.112	0.334	1.860	7.864	3.480	1.2251	169.2252
D3_B2_A1-CONF5	5.36	3.32	0.207	0.112	0.319	2.788	9.232	3.535	1.1803	185.2047
D3_B2_A1-CONF6	5.36	3.33	0.209	0.112	0.321	1.801	8.050	3.468	1.1944	186.8252
D3_B2_A1-CONF7	5.36	3.34	0.206	0.111	0.317	3.387	9.367	3.596	1.2216	203.7833
D3_B2_A1-CONF8	5.36	3.34	0.207	0.111	0.318	3.400	9.332	3.531	1.2203	201.2106
**⟨D3_B2_A1⟩**	**5.36**	**3.33**	**0.207**	**0.111**	**0.319**	**2.863**	**8.941**	**3.539**	**1.2107**	**196.7591**
D3_B2_A3-CONF1	5.37	3.70	0.256	0.112	0.367	5.485	15.092	4.646	0.9367	68.3139
D3_B2_A3-CONF2	5.37	3.70	0.261	0.106	0.367	5.477	14.899	4.613	0.9472	69.9652
**⟨D3_B2_A3⟩**	**5.37**	**3.70**	**0.258**	**0.109**	**0.367**	**5.481**	**15.012**	**4.632**	**0.9411**	**68.9953**
D3_B7_A3-CONF1	5.32	3.14	0.340	0.161	0.501	4.919	12.766	3.801	1.2068	26.4555
D3_B7_A3-CONF2	5.32	3.14	0.338	0.161	0.498	4.894	13.109	3.870	1.1881	26.3512
D3_B7_A3-CONF3	5.32	3.14	0.334	0.167	0.501	4.676	12.669	3.889	1.2017	26.2814
D3_B7_A3-CONF4	5.32	3.14	0.333	0.161	0.495	4.569	13.029	3.918	1.1746	26.7033
D3_B7_A3-CONF5	5.32	3.14	0.338	0.164	0.503	4.686	12.321	3.908	1.2350	27.1668
D3_B7_A3-CONF6	5.31	3.23	0.343	0.162	0.505	4.181	7.864	3.750	1.5375	40.9804
D3_B7_A3-CONF7	5.31	3.23	0.346	0.162	0.508	4.221	8.211	3.711	1.5032	38.1313
D3_B7_A3-CONF8	5.32	3.23	0.346	0.164	0.510	4.923	11.774	3.800	1.2767	26.8596
D3_B7_A3-CONF9	5.31	3.23	0.346	0.160	0.506	4.207	8.275	3.763	1.5054	38.7973
**⟨D3_B7_A3⟩**	**5.32**	**3.16**	**0.339**	**0.162**	**0.502**	**4.724**	**12.026**	**3.843**	**1.2548**	**28.4517**
D4_B1_A1-CONF1	5.80	3.00	0.608	0.772	1.379	2.564	4.860	2.205	1.4718	0.0046
D4_B1_A1-CONF2	5.77	2.96	0.641	0.745	1.386	1.832	4.602	2.141	1.3986	0.0039
D4_B1_A1-CONF3	5.79	2.99	0.609	0.767	1.376	2.139	5.804	2.206	1.2757	0.0036
D4_B1_A1-CONF4	5.79	2.99	0.611	0.762	1.373	2.699	5.817	2.207	1.3555	0.0042
D4_B1_A1-CONF5	5.58	3.23	0.353	0.560	0.913	1.290	6.615	0.039	0.0253	0.0002
D4_B1_A1-CONF6	5.58	3.23	0.381	0.560	0.942	1.020	7.552	0.040	0.0208	0.0001
D4_B1_A1-CONF7	5.80	3.01	0.599	0.160	0.759	3.749	7.884	2.133	1.1890	1.6879
**⟨D4_B1_A1⟩**	**5.62**	**3.19**	**0.404**	**0.503**	**0.907**	**1.618**	**7.023**	**0.406**	**0.2319**	**0.2597**

Continued on next page

Table S7 – Continued from previous page

**Molecule / Conf.**	**IP (eV)**	**EA (eV)**	λ_e (eV)	λ_h (eV)	λ_i (eV)	μ_1 (a.u.)	μ_2 (a.u.)	μ_{12} (a.u.)	V_{12} (eV)	k_{ct} (10^{13} s^{-1})
D4_B1_A2-CONF1	5.79	3.18	0.637	0.135	0.772	5.907	8.303	3.647	1.4357	2.1547
D4_B1_A2-CONF2	5.79	3.10	0.681	0.152	0.833	3.896	4.998	3.460	1.5361	1.3058
D4_B1_A2-CONF3	5.79	3.17	0.639	0.144	0.783	7.004	9.381	3.682	1.4396	1.9356
**⟨D4_B1_A2⟩**	**5.79**	**3.17**	**0.639**	**0.136**	**0.775**	**5.861**	**8.196**	**3.640**	**1.4405**	**2.1059**
D4_B5_A2-CONF2	5.61	2.90	0.346	0.554	0.901	3.741	1.444	3.583	1.8859	0.9855
D4_B5_A2-CONF3	5.61	2.84	0.367	0.563	0.930	2.847	2.916	3.174	1.9447	0.7750
D4_B5_A2-CONF4	5.70	2.81	0.357	0.318	0.675	4.242	5.335	3.224	1.8849	10.1857
D4_B5_A2-CONF5	5.64	2.87	0.363	0.590	0.953	2.791	1.983	3.566	1.9738	0.6311
D4_B5_A2-CONF6	5.63	2.83	0.364	0.581	0.945	3.969	2.049	3.189	1.8609	0.6078
D4_B5_A2-CONF7	5.61	2.84	0.370	0.561	0.931	4.282	4.421	3.319	1.9464	0.7663
D4_B5_A2-CONF9	5.64	2.88	0.365	0.589	0.955	4.210	3.758	3.659	1.9874	0.6274
D4_B5_A2-CONF10	5.64	2.84	0.408	0.587	0.996	5.373	3.354	3.298	1.8612	0.3620
**⟨D4_B5_A2⟩**	**5.62**	**2.85**	**0.365**	**0.564**	**0.930**	**3.263**	**2.636**	**3.268**	**1.9244**	**0.7977**
D5_B7_A2-CONF1	5.16	2.89	0.347	0.285	0.633	2.449	6.162	3.375	1.6333	11.9601
D5_B7_A2-CONF2	5.07	2.89	0.351	0.178	0.529	4.999	9.341	3.541	1.5977	34.2440
D5_B7_A2-CONF3	5.18	2.89	0.352	0.287	0.640	4.877	9.286	3.582	1.5973	10.6292
D5_B7_A2-CONF4	5.18	2.90	0.352	0.285	0.636	4.982	9.355	3.346	1.5679	10.5787
D5_B7_A2-CONF5	5.06	2.90	0.348	0.180	0.528	5.782	9.961	3.486	1.5938	34.5845
D5_B7_A2-CONF6	5.16	2.89	0.351	0.287	0.638	3.403	5.419	3.408	1.7884	13.5520
**⟨D5_B7_A2⟩**	**5.08**	**2.89**	**0.350**	**0.193**	**0.544**	**5.107**	**9.383**	**3.517**	**1.5974**	**31.1236**
D5_B7_A3-CONF1	5.09	3.13	0.337	0.203	0.540	6.296	12.901	3.519	1.2625	19.0163
D5_B7_A3-CONF2	5.07	3.13	0.335	0.180	0.515	6.331	13.011	3.580	1.2649	24.9879
D5_B7_A3-CONF3	5.09	3.13	0.350	0.207	0.557	5.614	13.049	3.523	1.1834	14.0124
D5_B7_A3-CONF4	5.15	3.22	0.338	0.268	0.606	4.330	8.291	3.851	1.5175	13.6040
D5_B7_A3-CONF5	5.07	3.22	0.356	0.179	0.535	6.325	11.722	3.961	1.4179	25.3753
**⟨D5_B7_A3⟩**	**5.07**	**3.19**	**0.350**	**0.186**	**0.536**	**6.211**	**11.987**	**3.830**	**1.3695**	**23.6179**

2.5 Averaging-Order Comparison: Property-of-the-Average versus Average-of-the-Property

Table S8 reports, for all ten top-ranked sensitizers at each of the three functionals, a direct comparison between the property-of-the-average and average-of-the-property definitions of LHE, $J_{\mathrm{SC-max}}$, V_{12}, and k_{CT}. Property-of-the-average denotes computing the quantity once from the Boltzmann-averaged underlying descriptors (f for LHE; f, λ_{max} for $J_{\mathrm{SC-max}}$; μ_1, μ_2, μ_{12}, the transition energy, and λ_i for V_{12} and k_{CT}); average-of-the-property denotes computing the quantity independently for every conformer and then Boltzmann-averaging the resulting per-conformer values. The manuscript reports average-of-the-property throughout, since it is the formally correct ensemble average for any observable; this table verifies that the simpler property-of-the-average would have been an adequate approximation for LHE, $J_{\mathrm{SC-max}}$, and V_{12}, but not for k_{CT}, as detailed in the main text.

Table S8: Property-of-the-average versus average-of-the-property comparison for LHE, $J_{\mathrm{SC-max}}$, V_{12}, and k_{CT}, ten top-ranked sensitizers, three functionals. $\Delta\% = (\mathrm{AOP} - \mathrm{POA})/\mathrm{POA} \times 100$. $J_{\mathrm{SC-max}}$ in $\mathrm{mA\,cm^{-2}}$; k_{CT} in units of $10^{13}\,\mathrm{s^{-1}}$.

**Molecule**	**Functional**	$\mathrm{LHE}^{\mathrm{POA}}$	$\mathrm{LHE}^{\mathrm{AOP}}$	ΔLHE (%)	$J_{\text{SC-max}}^{\mathrm{POA}}$	$J_{\text{SC-max}}^{\mathrm{AOP}}$	$\Delta J_{\text{SC-max}}$ (%)	V_{12}^{POA}	V_{12}^{AOP}	ΔV_{12} (%)	$k_{\mathrm{CT}}^{\mathrm{POA}}$	$k_{\mathrm{CT}}^{\mathrm{AOP}}$	Δk_{CT} (%)
D2_B1_A1	B3LYP	0.122	0.122	-0.0	1.743	1.743	+0.0	0.396	0.396	+0.0	6.688	6.718	+0.4
D2_B1_A1	CAM-B3LYP	0.230	0.230	-0.0	0.872	0.874	+0.2	0.596	0.597	+0.2	9.896	9.939	+0.4
D2_B1_A1	ωB97XD	0.349	0.349	-0.0	0.445	0.446	+0.2	0.860	0.861	+0.1	23.276	23.805	+2.3
D3_B1_A1	B3LYP	0.413	0.412	-0.1	7.715	7.703	-0.2	0.386	0.386	-0.0	11.603	11.653	+0.4
D3_B1_A1	CAM-B3LYP	0.666	0.666	-0.0	3.336	3.336	-0.0	0.859	0.859	-0.0	37.576	37.655	+0.2
D3_B1_A1	ωB97XD	0.791	0.791	-0.0	1.405	1.406	+0.0	1.236	1.236	-0.0	63.872	63.993	+0.2
D3_B2_A1	B3LYP	0.504	0.503	-0.1	9.829	9.818	-0.1	0.354	0.354	+0.0	31.989	32.088	+0.3
D3_B2_A1	CAM-B3LYP	0.767	0.767	-0.0	6.230	6.230	+0.0	0.753	0.753	-0.0	94.196	94.331	+0.1
D3_B2_A1	ωB97XD	0.865	0.865	-0.0	3.129	3.131	+0.0	1.211	1.211	-0.0	196.666	196.759	+0.0
D3_B2_A3	B3LYP	0.882	0.882	-0.1	13.989	13.983	-0.0	0.439	0.439	-0.0	41.715	41.718	+0.0
D3_B2_A3	CAM-B3LYP	0.969	0.969	-0.0	15.165	15.164	-0.0	0.781	0.781	-0.0	66.965	66.972	+0.0
D3_B2_A3	ωB97XD	0.982	0.982	-0.0	9.887	9.888	+0.0	0.941	0.941	+0.0	68.989	68.995	+0.0
D3_B7_A3	B3LYP	0.047	0.047	-1.4	0.919	0.905	-1.5	0.054	0.057	+4.4	0.147	0.203	+38.1
D3_B7_A3	CAM-B3LYP	0.906	0.903	-0.4	3.182	3.167	-0.4	0.998	1.000	+0.2	24.634	24.740	+0.4
D3_B7_A3	ωB97XD	0.959	0.958	-0.1	2.088	2.085	-0.1	1.251	1.255	+0.3	28.194	28.452	+0.9
D4_B1_A1	B3LYP	0.065	0.055	-14.1	1.297	0.620	-52.2	0.120	0.154	+27.9	0.009	0.095	+924.2
D4_B1_A1	CAM-B3LYP	0.098	0.078	-20.4	0.650	0.361	-44.4	0.203	0.208	+2.5	0.014	0.186	+1232.1
D4_B1_A1	ωB97XD	0.118	0.090	-24.1	0.227	0.222	-2.4	0.253	0.232	-8.4	0.017	0.260	+1459.8
D4_B1_A2	B3LYP	0.709	0.709	-0.0	9.290	9.290	+0.0	1.155	1.155	-0.0	1.940	1.948	+0.4
D4_B1_A2	CAM-B3LYP	0.854	0.854	-0.0	6.381	6.382	+0.0	1.337	1.337	-0.0	1.947	1.956	+0.5
D4_B1_A2	ωB97XD	0.891	0.891	-0.0	4.622	4.624	+0.0	1.441	1.440	-0.0	2.097	2.106	+0.4
D4_B5_A2	B3LYP	0.001	0.001	-5.7	0.010	0.009	-9.3	0.010	0.010	+2.2	0.000	0.003	+3922.8
D4_B5_A2	CAM-B3LYP	0.927	0.927	-0.0	0.908	0.910	+0.2	1.865	1.842	-1.2	1.070	1.073	+0.3
D4_B5_A2	ωB97XD	0.937	0.937	-0.0	0.569	0.570	+0.2	1.943	1.924	-1.0	0.777	0.798	+2.7
D5_B7_A2	B3LYP	0.045	0.045	-0.4	0.780	0.784	+0.5	0.076	0.076	+0.1	0.190	0.212	+12.0
D5_B7_A2	CAM-B3LYP	0.490	0.450	-8.3	0.870	0.843	-3.1	0.563	0.570	+1.3	5.158	5.408	+4.9
D5_B7_A2	ωB97XD	0.942	0.942	-0.1	0.938	0.937	-0.0	1.597	1.597	-0.0	29.314	31.124	+6.2
D5_B7_A3	B3LYP	0.024	0.024	-1.0	0.475	0.470	-1.0	0.046	0.047	+1.6	0.086	0.093	+8.4
D5_B7_A3	CAM-B3LYP	0.494	0.474	-4.0	1.868	1.787	-4.3	0.480	0.484	+0.7	4.191	4.307	+2.8
D5_B7_A3	ωB97XD	0.955	0.955	-0.0	2.136	2.136	+0.0	1.373	1.369	-0.2	23.462	23.618	+0.7

Across the 30 (molecule, functional) combinations, LHE deviates by 2.7% on average and by up to 24.1% (D4_B1_A1/ωB97XD); $J_{\mathrm{SC-max}}$ deviates by 4.0% on average and by up to 52.2% (D4_B1_A1/B3LYP); V_{12} deviates by 1.7% on average and by up to 27.9%; and k_{CT} deviates by 254% on average and by up to 3923% (D4_B5_A2/B3LYP). In every case, the largest deviations are confined to the same dyes identified elsewhere in this work as having a dark or near-dark dominant conformer (D4-B1-A1 and, at the B3LYP level, D4-B5-A2), for which a small population of highly coupled conformers dominates a population-weighted average of an already-nonlinear (squared and exponentiated) per-conformer quantity far more than it shifts a linear average of the underlying inputs. LHE and $J_{\mathrm{SC-max}}$, which depend on f alone through a smooth, non-exponentiated function, show comparably modest sensitivity to the averaging order; property-of-the-average would have been an adequate approximation for both, though the manuscript reports average-of-the-property throughout for consistency and formal correctness.

2.6 Gas-Phase Screening of the 150-Dye Library (CAM-B3LYP/6-31G(d,p)//r^{2}SCAN-3c)

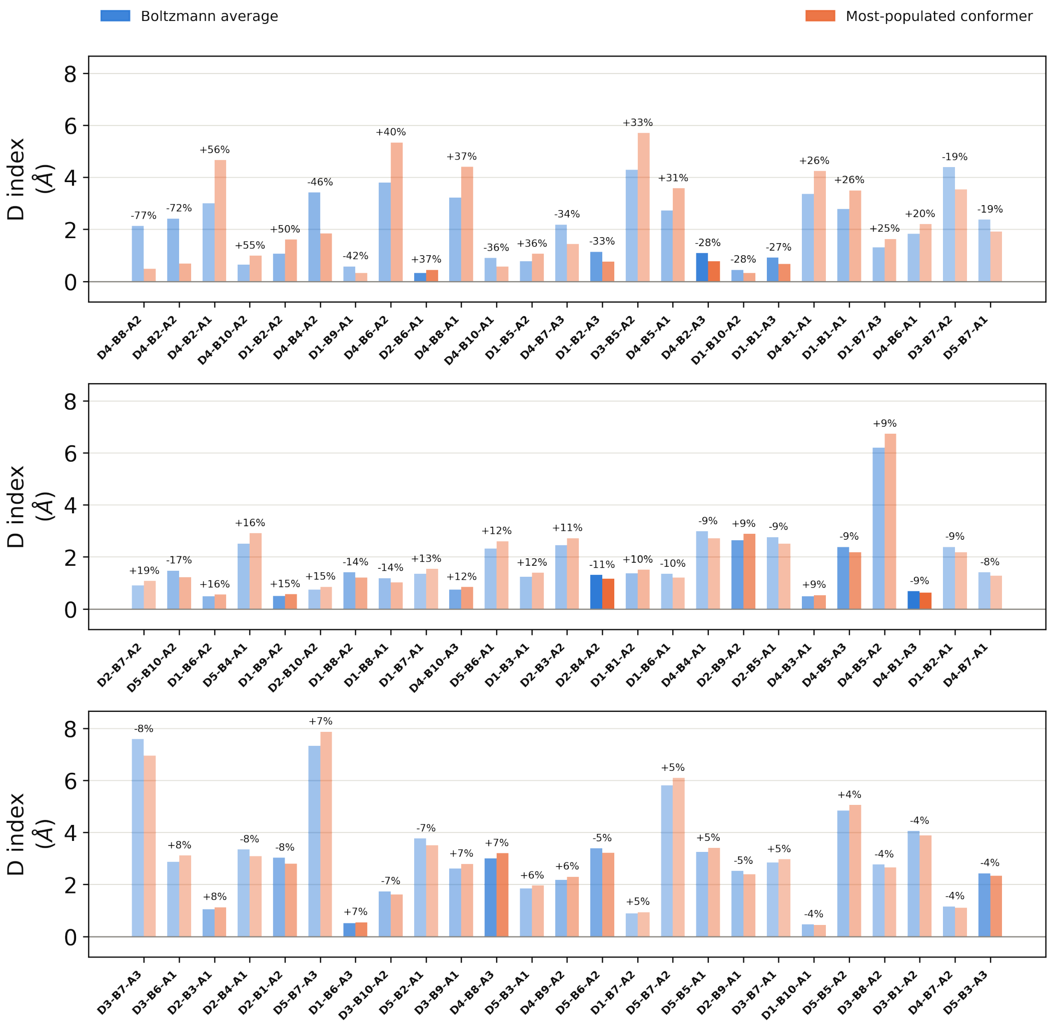

Figure S1: Boltzmann-averaged D index versus the value obtained from only the most-populated conformer of each dye, at the CAM-B3LYP/6-31G(d,p)//r^{2}SCAN-3c gas-phase level. Bar opacity is proportional to the Boltzmann weight of the most-populated conformer (darker = a single conformer dominates the ensemble; lighter = the population is spread across several conformers). The percentage above each pair is the deviation of the most-populated-conformer value relative to the Boltzmann average, $\Delta D = (D^{\text{best}} - \langle D \rangle)/\langle D \rangle$. Dyes are sorted by $|\Delta\%|$ in descending order; this figure shows the 75 dyes with the largest deviations.

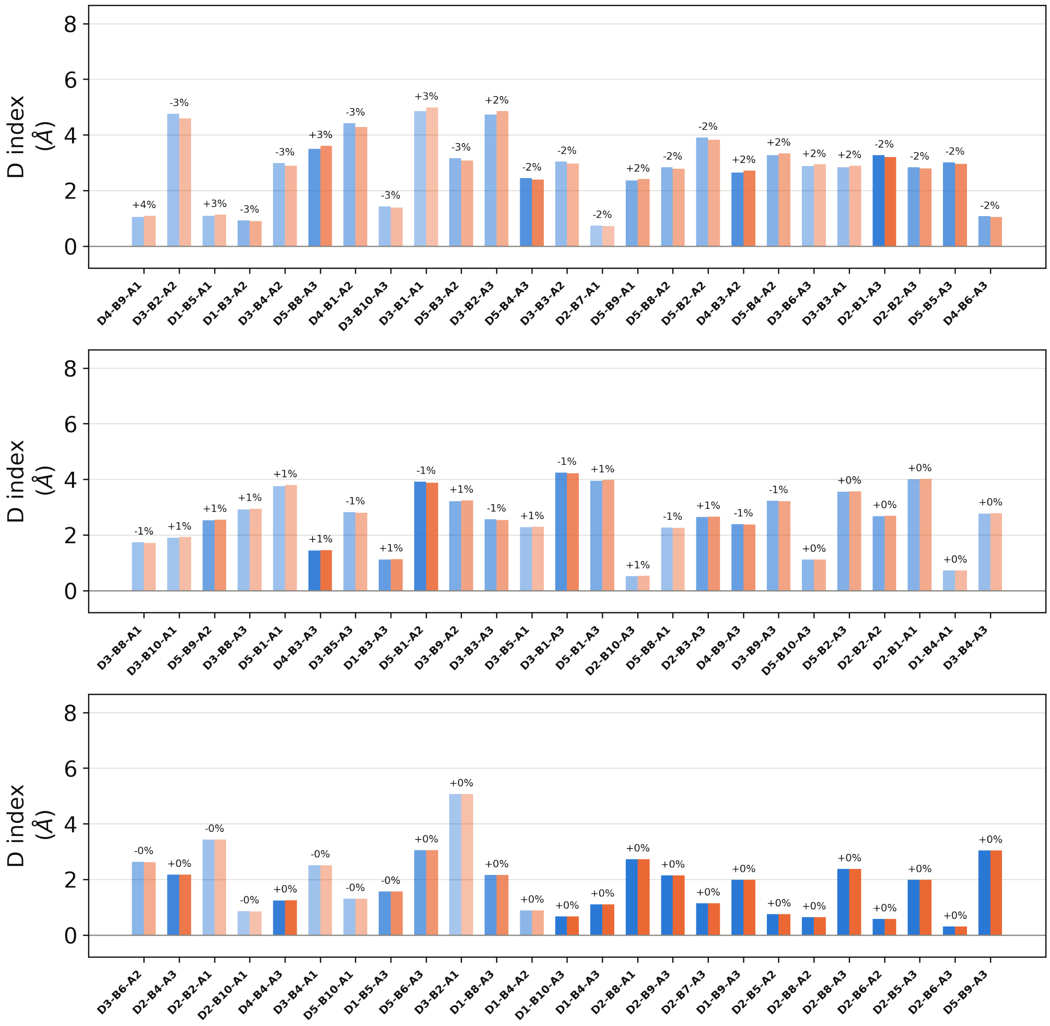

Figure S2: Continuation of Figure S1, showing the remaining 75 (of 150) dyes, in the same order of decreasing $|\Delta\%|$.

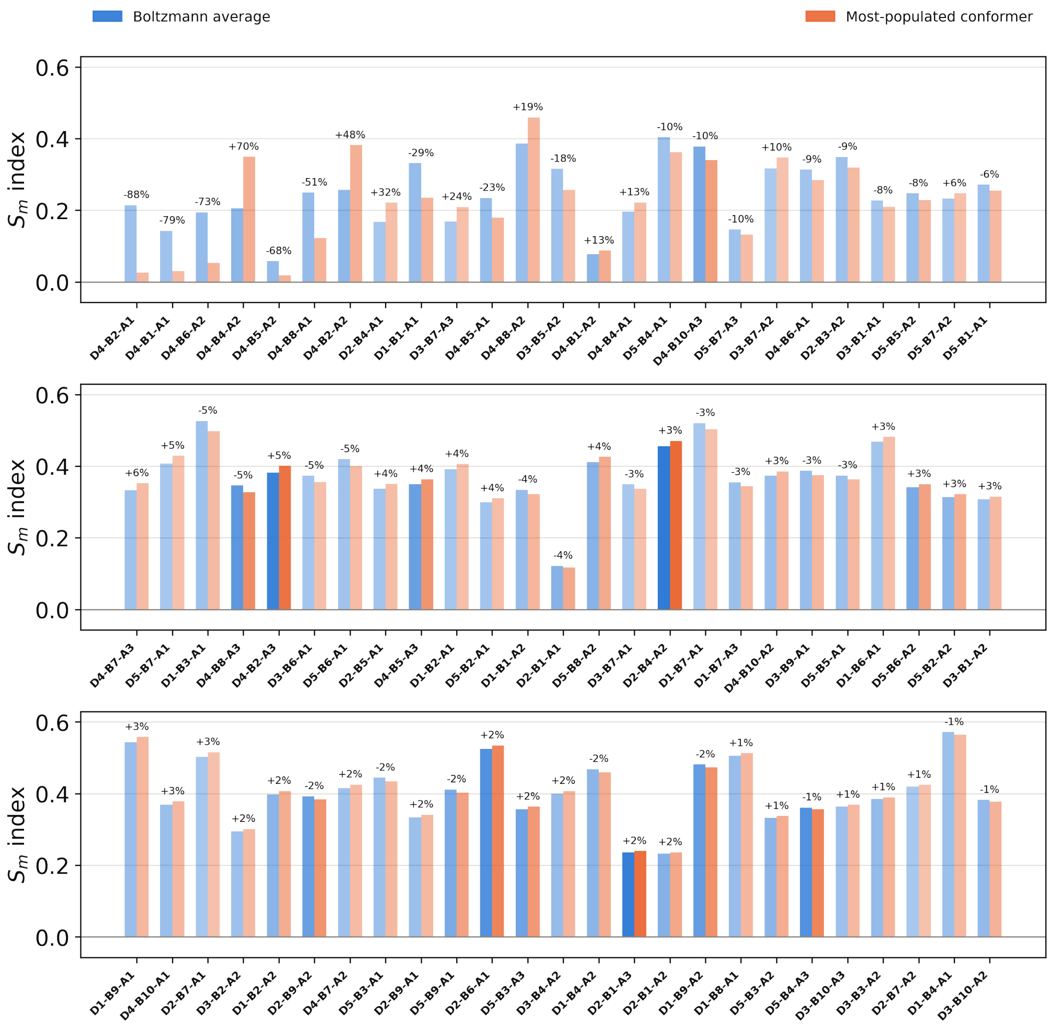

Figure S3: Boltzmann-averaged S_m index versus the value obtained from only the most-populated conformer of each dye, at the CAM-B3LYP/6-31G(d,p)//r^{2}SCAN-3c gas-phase level. Bar opacity is proportional to the Boltzmann weight of the most-populated conformer (darker = a single conformer dominates the ensemble; lighter = the population is spread across several conformers). The percentage above each pair is the deviation of the most-populated-conformer value relative to the Boltzmann average, $\Delta S_m = (S_m^{\text{best}} - \langle S_m \rangle)/\langle S_m \rangle$. Dyes are sorted by $|\Delta\%|$ in descending order; this figure shows the 75 dyes with the largest deviations.

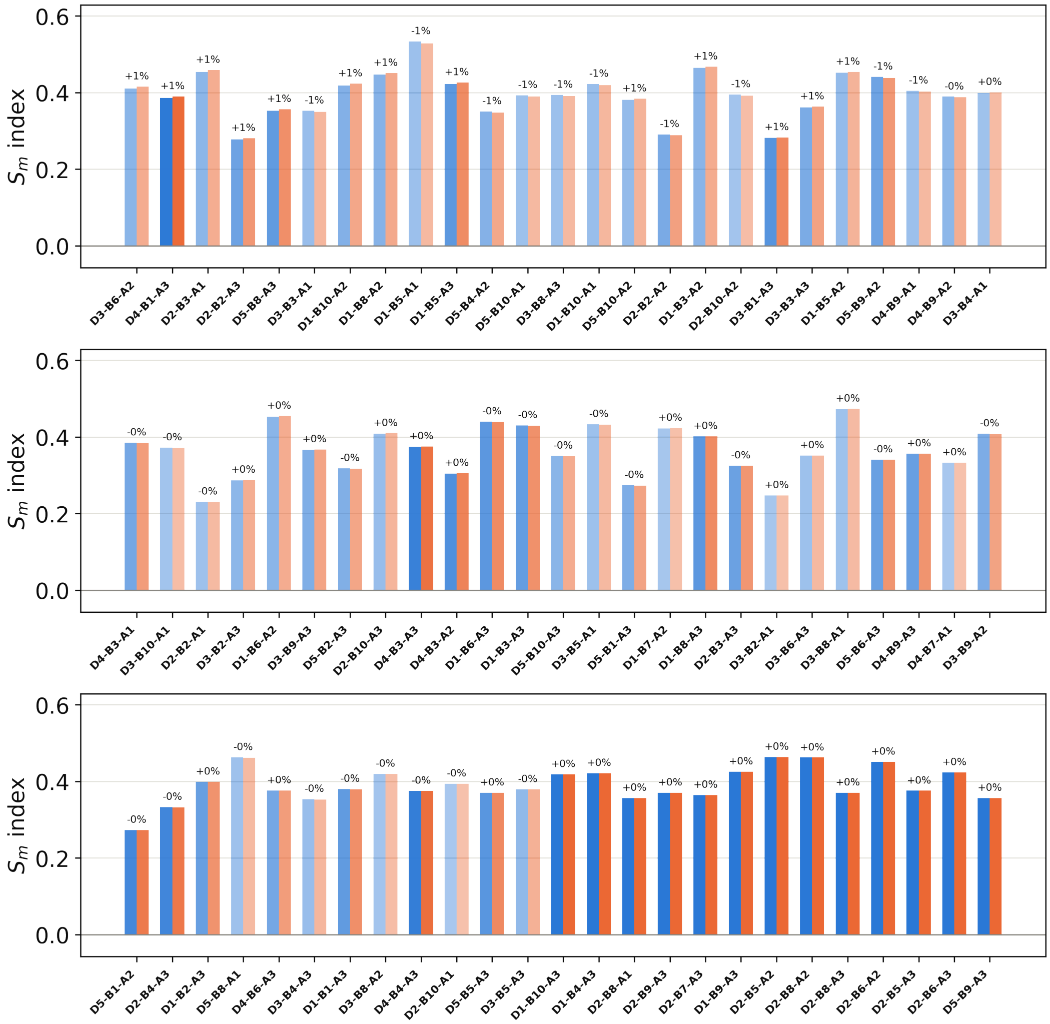

Figure S4: Continuation of Figure S3, showing the remaining 75 (of 150) dyes, in the same order of decreasing $|\Delta\%|$.

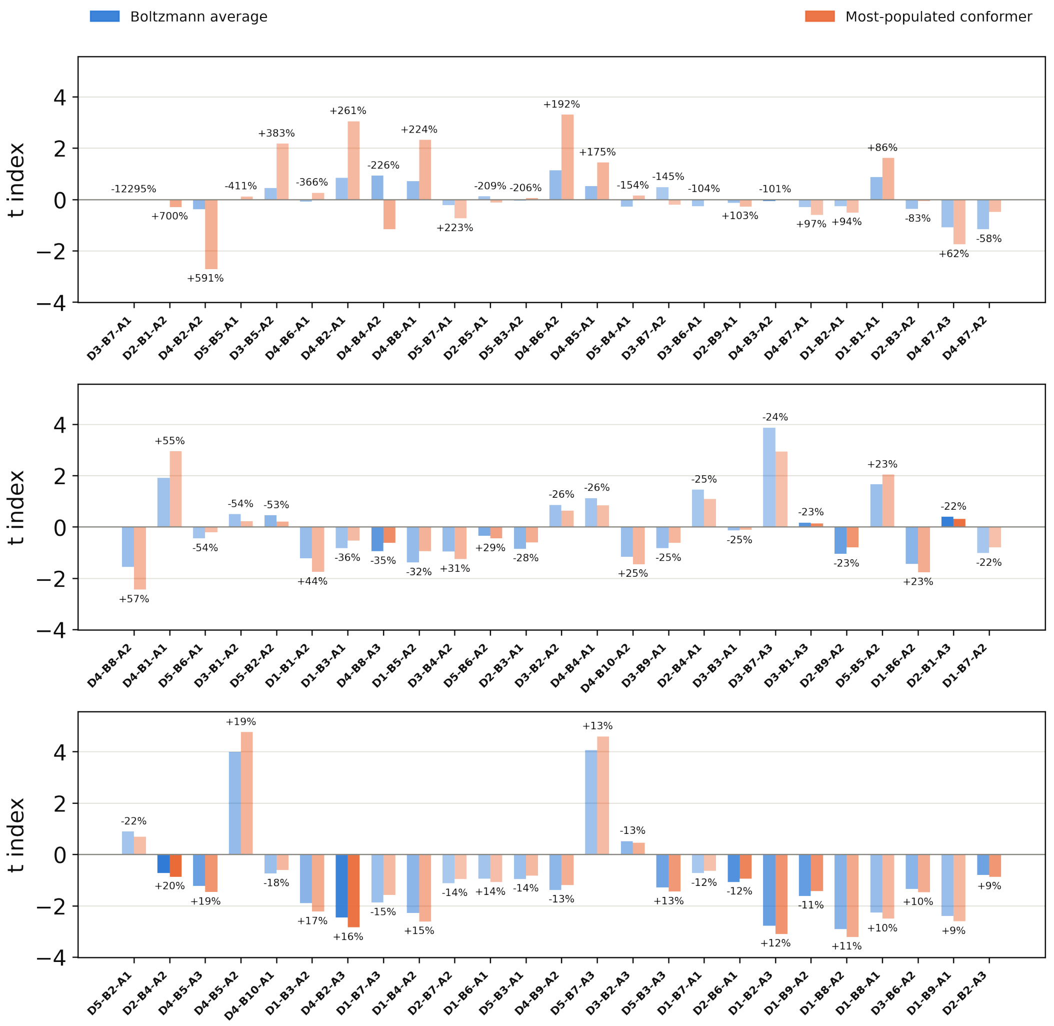

Figure S5: Boltzmann-averaged t index versus the value obtained from only the most-populated conformer of each dye, at the CAM-B3LYP/6-31G(d,p)//r^{2}SCAN-3c gas-phase level. Bar opacity is proportional to the Boltzmann weight of the most-populated conformer (darker = a single conformer dominates the ensemble; lighter = the population is spread across several conformers). The percentage above each pair is the deviation of the most-populated-conformer value relative to the Boltzmann average, $\Delta t = (t^{\text{best}} - \langle t \rangle)/\langle t \rangle$ (large percentages can result from a Boltzmann average close to zero rather than a large absolute change). Dyes are sorted by $|\Delta\%|$ in descending order; this figure shows the 75 dyes with the largest deviations.

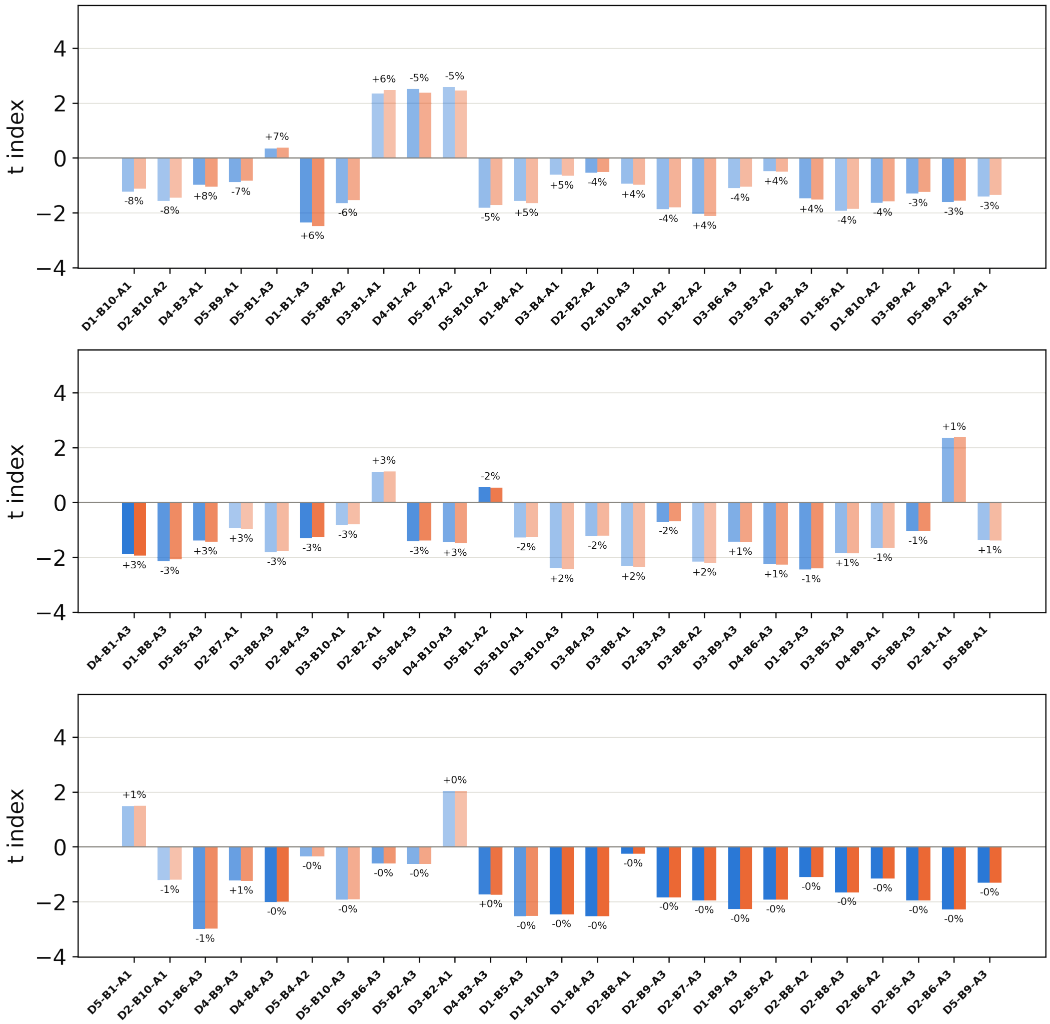

Figure S6: Continuation of Figure S5, showing the remaining 75 (of 150) dyes, in the same order of decreasing $|\Delta\%|$.

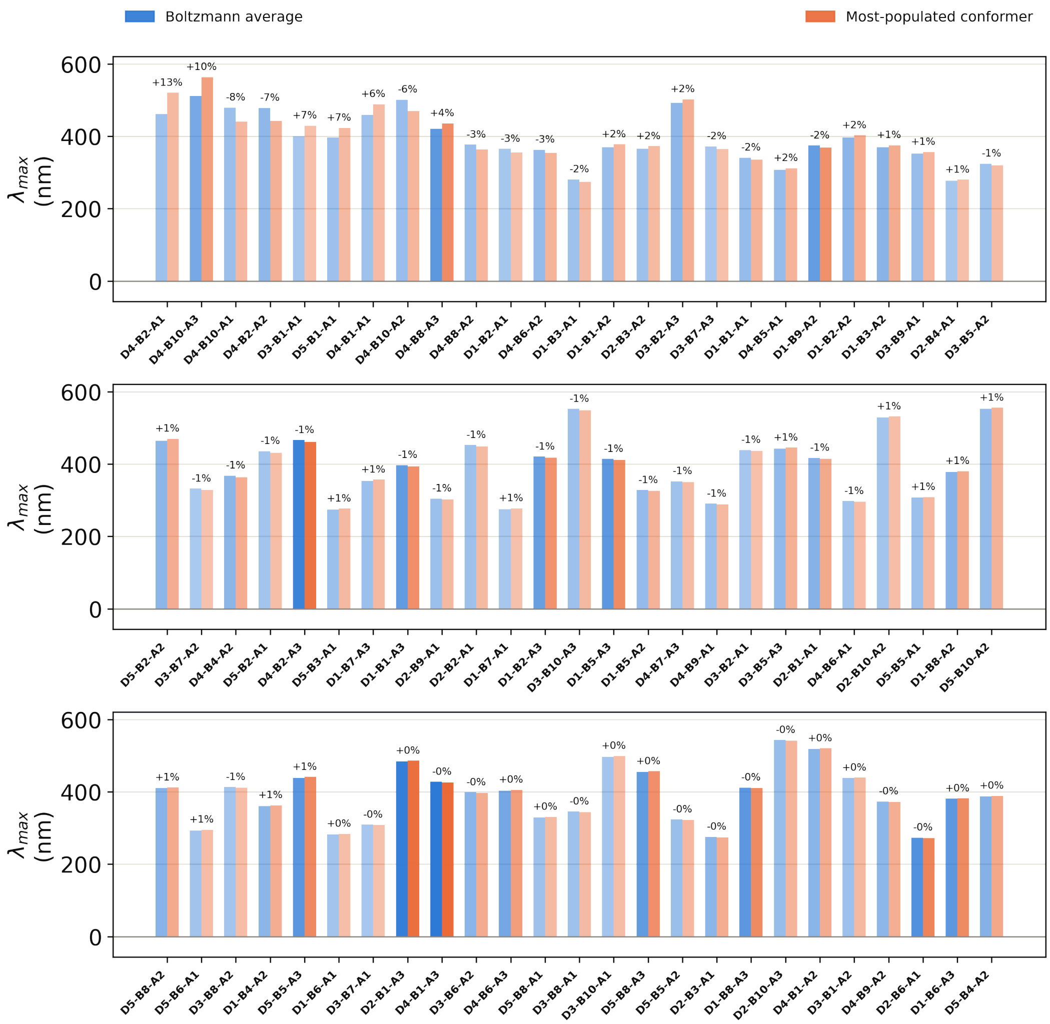

Figure S7: Boltzmann-averaged λ_{max} versus the value obtained from only the most-populated conformer of each dye, at the CAM-B3LYP/6-31G(d,p)//r^{2}SCAN-3c gas-phase level. Bar opacity is proportional to the Boltzmann weight of the most-populated conformer (darker = a single conformer dominates the ensemble; lighter = the population is spread across several conformers). The percentage above each pair is the deviation of the most-populated-conformer value relative to the Boltzmann average, $\Delta\lambda_{\mathrm{max}} = (\lambda_{\mathrm{max}}^{\mathrm{best}} - \langle\lambda_{\mathrm{max}}\rangle)/\langle\lambda_{\mathrm{max}}\rangle$. Dyes are sorted by $|\Delta\%|$ in descending order; this figure shows the 75 dyes with the largest deviations.

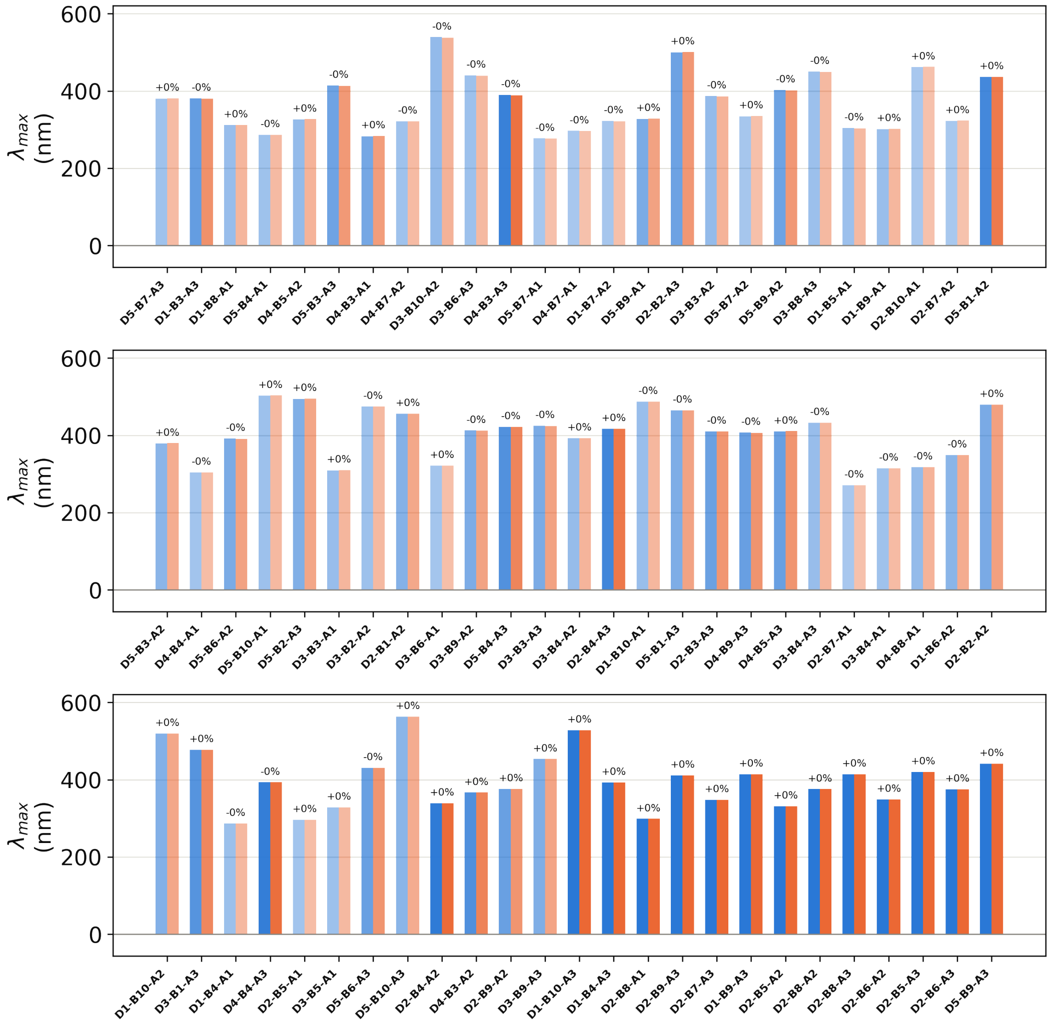

Figure S8: Continuation of Figure S7, showing the remaining 75 (of 150) dyes, in the same order of decreasing $|\Delta\%|$.

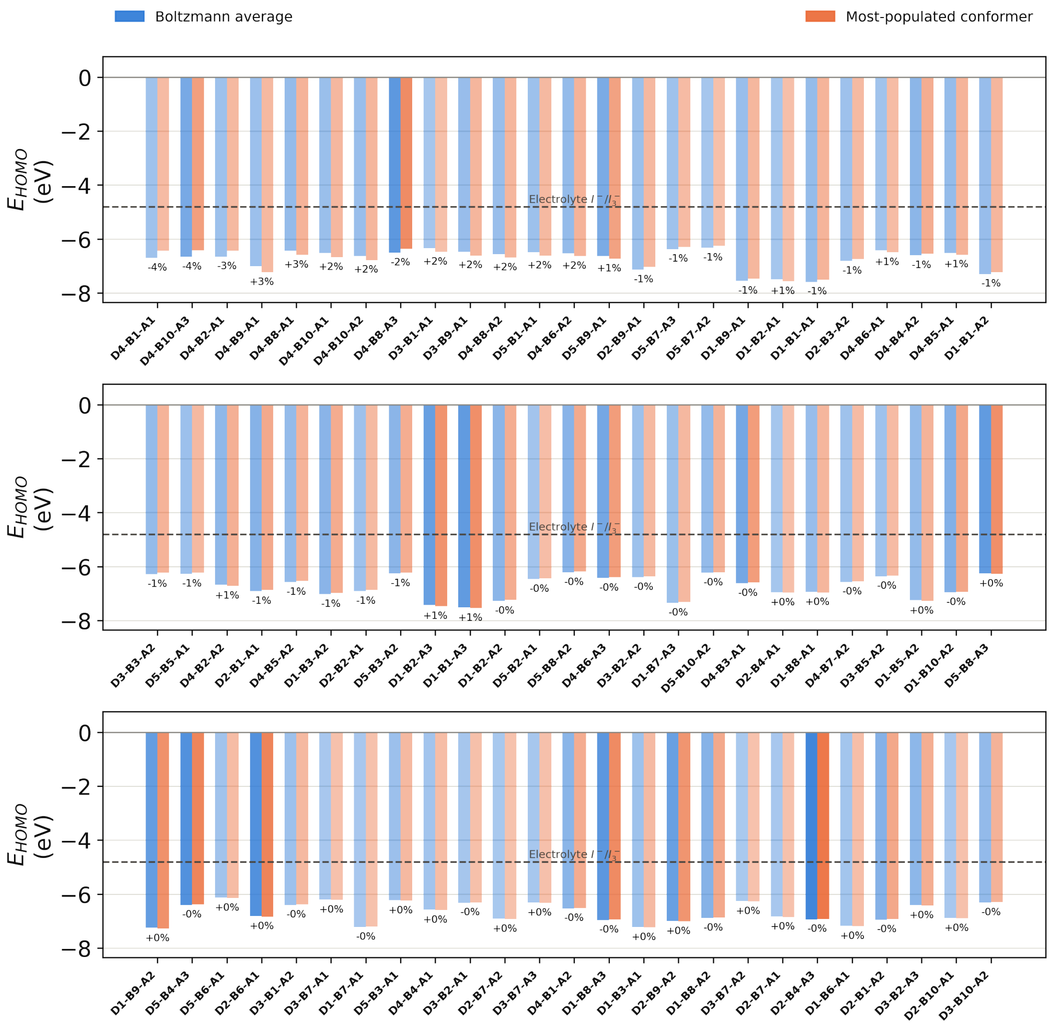

Figure S9: Boltzmann-averaged E_{HOMO} versus the value obtained from only the most-populated conformer of each dye, at the CAM-B3LYP/6-31G(d,p)//r^{2}SCAN-3c gas-phase level. Bar opacity is proportional to the Boltzmann weight of the most-populated conformer (darker = a single conformer dominates the ensemble; lighter = the population is spread across several conformers). The percentage above (or below) each pair is the deviation of the most-populated-conformer value relative to the Boltzmann average, $\Delta E_{\mathrm{HOMO}} = (E^{\mathrm{best}}_{\mathrm{HOMO}} - \langle E_{\mathrm{HOMO}} \rangle)/\langle E_{\mathrm{HOMO}} \rangle$. The dashed line marks the I^-/I_3^- electrolyte redox potential (-4.80 eV); a dye's HOMO must lie below this line for dye regeneration to be thermodynamically favorable. Dyes are sorted by $|\Delta\%|$ in descending order; this figure shows the 75 dyes with the largest deviations.

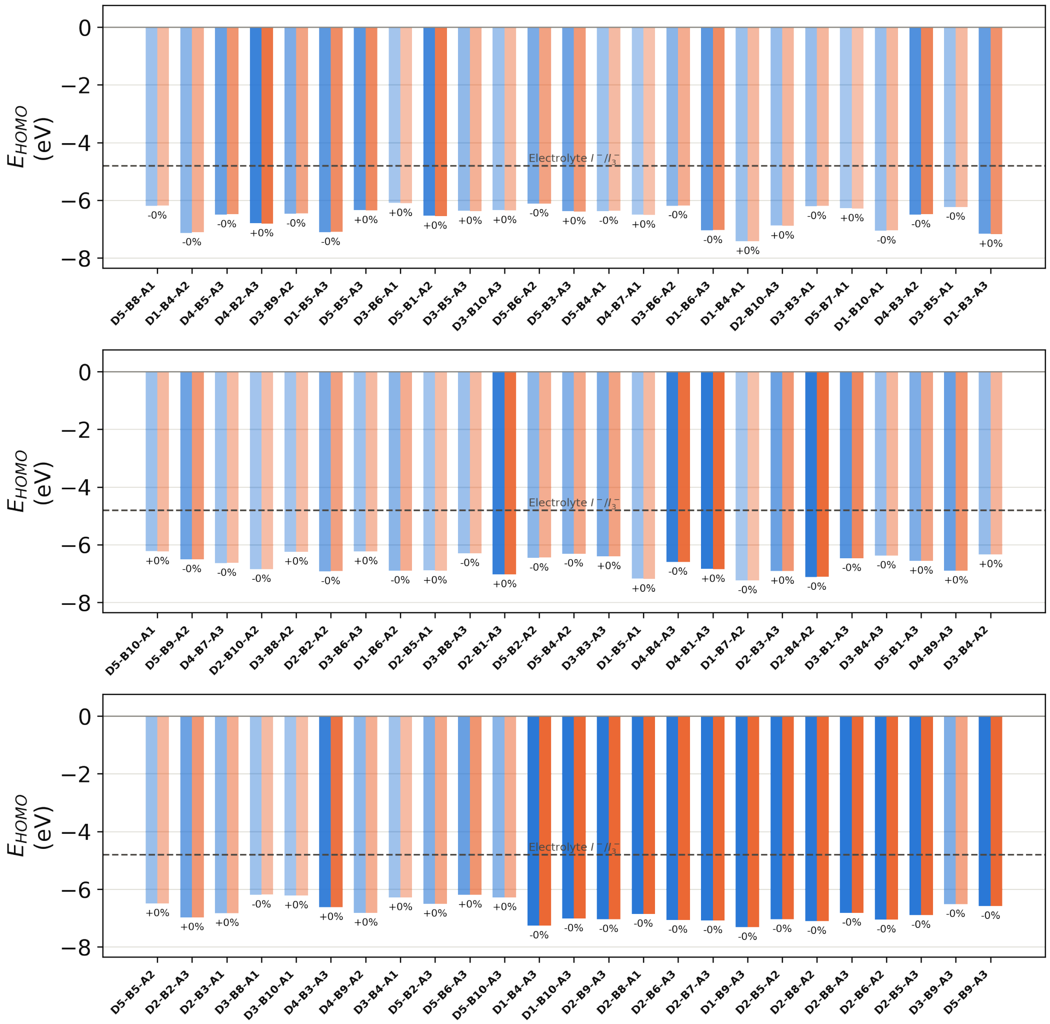

Figure S10: Continuation of Figure S9, showing the remaining 75 (of 150) dyes, in the same order of decreasing $|\Delta\%|$.

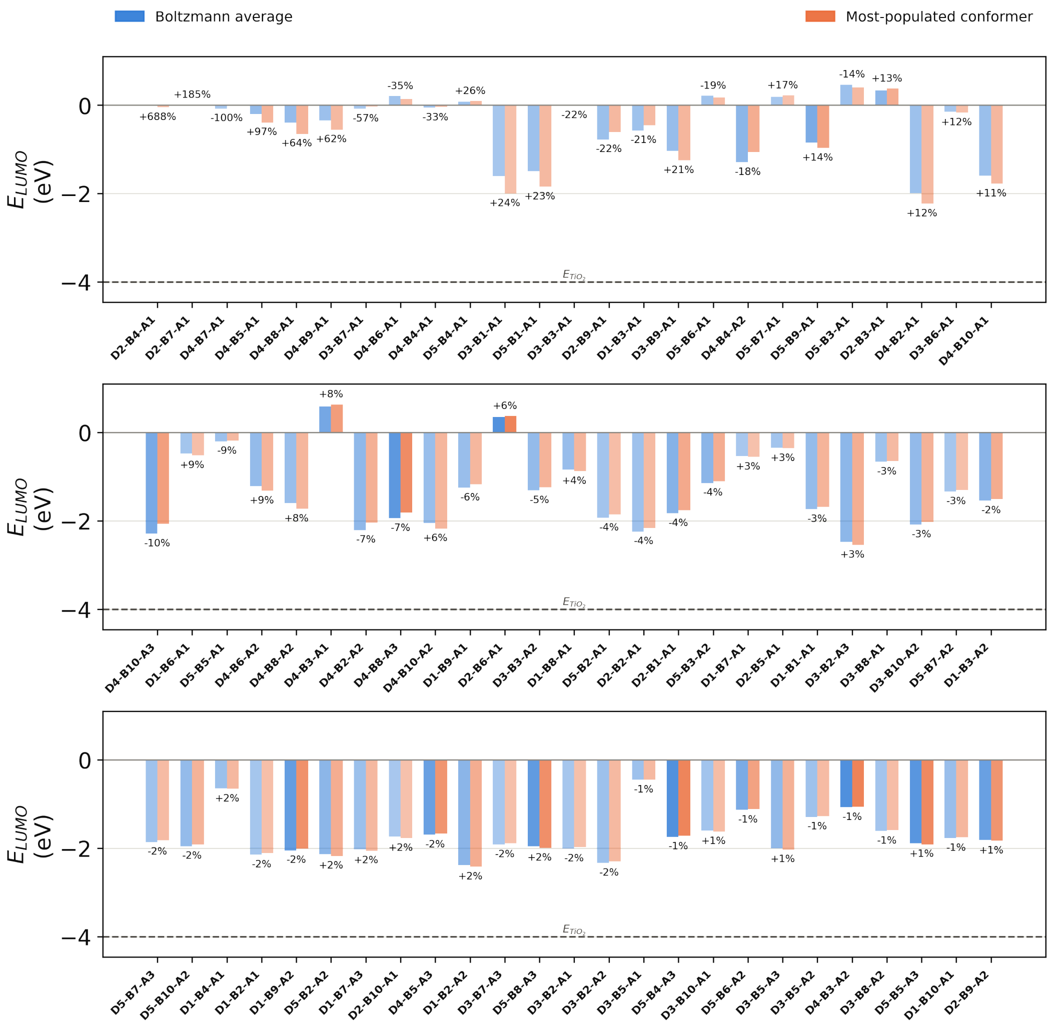

Figure S11: Boltzmann-averaged E_{LUMO} versus the value obtained from only the most-populated conformer of each dye, at the CAM-B3LYP/6-31G(d,p)//r^{2}SCAN-3c gas-phase level. Bar opacity is proportional to the Boltzmann weight of the most-populated conformer (darker = a single conformer dominates the ensemble; lighter = the population is spread across several conformers). The percentage above (or below) each pair is the deviation of the most-populated-conformer value relative to the Boltzmann average, $\Delta E_{\text{LUMO}} = (E^{\text{best}}_{\text{LUMO}} - \langle E_{\text{LUMO}} \rangle)/\langle E_{\text{LUMO}} \rangle$ (large percentages can result from a Boltzmann average close to zero rather than a large absolute change). The dashed line marks the TiO_2 conduction-band edge (−4.00 eV); a dye's LUMO must lie above this line for electron injection to be thermodynamically favorable. Dyes are sorted by $|\Delta\%|$ in descending order; this figure shows the 75 dyes with the largest deviations.

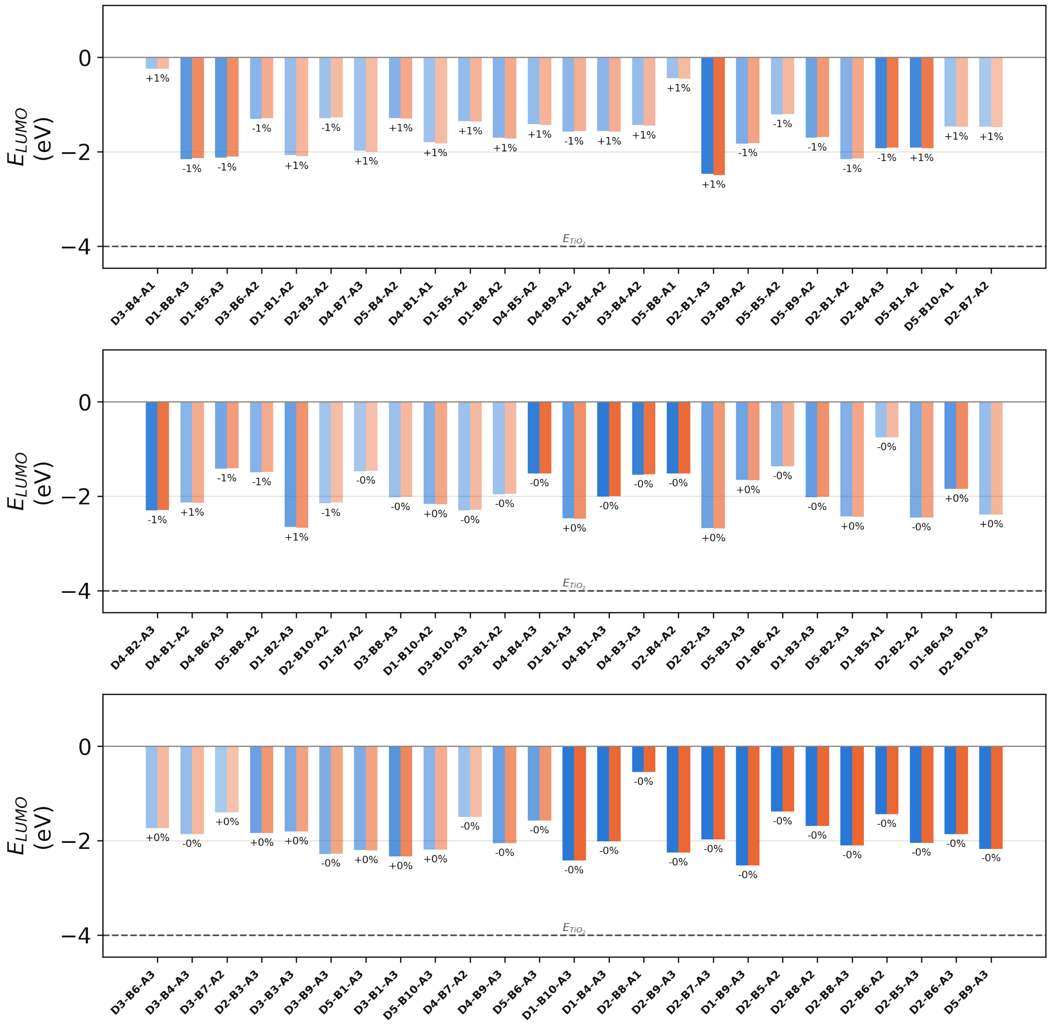

Figure S12: Continuation of Figure S11, showing the remaining 75 (of 150) dyes, in the same order of decreasing $|\Delta\%|$.

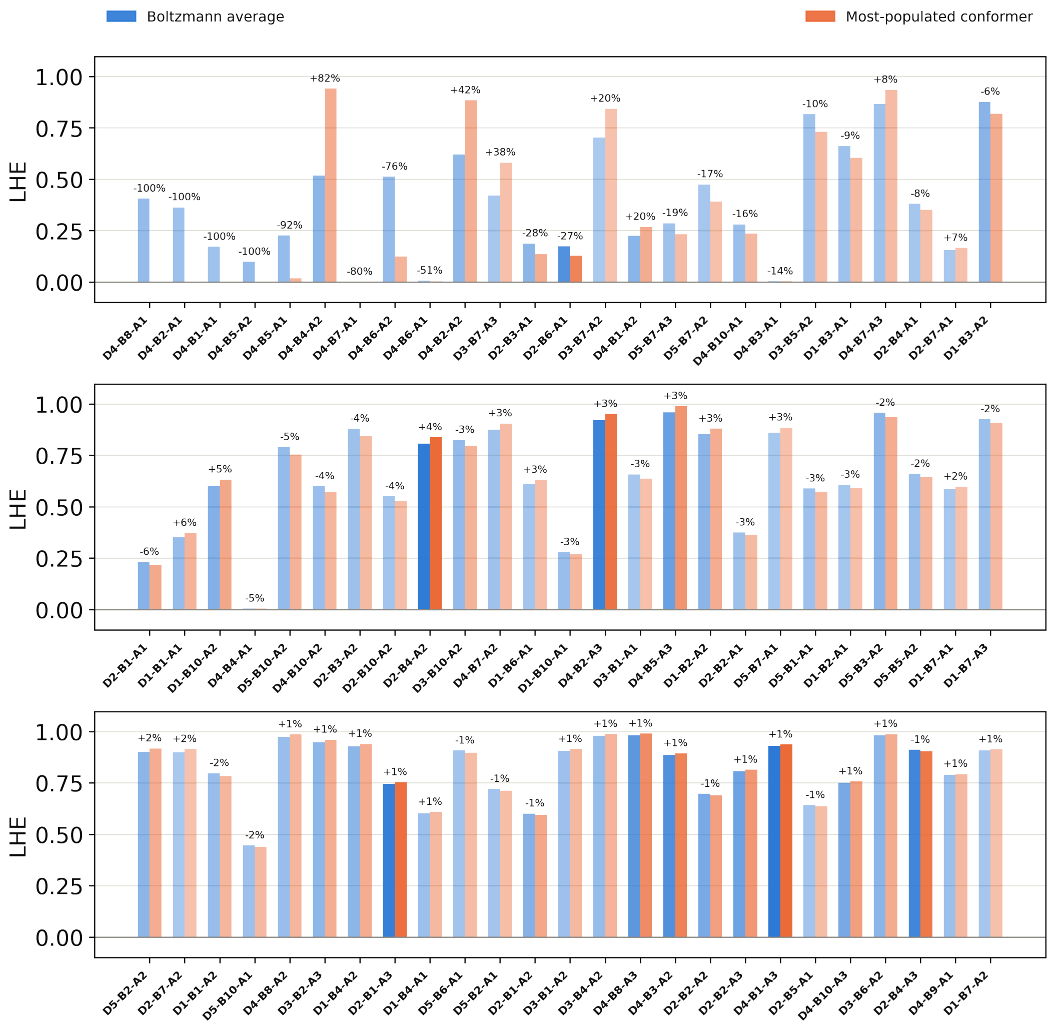

Figure S13: Boltzmann-averaged LHE versus the value obtained from only the most-populated conformer of each dye, at the CAM-B3LYP/6-31G(d,p)//r^{2}SCAN-3c gas-phase level. Bar opacity is proportional to the Boltzmann weight of the most-populated conformer (darker = a single conformer dominates the ensemble; lighter = the population is spread across several conformers). The percentage above each pair is the deviation of the most-populated-conformer value relative to the Boltzmann average, $\Delta\text{LHE} = (\text{LHE}^{\text{best}} - \langle\text{LHE}\rangle)/\langle\text{LHE}\rangle$. Dyes are sorted by $|\Delta\%|$ in descending order; this figure shows the 75 dyes with the largest deviations.

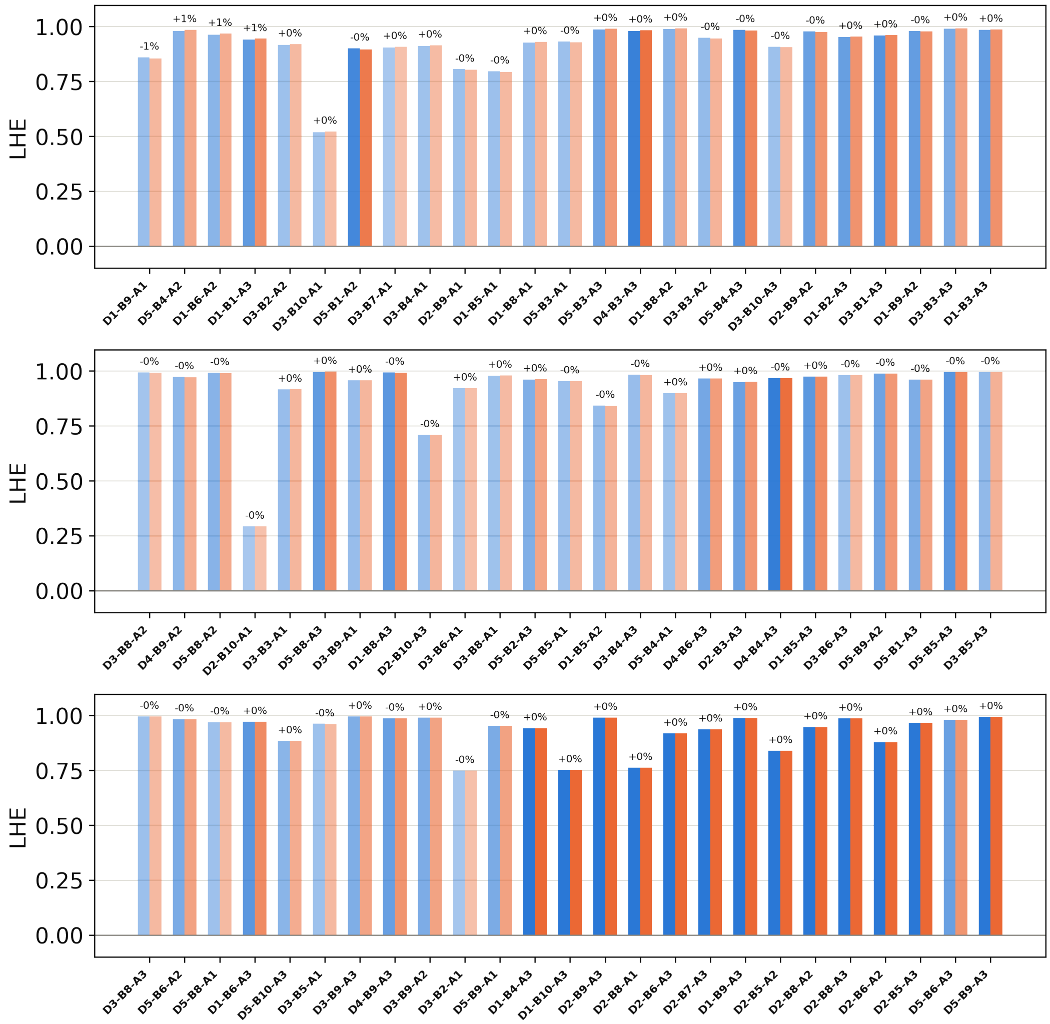

Figure S14: Continuation of Figure S13, showing the remaining 75 (of 150) dyes, in the same order of decreasing $|\Delta\%|$.

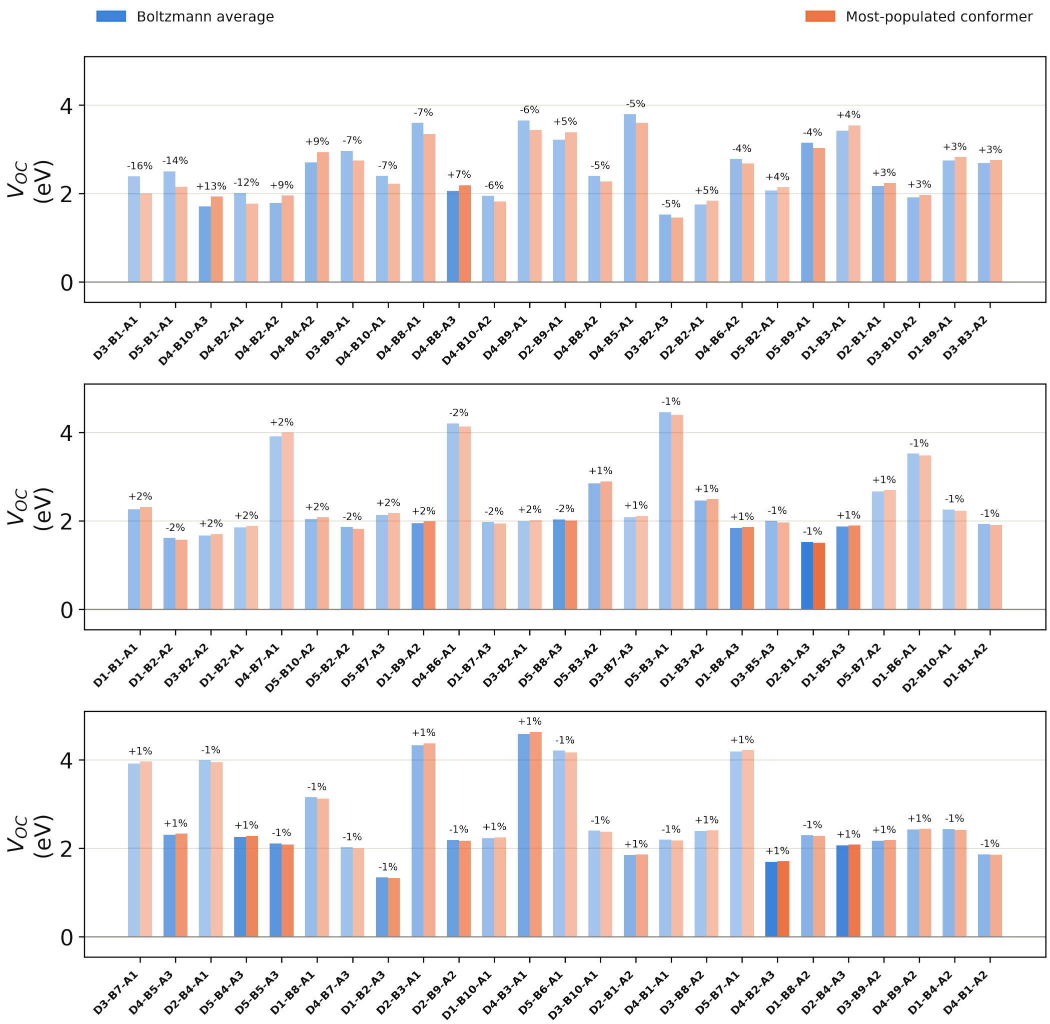

Figure S15: Boltzmann-averaged V_{OC} versus the value obtained from only the most-populated conformer of each dye, at the CAM-B3LYP/6-31G(d,p)//r^{2}SCAN-3c gas-phase level. Bar opacity is proportional to the Boltzmann weight of the most-populated conformer (darker = a single conformer dominates the ensemble; lighter = the population is spread across several conformers). The percentage above each pair is the deviation of the most-populated-conformer value relative to the Boltzmann average, $\Delta V_{OC} = (V_{OC}^{\text{best}} - \langle V_{OC}\rangle)/\langle V_{OC}\rangle$. Dyes are sorted by $|\Delta\%|$ in descending order; this figure shows the 75 dyes with the largest deviations.

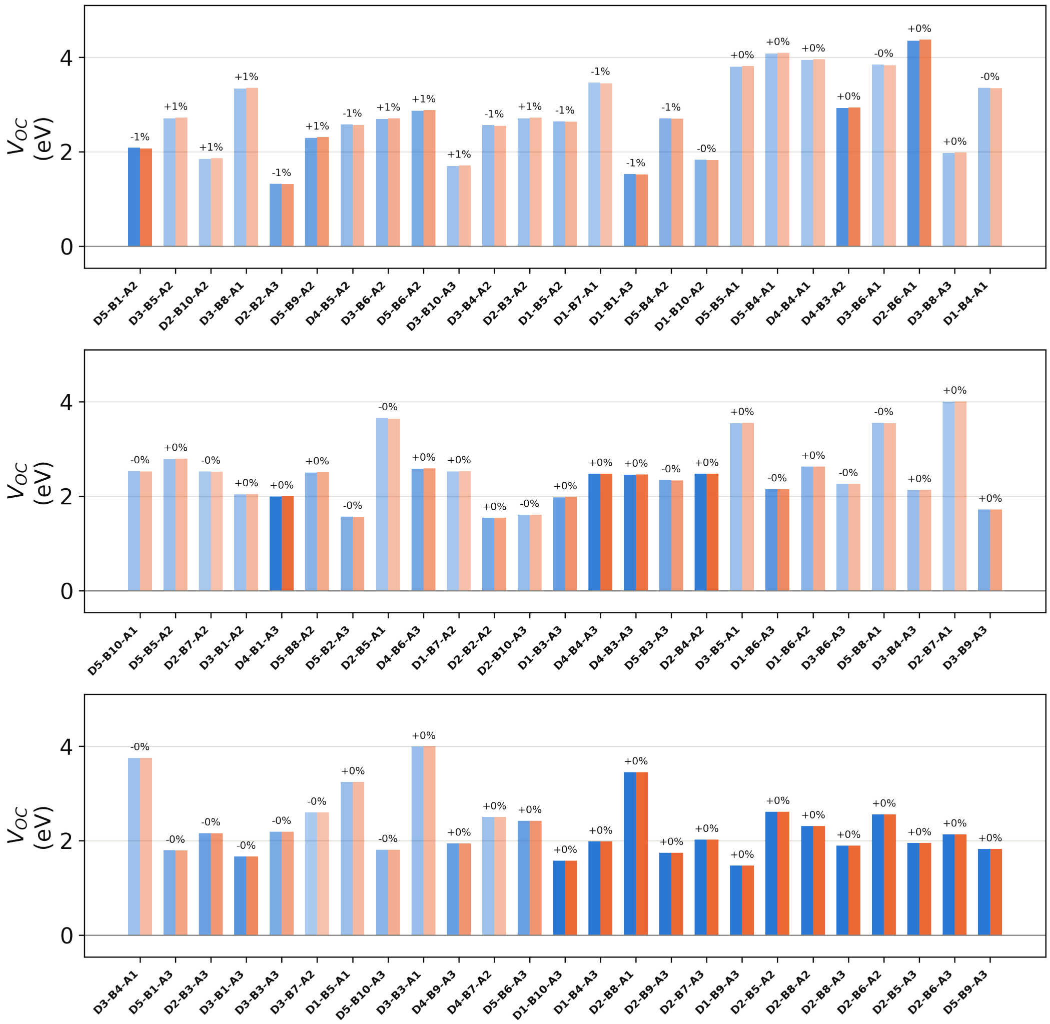

Figure S16: Continuation of Figure S15, showing the remaining 75 (of 150) dyes, in the same order of decreasing $|\Delta\%|$.

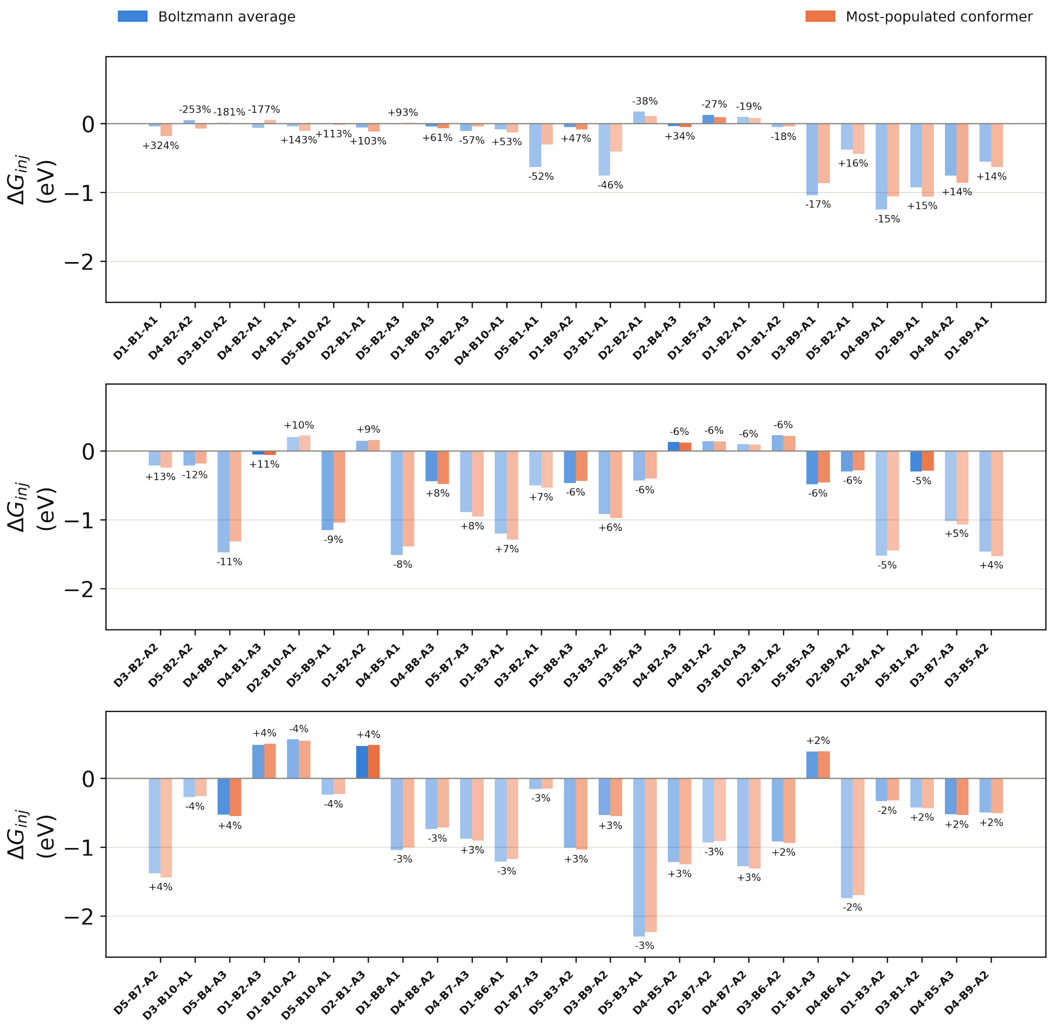

Figure S17: Boltzmann-averaged ΔG_{inj} versus the value obtained from only the most-populated conformer of each dye, at the CAM-B3LYP/6-31G(d,p)//r^{2}SCAN-3c gas-phase level. Bar opacity is proportional to the Boltzmann weight of the most-populated conformer (darker = a single conformer dominates the ensemble; lighter = the population is spread across several conformers). The percentage above (or below) each pair is the deviation of the most-populated-conformer value relative to the Boltzmann average, $\Delta(\Delta G_{\text{inj}}) = (\Delta G_{\text{inj}}^{\text{best}} - \langle \Delta G_{\text{inj}} \rangle)/\langle \Delta G_{\text{inj}} \rangle$ (large percentages can result from a Boltzmann average close to zero rather than a large absolute change). Dyes are sorted by $|\Delta\%|$ in descending order; this figure shows the 75 dyes with the largest deviations.

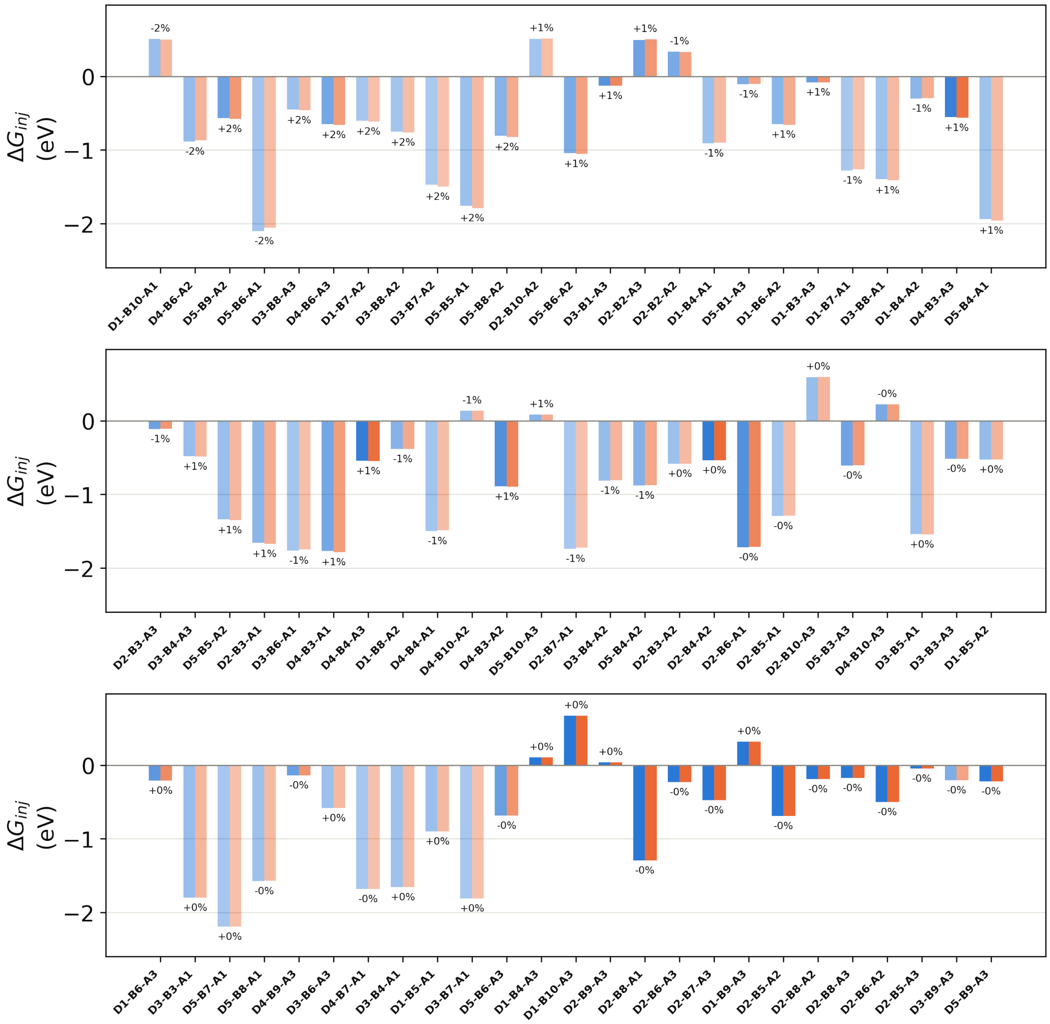

Figure S18: Continuation of Figure S17, showing the remaining 75 (of 150) dyes, in the same order of decreasing $|\Delta\%|$.

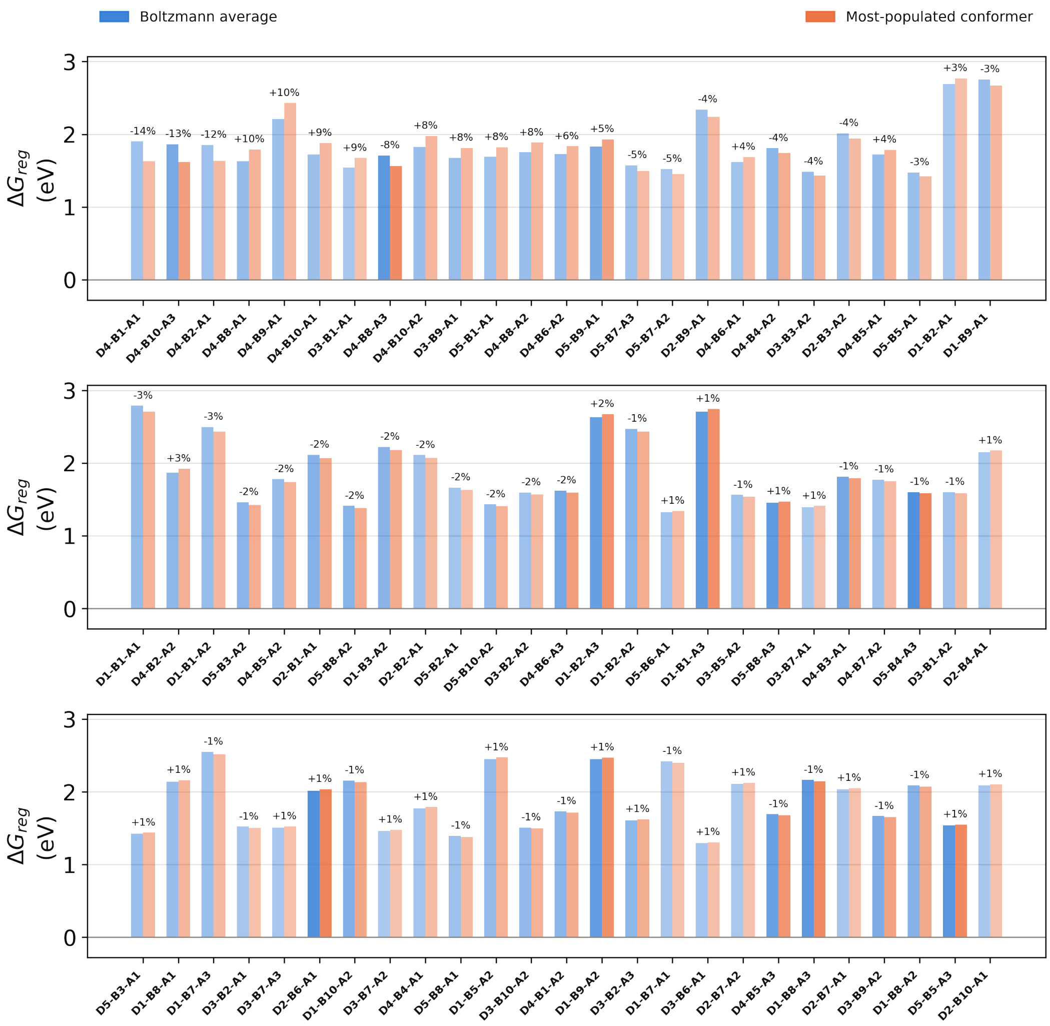

Figure S19: Boltzmann-averaged ΔG_{reg} versus the value obtained from only the most-populated conformer of each dye, at the CAM-B3LYP/6-31G(d,p)//r^{2}SCAN-3c gas-phase level. Bar opacity is proportional to the Boltzmann weight of the most-populated conformer (darker = a single conformer dominates the ensemble; lighter = the population is spread across several conformers). The percentage above each pair is the deviation of the most-populated-conformer value relative to the Boltzmann average, $\Delta(\Delta G_{\text{reg}}) = (\Delta G_{\text{reg}}^{\text{best}} - \langle \Delta G_{\text{reg}} \rangle)/\langle \Delta G_{\text{reg}} \rangle$. Dyes are sorted by $|\Delta\%|$ in descending order; this figure shows the 75 dyes with the largest deviations.

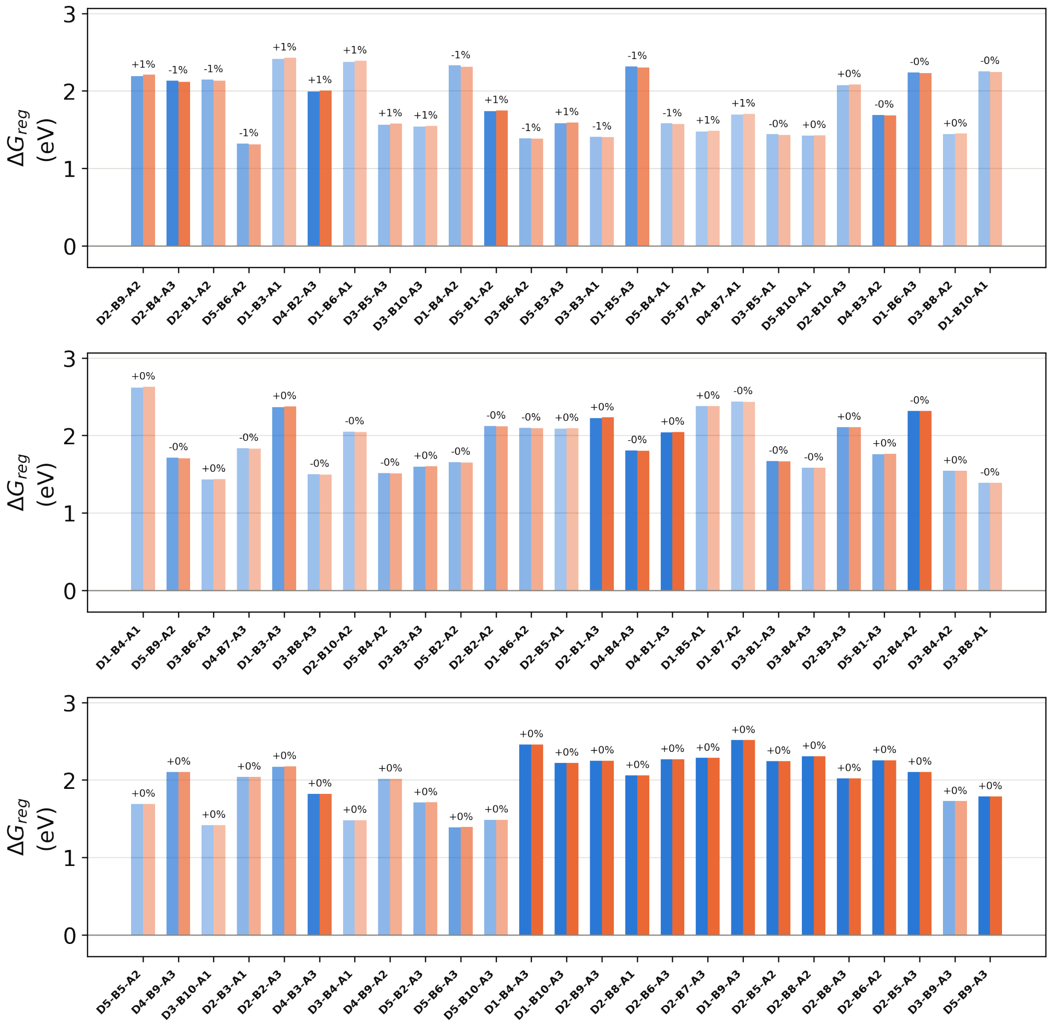

Figure S20: Continuation of Figure S19, showing the remaining 75 (of 150) dyes, in the same order of decreasing $|\Delta\%|$.

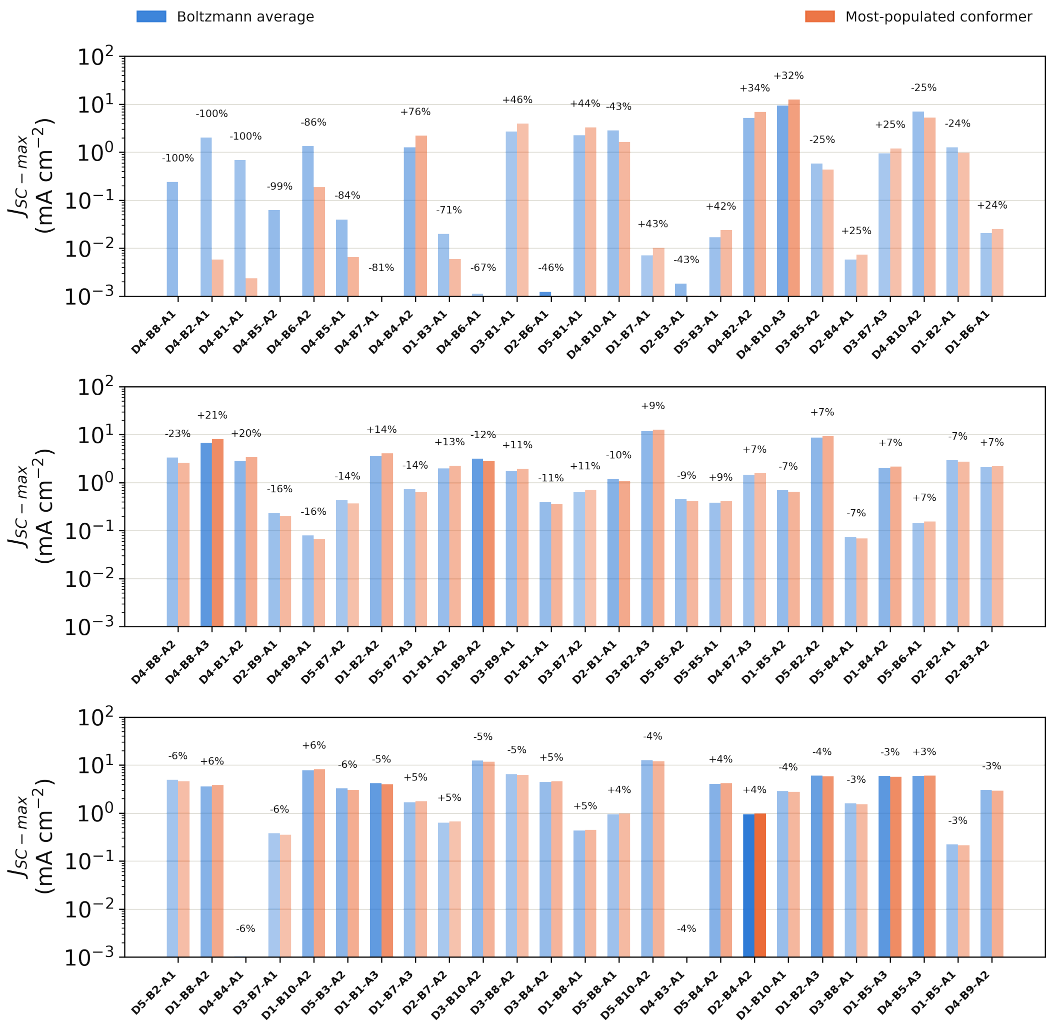

Figure S21: Boltzmann-averaged J_{SC-max} versus the value obtained from only the most-populated conformer of each dye, at the CAM-B3LYP/6-31G(d,p)//r^{2}SCAN-3c gas-phase level. Bar opacity is proportional to the Boltzmann weight of the most-populated conformer (darker = a single conformer dominates the ensemble; lighter = the population is spread across several conformers). The percentage above each pair is the deviation of the most-populated-conformer value relative to the Boltzmann average, $\Delta J_{SC-max} = (J^{\text{best}}_{SC-max} - \langle J_{SC-max}\rangle)/\langle J_{SC-max}\rangle$. Dyes are sorted by $|\Delta\%|$ in descending order; this figure shows the 75 dyes with the largest deviations.

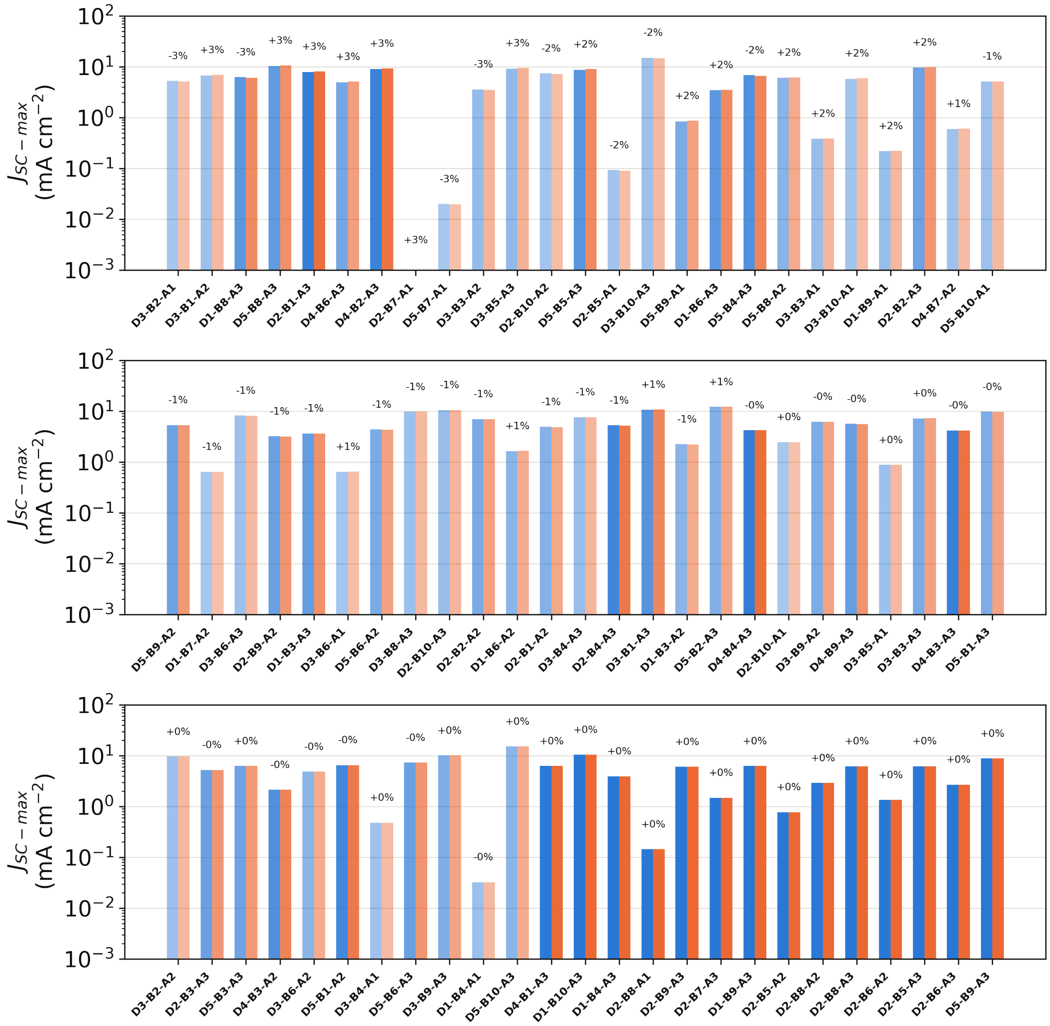

Figure S22: Continuation of Figure S21, showing the remaining 75 (of 150) dyes, in the same order of decreasing $|\Delta\%|$.

2.7 Simulated UV-Vis Absorption Spectra: Most-Populated Conformer versus Ensemble Average

For each of the ten top-ranked sensitizers, at each of the three functionals, Figures S23 and S24 compare the simulated absorption spectrum of the single most-populated conformer (dashed orange) with the Boltzmann-weighted conformational-ensemble average (solid blue, filled), together with oscillator-strength stick markers for the most-populated conformer's twelve computed states (the $S_0 \rightarrow S_1$ transition is marked with a circle). Each spectrum was obtained by Gaussian broadening of the computed oscillator strengths using the standard Gaussian 16 convention, $\varepsilon(\tilde{\nu}) = 1.3062974 \times 10^8\,(f/\mathrm{HWHM})\,\exp[-((\tilde{\nu}-\tilde{\nu}_i)/\mathrm{HWHM})^2]$, with a half-width at half maximum of 0.333 eV (2685.83 cm^{-1}, the Gaussian 16 default). The ensemble-average spectrum is the population-weighted sum of every individual conformer's own twelve-state Gaussian-broadened spectrum, not a spectrum built from population-

averaged state parameters.

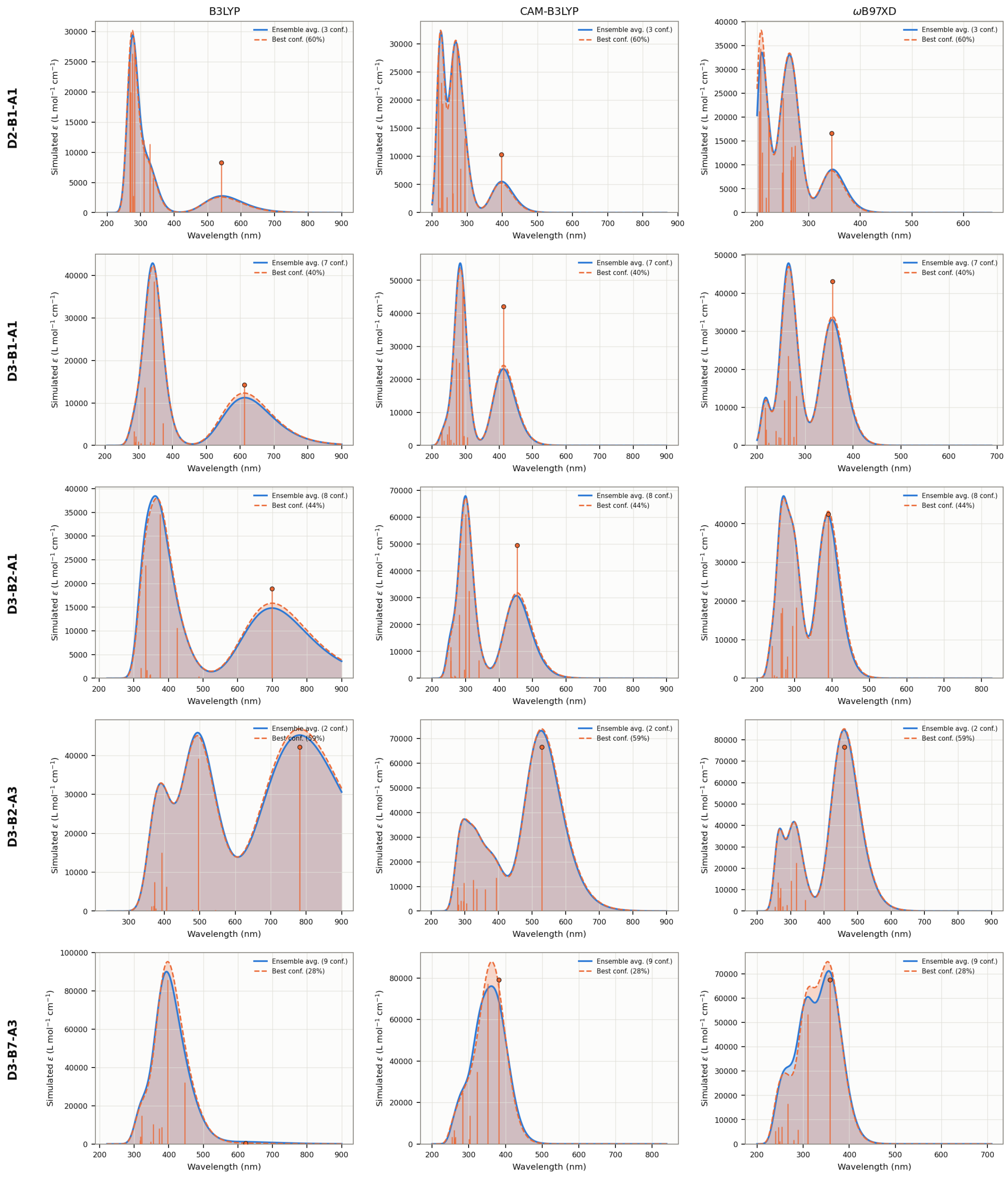

Figure S23: Simulated UV-Vis absorption spectra of D2-B1-A1, D3-B1-A1, D3-B2-A1, D3-B2-A3, and D3-B7-A3, most-populated conformer (dashed orange) versus Boltzmann-weighted ensemble average (solid blue), at B3LYP, CAM-B3LYP, and ωB97XD (SMD, acetonitrile). Vertical orange lines mark the oscillator strength of each of the most-populated conformer's twelve computed states, scaled to the intensity axis; the circle marks the $S_0 \rightarrow S_1$ transition.

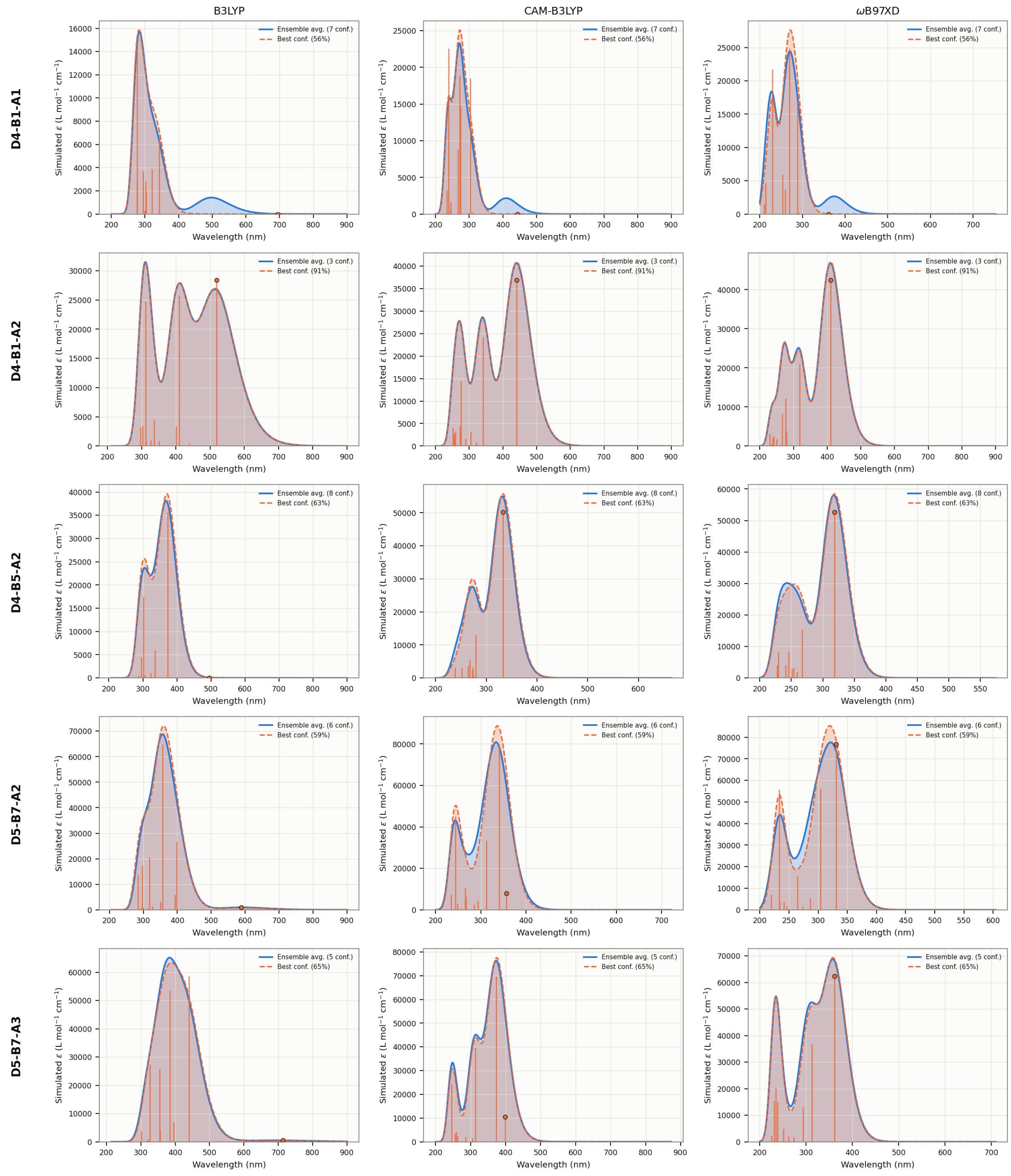

Figure S24: Simulated UV-Vis absorption spectra of D4-B1-A1, D4-B1-A2, D4-B5-A2, D5-B7-A2, and D5-B7-A3, most-populated conformer (dashed orange) versus Boltzmann-weighted ensemble average (solid blue), at B3LYP, CAM-B3LYP, and ωB97XD (SMD, acetonitrile). Vertical orange lines mark the oscillator strength of each of the most-populated conformer's twelve computed states, scaled to the intensity axis; the circle marks the $S_0 \rightarrow S_1$ transition.

2.8 Solvent-Phase Conformer-Averaging Analysis for the Top-10 Sensitizers (SMD(Acetonitrile), r^2SCAN-3c Geometries, TD-DFT Single Points at Three Functionals)

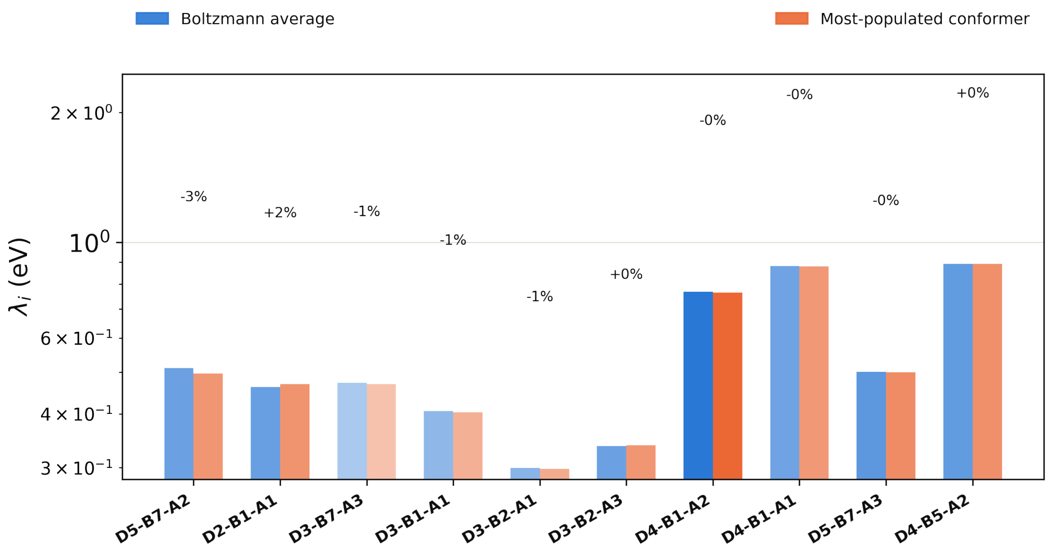

Figure S25: Boltzmann-averaged internal reorganization energy λ_i versus the value obtained from only the most-populated conformer, for the ten top-ranked sensitizers at the CAM-B3LYP/6-31G(d,p)/SMD(acetonitrile) level. Bar opacity is proportional to the Boltzmann weight of the most-populated conformer. The percentage above each pair is the deviation of the most-populated-conformer value relative to the Boltzmann average, $\Delta\lambda_i = (\lambda_i^{\text{best}} - \langle\lambda_i\rangle)/\langle\lambda_i\rangle$.

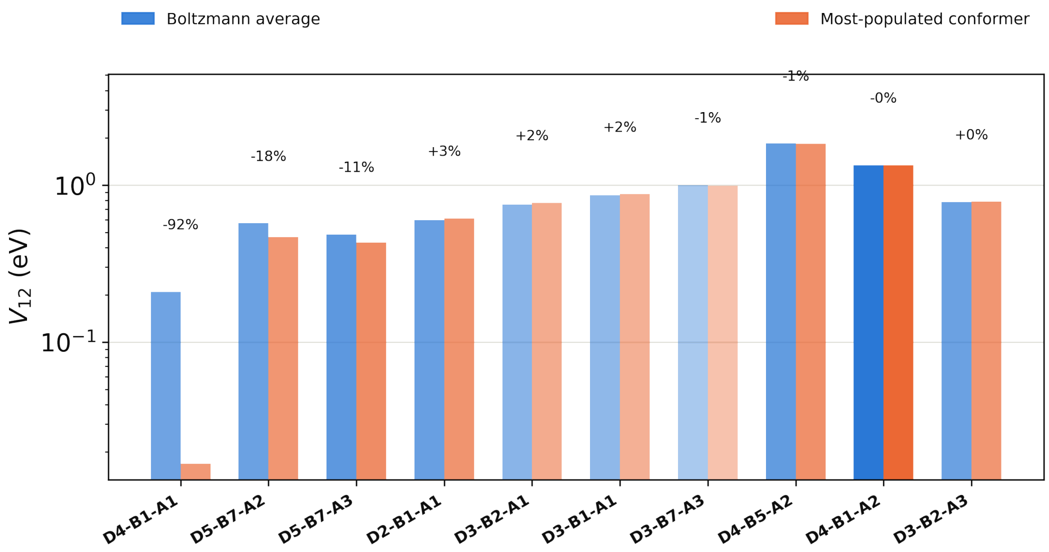

Figure S26: Boltzmann-averaged electronic coupling V_{12} versus the value obtained from only the most-populated conformer, for the ten top-ranked sensitizers at the CAM-B3LYP/6-31G(d,p)/SMD(acetonitrile) level. Bar opacity is proportional to the Boltzmann weight of the most-populated conformer. The percentage above each pair is the deviation of the most-populated-conformer value relative to the Boltzmann average, $\Delta V_{12} = (V_{12}^{\text{best}} - \langle V_{12} \rangle)/\langle V_{12} \rangle$.

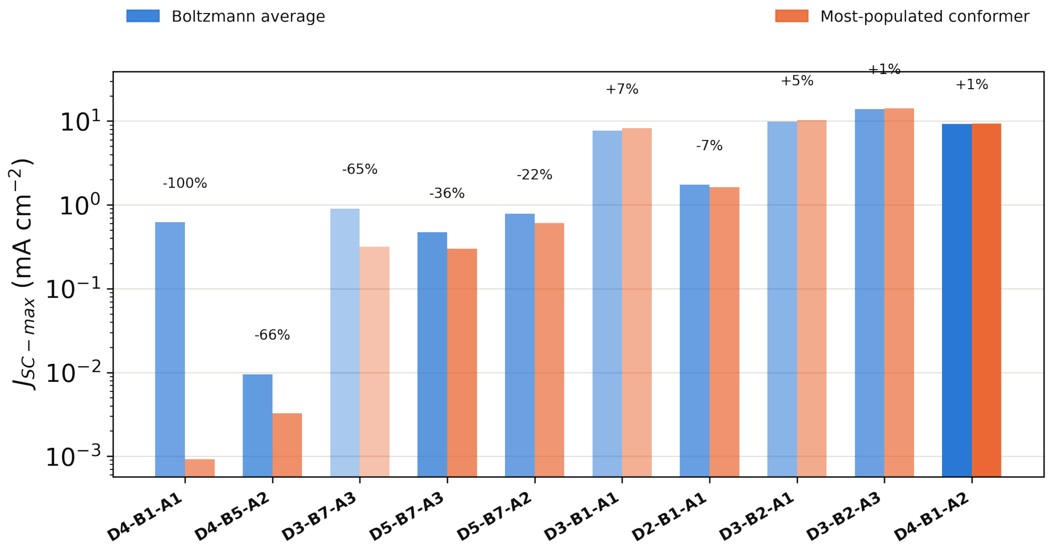

Figure S27: Comparison between Boltzmann-averaged and most-populated-conformer $J_{\text{SC}-\text{max}}$ for the ten top-ranked sensitizers at the B3LYP/6-31G(d,p)/SMD(acetonitrile) level. The percentage above each pair of bars represents the relative deviation of the most-populated-conformer value from the Boltzmann average, $\Delta J_{\text{SC}-\text{max}} = (J_{\text{SC}-\text{max}}^{\text{best}} - \langle J_{\text{SC}-\text{max}} \rangle)/\langle J_{\text{SC}-\text{max}} \rangle$. Bar opacity is proportional to the Boltzmann weight of the most-populated conformer (darker = a single conformer dominates the ensemble; lighter = the population is spread across several conformers).

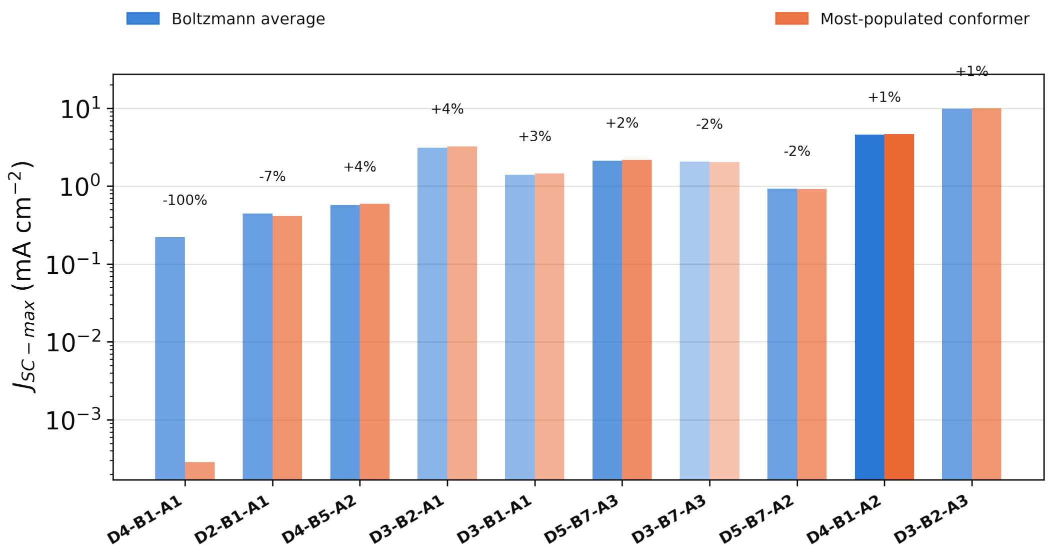

Figure S28: Comparison between Boltzmann-averaged and most-populated-conformer $J_{\mathrm{SC-max}}$ for the ten top-ranked sensitizers at the ωB97XD/6-31G(d,p)/SMD(acetonitrile) level. The percentage above each pair of bars represents the relative deviation of the most-populated-conformer value from the Boltzmann average, $\Delta J_{\mathrm{SC-max}} = (J^{\mathrm{best}}_{\mathrm{SC-max}} - \langle J_{\mathrm{SC-max}} \rangle)/\langle J_{\mathrm{SC-max}} \rangle$. Bar opacity is proportional to the Boltzmann weight of the most-populated conformer (darker = a single conformer dominates the ensemble; lighter = the population is spread across several conformers).

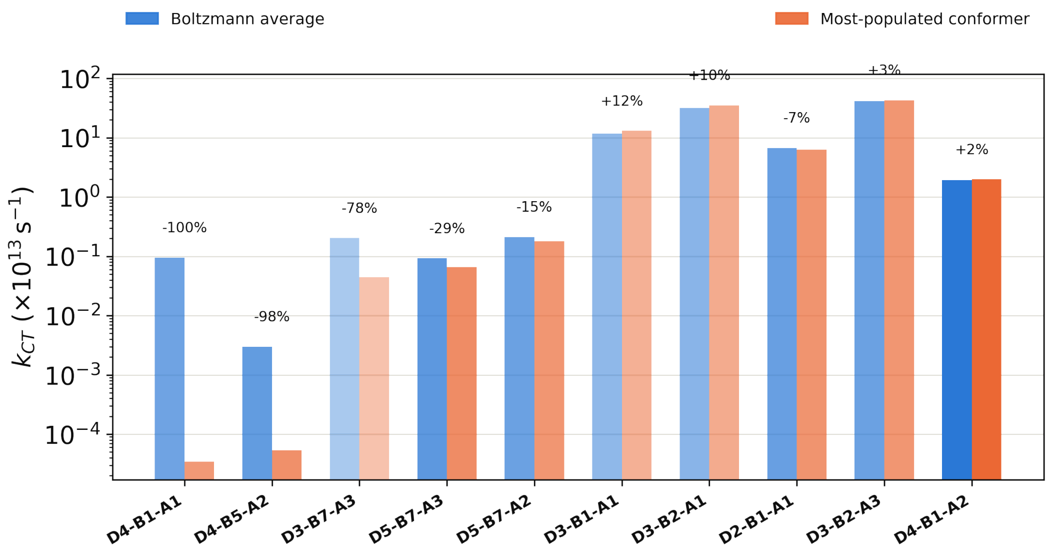

Figure S29: Comparison between Boltzmann-averaged and most-populated-conformer charge-transfer rate constants, k_{CT}, for the ten top-ranked sensitizers at the B3LYP/6-31G(d,p)/SMD(acetonitrile) level. The percentage above each pair of bars represents the relative deviation of the most-populated-conformer value from the Boltzmann average, $\Delta k_{\mathrm{CT}} = (k_{\mathrm{CT}}^{\mathrm{best}} - \langle k_{\mathrm{CT}} \rangle)/\langle k_{\mathrm{CT}} \rangle$. Bar opacity is proportional to the Boltzmann weight of the most-populated conformer (darker = a single conformer dominates the ensemble; lighter = the population is spread across several conformers).

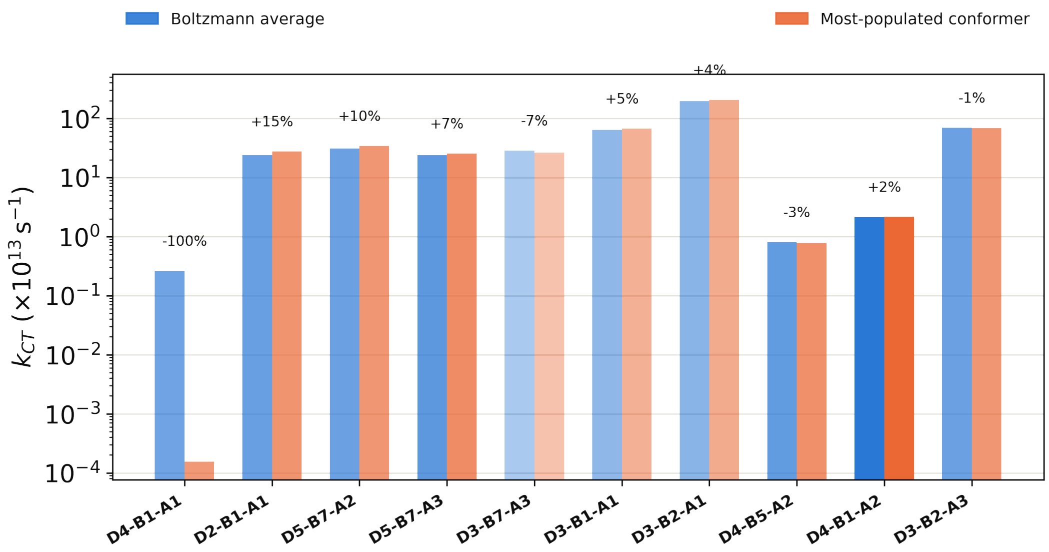

Figure S30: Comparison between Boltzmann-averaged and most-populated-conformer charge-transfer rate constants, k_{CT}, for the ten top-ranked sensitizers at the ωB97XD/6-31G(d,p)/SMD(acetonitrile) level. The percentage above each pair of bars represents the relative deviation of the most-populated-conformer value from the Boltzmann average, $\Delta k_{\mathrm{CT}} = (k_{\mathrm{CT}}^{\mathrm{best}} - \langle k_{\mathrm{CT}} \rangle)/\langle k_{\mathrm{CT}} \rangle$. Bar opacity is proportional to the Boltzmann weight of the most-populated conformer (darker = a single conformer dominates the ensemble; lighter = the population is spread across several conformers).
